# Diversity patterns of bacteriophages infecting *Aggregatibacter* and *Haemophilus* species across clades and niches

**DOI:** 10.1038/s41396-019-0450-8

**Published:** 2019-06-14

**Authors:** Szymon P. Szafrański, Mogens Kilian, Ines Yang, Gesa Bei der Wieden, Andreas Winkel, Jan Hegermann, Meike Stiesch

**Affiliations:** 1 Department of Prosthetic Dentistry and Biomedical Materials Science, Hannover Medical School (MHH), Hannover, Germany; 2 Lower Saxony Centre for Biomedical Engineering, Implant Research and Development (NIFE), Hannover, Germany; 3 Cluster of Excellence RESIST (EXC 2155), Hannover, Germany; 4 Department of Biomedicine, Faculty of Health, Aarhus University, Aarhus, Denmark; 5 Research Core Unit Electron Microscopy, Hannover Medical School (MHH), Hannover, Germany; 6 Cluster of Excellence REBIRTH (EXC 62), Hannover, Germany

**Keywords:** Bacteriophages, Bacterial genetics, Phylogenetics, Microbial ecology, Biodiversity

## Abstract

*Aggregatibacter* and *Haemophilus* species are relevant human commensals and opportunistic pathogens. Consequently, their bacteriophages may have significant impact on human microbial ecology and pathologies. Our aim was to reveal the prevalence and diversity of bacteriophages infecting *Aggregatibacter* and *Haemophilus* species that colonize the human body. Genome mining with comparative genomics, screening of clinical isolates, and profiling of metagenomes allowed characterization of 346 phages grouped in 52 clusters and 18 superclusters. Less than 10% of the identified phage clusters were represented by previously characterized phages. Prophage diversity patterns varied significantly for different phage types, host clades, and environmental niches. A more diverse phage community lysogenizes *Haemophilus influenzae* and *Haemophilus parainfluenzae* strains than *Aggregatibacter actinomycetemcomitans* and “*Haemophilus ducreyi”*. Co-infections occurred more often in *“H. ducreyi”*. Phages from *Aggregatibacter actinomycetemcomitans* preferably lysogenized strains of specific serotype. Prophage patterns shared by subspecies clades of different bacterial species suggest similar ecoevolutionary drivers. Changes in frequencies of DNA uptake signal sequences and guanine–cytosine content reflect phage-host long-term coevolution. *Aggregatibacter* and *Haemophilus* phages were prevalent at multiple oral sites. Together, these findings should help exploring the ecoevolutionary forces shaping virus-host interactions in the human microbiome. Putative lytic phages, especially phiKZ-like, may provide new therapeutic options.

## Introduction

*Aggregatibacter* and *Haemophilus* species of the family *Pasteurellaceae* encompass commensals and pathogens of considerable ecological and medical importance [1]. They dominate mucosal surfaces in the oral cavity and pharynx as well as contributing to dental biofilm development [2–5]. *Haemophilus parainfluenzae* is among the most abundant and transcriptionally active species across all oral and pharyngeal sites [3–8]. *Haemophilus influenzae* is the most important human opportunistic pathogen among *Pasteurellaceae* species [1]. Although vaccination against *H. influenzae* serotype b dramatically reduced the burden of invasive *H. influenzae* diseases, unvaccinated children and patients with predisposing conditions are still at risk. *H. influenzae* strains that form no capsule may cause common pediatric infections as well as acute exacerbation of chronic obstructive pulmonary disease in adults. “*Haemophilus ducreyi”* is the cause of chancroid, a sexually transmitted disease characterized by genital ulcerations, and cutaneous ulcers in children [1]. *Aggregatibacter* species have been linked to periodontal disease, infective endocarditis, and extra-oral abscesses [1].

Relatively little is known about evolutionary and ecological drivers that control populations of *Aggregatibacter* and *Haemophilus* species. Bacteriophages may be candidates for such force [9–12]. These bacterial viruses, in short phages, are either obligatory parasites with strictly lytic lifestyle or they also act as mutualists and exhibit a temperate lifestyle [13]. Phage and bacterium exert reciprocal selective pressures on each other leading to rapid adaptation of both of them [12, 14, 15]. On the one hand, phages may decimate a bacterial population or even bring it to extinction; on the other, they may transfer genetic traits improving ecological fitness [10] or mediate defense against other phages [16]. Therefore, phages infecting *Aggregatibacter* and *Haemophilus* species are likely contributors to the control of their host populations and may indirectly influence the human microbiome and potentially human health.

Despite the high medical and ecological importance of *Aggregatibacter* and *Haemophilus* species, the population of phages infecting these species has not been widely explored [17, 18]. Although the first *Haemophilus* phage was reported in early 1960s [19], so far only three types of *H. influenzae* phages have been reported. These were (i) five P2-like phages: HP1, HP2, S2A, S2B, and S2C, (ii) transposable Mu-like FluMu phage, and (iii) unclassified siphovirus N3 [20, 21]. These P2-like and Mu-like phages represent the family *Myoviridae*. Exploration of *Aggregatibacter actinomycetemcomitans* phages started with induction and characterization of lambda-like phiAal7/phiAa phage [22]. Later, dozens of related *Aggregatibacter* myoviruses were detected and genetics of two, Aaphi23 and S1249, were revealed as reviewed in ref. [18]. Although lysogeny has not been linked to clinical conditions, in vitro studies showed that *Aggregatibacter* myoviruses can transfer antibiotic resistance cassettes, induce serotype conversion, and potentially increase release of leukotoxin [18]. *Aggregatibacter* siphoviruses and podoviruses were occasionally observed but have not been characterized. Virulent phages infecting *A. actinomycetemcomitans* or *H. influenzae* have not been reported. Phages for the other human-associated *Aggregatibacter* and *Haemophilus* species are either not known or are represented by unclassified putative phage sequences. Putative virulent phiKZ-like *Haemophilus* phages were detected by metagenomics [23] but this type of phages is also known to show pseudolysogeny [24]. Current taxonomy of *Aggregatibacter* and *Haemophilus* phages curated by the International Committee on Taxonomy of Viruses is limited to only two species: *Haemophilus* virus HP1 and HP2 both classified in genus Hp1virus, subfamily Peduovirinae, family Myoviridae and order Caudovirales (see Table S1 in the Supplementary Information for more details). Thus, prevalence, diversity and genetics of *Aggregatibacter* and *Haemophilus* phages remained largely unknown, and consequently the impact of these phages on the human microbiome could not be fully explored until now.

In the present study, we explored three main datasets to reveal the prevalence and diversity of *Aggregatibacter* and *Haemophilus* phages with focus on temperate, tailed, double-stranded DNA phages. First, we described the prophages found in publicly available genomes of human-associated *Pasteurellaceae* species [25], and established a classification scheme for these phages. Subsequently, we detected prophages in genomes of clinical isolates of *A. actinomycetemcomitans* and obtained full-genome sequences for representative prophages. Finally, we detected phages in publicly available human microbiome metagenomes [26] and classified metagenomic phage assemblies from geographically diverse samples [23, 27]. By linking various metadata to classified phage sequences, we could show how phage diversity differs across clades and niches, and how phages co-evolve with their host. Our data laid the foundation for a comprehensive definition of the population structure of phages infecting *Aggregatibacter* and *Haemophilus* species that can be further used to explore the ecological role of these phages in the human microbiome and develop new therapeutic approaches.

## Results

We screened 276 publicly available genomes of 14 human-associated *Aggregatibacter* and *Haemophilus* species for phage-like elements (Fig. S1a). In total, 828 phage-like elements were discovered, 258 of which were predicted to be intact, coding for at least 40 open reading frames (ORFs), and not duplicated within the dataset—hereafter simply referred to as “prophages” (Fig. S1b; Table S2). We chose this ORF number cutoff based on results of alignments and clustering where obviously truncated prophage sequences formed separate groups. Prophages found in 11 of 14 bacterial species, had, on average, the size of 38.8 kb [±1.2, 95% confidence interval (CI)], and coded for 53 (±1, 95% CI) proteins. Evolutionary relationships of prophages were studied at DNA and protein level to cover both close and more distant relations, respectively. We used the presence and position of conserved domains in phage genomes to identify phylotype-specific marker proteins. Multiple complementary analyses gave consistent results and created a framework for phage phylogeny.

### Phylogenetic diversity of prophages revealed by comparative genomics

**Fig. 1:**
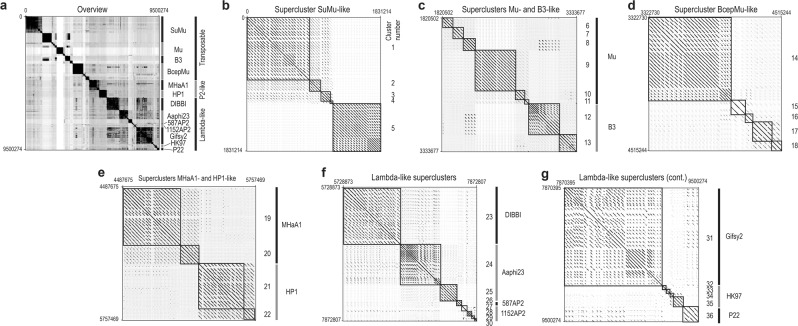
Diversity of prophages assessed by DNA similarity matrix analysis. **a** Overview dot plot of 243 prophage sequences arranged in groups, superclusters and clusters. Dot plot compared sorted and merged sequences of prophages (combined length of 9.5 megabase) on the *x*-axis, and the same collection of sequences on the *y*-axis of a plot. When the DNA residues of both sequences match at the same location on the plot, a dot is drawn at the corresponding position. Once the dots have been plotted, they will combine to form lines, and dense groups of lines will form black squares that correspond to clusters of similar genomes. The bigger the cluster, the higher the prevalence of prophages belonging to that cluster. The main diagonal represents the sequence’s alignment with itself; lines off the main diagonal but around it represent similar patterns within the closely related phage genomes. Signals found more far from the diagonal represents similar patterns within more distantly related phage genomes. Both halves of the graph created by the diagonal provide the identical information but we decided to keep them both for plot clarity. Enlarged sections for phages assigned to supercluster SuMu-like (**b**), Mu- and B3-like (**c**), BcepMu-like (**d**), MHaA1- and HP1-like (**e**), and lambda-like supercluster (**f,** **g**) are shown. Clusters (numbers 1–36) were highlighted with black frames. Superclusters and main groups are labeled and indicated by black or gray stripes. DNA coordinates for merged sequence are given in plots corners

Prophages were first stratified into groups based on two complementary approaches: (i) Genome sequences of prophages were aligned and clustered based on shared DNA content [28]. (ii) Predicted coding sequences from the phage genomes were used as a query against the Prophage/Virus Database (version from Aug 3, 2017) to identify the best matching phages [29]. Both approaches gave consistent groupings. Next, genome sequences of prophages were compared within stratified groups, i.e., individually for each supercluster, using the Genome BLAST Distance Phylogeny method via VICTOR [30], and manually curated. The relationship between genomes was visualized with a dot plot (Fig. 1). In total, 36 clusters of phage genomes were assigned to 13 superclusters and three main groups (Fig. 1a). Transposable phages, that are characterized by transpositional mode of replication [31], were divided into four superclusters (Fig. 1b–d). P2-like phages, recognized by the presence of a *Q-P-O-N-M-L* gene cluster, in which terminase genes (*P* and *M*) are located in reverse order and interspaced with scaffold- and capsid encoded genes [32], were distributed between two superclusters (Fig. 1e). Lambda-like phages, i.e., a diverse group of myo- and siphoviruses that somehow resemble phage lambda and have highly mosaic genomes [33, 34], were grouped into seven superclusters (Fig. 1f, g). Average nucleotide identity of sequences grouped in clusters ranged between 88.5 and 100%, whereas average Mash distances ranged between 0 and 0.082, as assessed by OrthoANI [35] and Mash [36], respectively (Table 1). There was a positive correlation between average Mash distances and (1-*ANI*), Pearson’s r = 0.83. Cluster sizes differed considerably and exchange of genome fragments between clusters was noticed. As phage mosaicism can pose a problem for whole-genome phylogenies, we looked for marker proteins that can be used to evaluate our classification.

**Table 1:** Characteristics of phage clusters

Supercluster	Cluster number	Prophages	Assemblies	OrthoANI (mean ± s.d.)	Mash distance (mean ± s.d.)	Predicted tail morphology	Taxonomy of lysogens	Most related phages	Reported phages	Comments	References
SuMu-like	1	20	0	93.9 ± 3.3	0.048 ± 0.02	Contractile	*H. aegyptius; H. haemolyticus; H. influenzae; H. parainfluenzae*	*Glaesserella* phage SuMu; *Mannheimia* phage 3927AP2			
	2	4	2	92.4 ± 3.7	0.05 ± 0.025	Contractile	*H. parainfluanzae*	*Glaesserella* phage SuMu; *Mannheimia* phage 3927AP2			
	3	3	0	100 ± 0.0	0.002 ± 0.002	Contractile	*H. influenzae*	*Glaesserella* phage SuMu; *Mannheimia* phage 3927AP2			
	4	1	1	–	–	Contractile	*H. parainfluenzae*	*Glaesserella* phage SuMu; *Mannheimia* phage 3927AP2			
	5	18	0	90.9 ± 0.1	0.01 ± 0.008	Flexible	*H. ducreyi*	*Glaesserella* phage SuMu; *Mannheimia* phage 3927AP2		DNA homology with phages from clusters 8 and 12	Gangaiah et al., [70]
	37	0	1	–	–	Flexible	*–*	*Glaesserella* phage SuMu; *Mannheimia* phage 3927AP2			Paez-Espino et al., [27]
Mu-like	6	3	0	95.1 ± 4.3	0.038 ± 0.033	Contractile	*H. haemolyticus; H. influenzae*	*Enterobacteria* phages Mu and SfMu; *Escherichia* phage D108			
	7	3	0	93.9 ± 3.3	0.05 ± 0.041	Contractile	*H. influenzae; H. parainfluenzae*	*Enterobacteria* phages Mu and SfMu; *Escherichia* phage D108	FluMu		Morgan et al., [21]
	8	4	0	100 ± 0.0	0.001 ± 0.001	Flexible	*H. ducreyi*	*Enterobacteria* phages Mu and SfMu; *Escherichia* phage D108		DNA homology with phages from clusters 5 and 12	Gangaiah et al., [70]
	9	13	0	99.9 ± 0.1	0.001 ± 0.001	Flexible	*H. ducreyi*	*Enterobacteria* phages Mu and SfMu; *Escherichia* phage D108			Gangaiah et al., [70]
	10	3	0	91.3 ± 6.7	0.067 ± 0.052	Flexible	*A. actinomycetemcomitans*	*Enterobacteria* phages Mu and SfMu; *Escherichia* phage D108			
	11	1	0	–	–	Flexible	*A. aphrophilus*	*Enterobacteria* phages Mu and SfMu; *Escherichia* phage D108			
B3-like	12	11	0	93.7 ± 6.1	0.058 ± 0.05	Flexible	*H. ducreyi*	*Pseudomonas* phages B3 and PM105		DNA homology with phages from clusters 5 and 8	Gangaiah et al., [70]
	13	6	0	90.0 ± 6.2	0.082 ± 0.051	Flexible	*A. actinomycetemcomitans; A*. sp. HMT-458	*Pseudomonas* phages B3 and PM105			
BcepMu-like	14	22	0	98.7 ± 1.7	0.021 ± 0.021	Contractile	*H. influenzae*	*Burkholderia* phages BcepMu and φE255			
	15	2	0	100	–	Contractile	*H. parainfluenzae*	*Burkholderia* phages BcepMu and φE255			
	16	2	0	88.5	–	Contractile	*A. aphrophilus/segnis*	*Burkholderia* phages BcepMu and φE255			
	17	4	2	99.2 ± 0.6	0.008 ± 0.005	Contractile	*H. influenzae*	*Burkholderia* phages BcepMu and φE255			
	18	2	0	100	–	Contractile	*H. paraphrohaemolyticus*	*Burkholderia* phages BcepMu and φE255			
MHaA1-like	19	16	5	93.7 ± 2.4	0.054 ± 0.02	Contractile	*H. aegyptius; H. haemolyticus; H. influenzae*	*Mannheimia* phages 587AP1 and φMHaA1			
	20	5	7	92.3 ± 2.8	0.054 ± 0.018	Contractile	*H. parainfluenzae*	*Mannheimia* phages 587AP1 and φMHaA1			
	38	0	2	–	–	Contractile	*–*	*Mannheimia* phages 587AP1 and φMHaA1			Paez-Espino et al., [27]
HP1-like	21	10	3	95.5 ± 2.5	0.043 ± 0.018	Contractile	*H. influenzae*	*Haemophilus* phages HP1 and HP2	HP1, HP2		Harm and Rupert, [19]; Williams et al., [20]
	22	3	16	90.9 ± 1.2	0.066 ± 0.019	Contractile	*H. parainfluenzae*	*Haemophilus* phages HP1 and HP2		One of phages has head morphogenesis protein resembling one from *Pasteurella* phage F108	
	39	0	3	–	–	Contractile	*–*	*Haemophilus* phages HP1 and HP2			Paez-Espino et al., [27]
	40	0	3	–	–	Contractile	*–*	*Haemophilus* phages HP1 and HP2			Paez-Espino et al., [27]
DIBBI-like	23	17	0	97.3 ± 1.3^$^	0.043 ± 0.017	–	*H. aegyptius; H. influenzae*	*Aggregatibacter* phages Aaphi23 and S1249		OrthoANI indicated three outliers, DNA homology with some phages from cluster 31	
Aaphi23-like	24	17	0	94.2 ± 3.5	0.07 ± 0.038	–	*H. aegyptius; H. haemolyticus; H. influenzae; H. parainfluenzae*	*Aggregatibacter* phages Aaphi23 and S1249			
	25	4	0	97.1 ± 1.6	0.029 ± 0.015	Contractile	*A. actinomycetemcomitans*	*Aggregatibacter* phages Aaphi23 and S1249	Aaphi23, S1249		Resch et al., [34]; Chen et al., [62]
	41	0	8	–	–		*–*	*Aggregatibacter* phages Aaphi23 and S1249		Two phages have similar head morphogenesis protein like phages from cluster 24	Paez-Espino et al., [27]
587AP2-like	26	2	1	99.9	0.000	–	*H. parainfluenzae*	*Mannheimia* phage 587AP2			
1152AP2-like	27	1	2	–	–	–	*H. parainfluenzae*	*Mannheimia* phage 1152AP2			
	28	3	0	95.8 ± 3.8	0.06 ± 0.051	Flexible	*H. paraphrohaemolyticus; H. parahaemolyticus*	*Mannheimia* phage 1152AP2			
	29	1	0	–	–	–	*H. haemolyticus*	*Mannheimia* phage 1152AP2			
	30	1	0	–	–	–	*H. haemolyticus*	*Mannheimia* phage 1152AP2			
Gifsy2-like	31	28	2	93.6 ± 4.7	0.057 ± 0.036	Flexible	*H. haemolyticus; H. influenzae; H. paraphrohaemolyticus; H. parainfluenzae*	Multiple phages		Some show DNA homology with phages from cluster 23	
	42	0	1	–	–	Flexible	*–*	*Mannheimia* phages 587AP2 and 535AP2			Paez-Espino et al., [27]
	43	0	5	–	–	Flexible	*–*	*Mannheimia* phages 587AP2 and 1152AP2			Paez-Espino et al., [27]
HK97-like	32	1	0	–	–	Flexible	*A. aphrophilus*	*Mannheimia* phage 1152AP2			
	33	1	1	–	–	Flexible?	*A. segnis*	*Mannheimia* phage 535AP2			
	34	1	0	–	–	Flexible	*H. parainfluenzae*	*Mannheimia* phage 1152AP2			
	35	3	18	93.8 ± 4.7	0.046 ± 0.003	Flexible	*H. parainfluenzae*	*Mannheimia* phage 587AP2			
	44	0	1	–	–	Flexible	*–*	*Pseudomonas* phages PA73 and SCH_Ab26			Paez-Espino et al., [27]
P22-like	36	3	0	–	0.024 ± 0.019	–	*H. influanzae*	*Mannheimia* phage 587AP2			
PA73-like	45	0	2	–	–	Flexible	*–*	*Mannheimia* phage 587AP2		Potentially defective	Paez-Espino et al., [27]
unknown 1	46	0	6	–	–	–	*–*	*Mannheimia* phage 587AP2		Potentially defective	Paez-Espino et al., [27]
phiKZ-like	47	0	4	–	–	–	–	*Pseudomonas* phage phiKZ		Genome size of 279 kbp, likely lytic, pseudolysogeny possible	Paez-Espino et al., [27]
	48	0	2	–	–	–	–	*Pseudomonas* phage phiKZ		Genome size of 241–254 kbp, likely lytic, pseudolysogeny possible	Paez-Espino et al., [27]
	49	0	2	–	–	–	–	*Pseudomonas* phage phiKZ		Genome size of 220 kbp, likely lytic, pseudolysogeny possible	Paez-Espino et al., [27]
	50	0	1	–	–	–	–	*Pseudomonas* phage phiKZ		Genome size of 211 kbp, likely lytic, pseudolysogeny possible	Paez-Espino et al., [27]
unknown 2	51	0	1	–	–	–	–	*Serratia* phage phiMAM13		Genome size of 190 kbp	Paez-Espino et al., [27]
S13-like	52	0	5	–	–	–	–	*Cronobacter* phage S13		Genome size of 151–152 kbp, likely lytic	Paez-Espino et al., [27]

### Phylogenetic diversity of prophages assessed using marker proteins

**Fig. 2:**
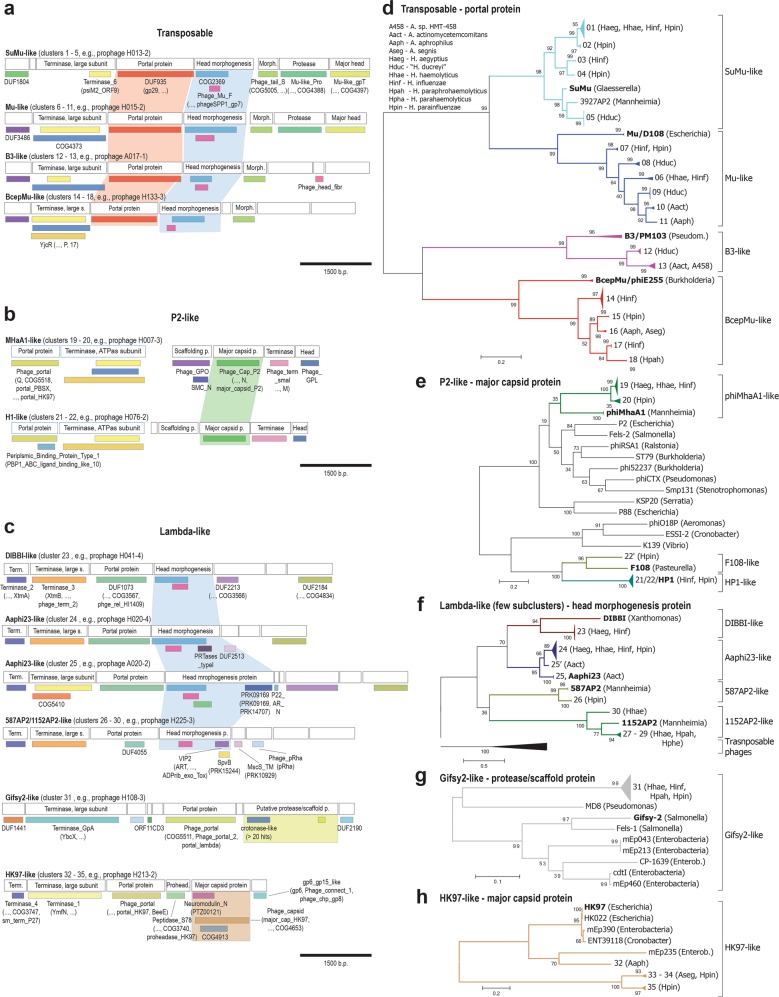
Diversity of prophages assessed by marker protein-based phylogeny. Genome segments coding for head morphogenesis and DNA packaging are shown for transposable (**a**), P2-like (**b**), and lambda-like (**c**) phages. Conserved domains are indicated and conserved marker proteins are highlighted. Blue frames in **b** indicate transcription in opposite direction (from right to left). The phylogenetic trees for transposable (**d**), P2-like (**e**), and lambda-like **(f, g, h)** phages were constructed. Reference sequences were included [32, 33, 99]. The evolutionary history was inferred by using the Maximum Likelihood method. The percentage of trees in which the associated taxa clustered together is shown next to the branches. The trees are drawn to scale, with branch lengths measured in the number of substitutions per site. Trees collapsed at cluster level. Number and length of studied sequences were as follows: 127 sequences with 324 amino acid positions (**d**), 55 with 300 aa (**e**), 63 with 131 aa (**f**), 37 with 554 aa (**g**), and 11 with 354 aa (**h**). Branches were colored to highlight superclusters. Abbreviated host taxonomy is given in brackets for each cluster. Full species names are listed in **d**

Phage genome sequences were screened for open reading frames. Genetics of these prophages will be described in detail elsewhere; here we screened the genomes for gene arrangements that were conserved for supercluster members and for marker proteins that could be used for phylogenetic profiling. We found the genome segment coding for proteins involved in head morphogenesis and DNA packaging (e.g., portal protein) to be the most suitable (Fig. 2a–c) because it is easy to identify and essential for the functionality of virions (i.e., entire phage particles). Our selection is in good agreement with previous comprehensive phylogenetic studies [31–33, 37]. Multiple markers were used because there is no single phage protein that has homologs in all phage genomes [38]. Next, we used the marker proteins to study the evolutionary relationship between newly reported and reference phages (Fig. 2d–h). Well-supported clusters and superclusters were observed. The findings were generally consistent with the results of the DNA-based full-genome analysis. One of the very rare exceptions was, for example, a recombination event where a HP1-like capsid gene (cluster 22) was replaced by one of F108-type (Fig. 2e). Such recombination events disrupt correlation between biomarker and cluster relationship (Table 1). To control for gene shuffling, we performed comparative analyses applying multiple biomarkers independently (data not shown). Occasionally, closely related clusters were grouped together (Fig. 2e, f, h), indicating that evolutionary relationship at fine phylogenetic level is better captured by analysis at whole-genome DNA level. We then compared our phylogenetic dataset with publicly available sequences of phages infecting *Aggregatibacter* and *Haemophilus* species [27, 39, 40] and discovered that only 10% of our phage clusters contain phage species that have been reported before (Table 1, for more details see Table S1).

### Diversity patterns of phages across species and subspecies clades

**Fig. 3:**
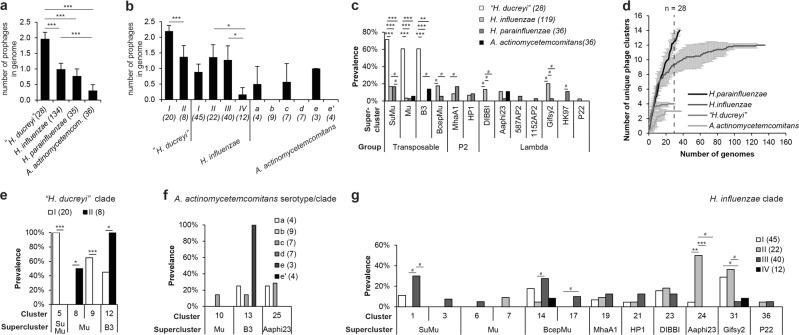
Prevalence and diversity patterns of phages across species and subspecies clades. **a** Number of prophages per genome plotted for four species that were best represented in genome database. **b** Number of prophages per genome plotted for subspecies clades of *“H. ducreyi”*, *H. influenzae*, and *A. actinomycetemcomitans*. Discrete population structure at subspecies resolution was inferred from whole-genome sequences [41–43]. **c** Prevalences of phage superclusters were plotted across four species. **d** Rarefaction curves were constructed to assess phage cluster richness from the results of sampling the genomes. Mean values are plotted and errors bars represent 95% CIs. The smallest sample size is indicated by a vertical dashed line. Prevalence of phage clusters across subspecies clades was plotted for “*H ducreyi”* (**e**), *A. actinomycetemcomitans* (**f**), and *H. influenzae* (**g**). Bar graph and error bars show mean value and 95 % CI, respectively. Number of observations per group is given in brackets. In **a** and **b**, significant differences between group means was detected by one-way ANOVA with post hoc Tukey test. In **c** and **e**–**g**, Fisher’s exact test (two-sided) was used to analyze the significance of the association between the phage presence of phage (grouped in superclusters or clusters) and host clades. Bonferroni correction was applied to compensate for multiple comparisons. The number of observations is given in brackets following the clade name. Symbols ***, **, *, and # indicated *p* < 0.001, *p* < 0.01, *p* < 0.05, and significant *p* value prior to Bonferroni correction, respectively

We uncovered the quantitative distribution of prophages across different host phylotypes. Because of the unequal distribution of available genomes, we focused on four well-represented species, i.e., *A. actinomycetemcomitans*, “*H. ducreyi”*, *H. influenzae*, and *H. parainfluenzae* as well as the subspecies clades of the first three species. Clades, i.e., discrete population structure at subspecies resolution (Table S2), were previously inferred from whole-genome sequence data [41–43]. Genomes of “*H. ducreyi”* harbor, on average, 2–6 times more prophages than other species (Fig. 3a). *H. influenzae* and *H. parainfluenzae* showed intermediate values. Differences in prophage prevalence were observed at subspecies resolution as well (Fig. 3b).

We then explored the qualitative distribution of phage groups, superclusters, and clusters across bacterial species and subspecies (Fig. 3c–g). Phages from all three main groups (i.e., transposable, P2-like, and lambda-like) lysogenized *H. influenzae* and *H. parainfluenzae* (Fig. 3c). *A. actinomycetemcomitans* was not lysogenized by P2-like phages, while “*H. ducreyi”* was exclusively lysogenized by transposable prophages. We observed interesting patterns in prophage distribution at supercluster level across bacterial species (Fig. 3c). The most striking observation was a very high prevalence of prophages from three transposable superclusters in genomes of “*H. ducreyi”*. The profiles for *H. influenzae* and *H. parainfluenzae* were generally similar, except for the distribution of DIBBI- and Gifsy2-like prophages, which were less prevalent in *H. parainfluenzae*. The Aaphi23-like supercluster was the only lambda-like supercluster represented in *A. actinomycetemcomitans* strains in contrast to *H. influenzae* and *H. parainfluenzae* strains that hosted prophages from multiple superclusters.

Subsequently, we looked at patterns of phage clusters and bacterial species at finer phylogenetic resolution. We first constructed rarefaction curves to assess phage richness from the results of sampling the genomes of selected species (Fig. 3d). This curve is a plot of the number of phage clusters as a function of the number of bacterial genomes studied. We decided to use genus-like clusters (as opposed to species-like viral operational taxonomic units, in short vOTUs) to effectively remove redundancy, although at a cost of diversity underestimation. Rarefaction curves generally grew rapidly at first, as the most common phage clusters were found, but plateaued (for all species but *H. parainfluenzae*) as only the rarest phage clusters remained to be sampled. Phage cluster richness (which we take as an approximate equivalent for phage genus richness) was high for *H. influenzae* and *H. parainfluenzae* and low for “*H. ducreyi”* and *A. actinomycetemcomitans. H. parainfluenzae* was lysogenized by the most taxonomically diverse group of phages, which was far from being fully explored in this dataset because of the limited number of available genome sequences.

Subspecies bacterial clades showing divergent traits are a sign of ongoing speciation. We wondered if, at such a fine phylogenetic resolution, we would be able to see additional patterns of phage diversity, which could shed light on a potential role of prophages in speciation processes. Indeed, we observed differences in the prevalences of phage clusters across such bacterial subspecies-like clades. Two clades of “*H. ducreyi”* were colonized by different transposable phage clusters (Fig. 3e). The effect was even more pronounced if truncated prophages were taken into account (Fig. S2). Clade-specific association was supported by balanced distribution of phage clusters across diseases and geographical origin (Fig. S2). Only half of *A. actinomycetemcomitans* serotypes/clades were lysogenized (Fig. 3f) but a high prevalence of cluster 13 prophages in serotype/clade e strains was noticed. *H. influenzae* clade II was associated with cluster 24-lysogeny (Fig. 3g). Strains from clade III were more often hosting transposable prophages from clusters 1, 14, and 17.

In summary, clear quantitative and qualitative differences in prophage profiles obtained for different clades suggest that either (i) phages piggybacked on hosts that outcompeted other variants in the process of natural selection, or (ii) phages were enriched due to either biological characteristics of their host clade (e.g., presence of specific receptor) and/or environmental factors shaping the occupied niche (e.g., favored specific phage variant that is able to co-exist with its host in a specific niche), or (iii) phages conferred a trait that is advantageous for their host, consequently contributing to their host’s success and, potentially, divergence, or (iv) a combination of factors favors the observed phage-host associations. Prophage patterns observed for *A. actinomycetemcomitans* strains could not be fully evaluated using the main dataset because of the small sample size. To address this problem, we included a second dataset built on a wide collection of clinical isolates.

### *Aggregatibacter* phages lysogenize strains of specific serotype/clade and show different host range

*A. actinomycetemcomitans* is an opportunistic pathogen that can devote most of its transcriptional effort to produce virulence factors, including adhesins and toxins specific for human neutrophils [44]. We explored prophage diversity patterns using strain collections [45–48] that encompass 157 isolates representing different serotypes, countries of origin, and clinical conditions (Table S3 and Fig. S3a). We developed PCR tests for detection of prophages in *A. actinomycetemcomitans* genomes (Fig. S3b, c). In *A. actinomycetemcomitans* strains, we detected 45 prophages from cluster 25, eight from cluster 13, and 11 from cluster 10 (Fig. S3d). No prophages were detected in genomes of related *Pasteurellaceae* species: *A. aphrophilus*, *A. segnis*, *H. influenzae*, *H. parahaemolyticus*, *H. parainfluenzae*, and *H. pittmaniae*.

**Fig. 4:**
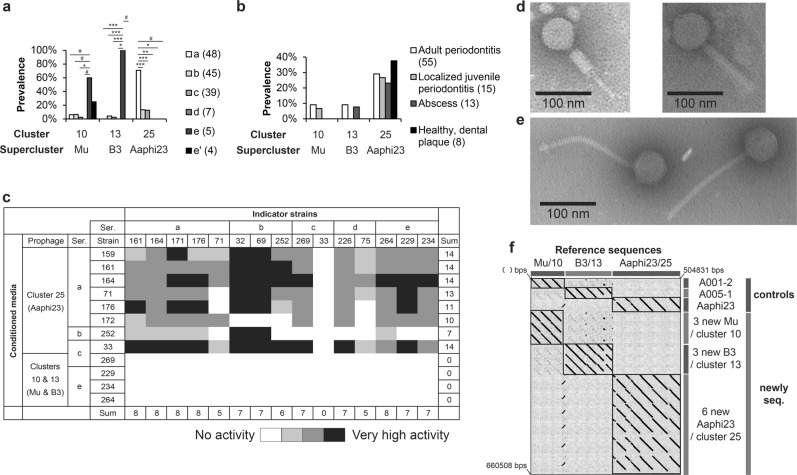
Characterization of *Aggregatibacter* phages. Prevalence of phages representing three clusters in different *A. actinomycetemcomitans* serotypes/clades (**a**) and clinical groups (**b**). Fisher’s exact test (two-sided) was used to analyze the significance of the association between the presence of phages and either host lineages or diseases. Bonferroni correction was applied to compensate for multiple comparisons. The number of observations is given in brackets following the clade name. Symbols ***, **, *, and # indicated *p* < 0.001, *p* < 0.01, *p* < 0.05, and significant *p* value prior to Bonferroni correction, respectively. **c** Antimicrobial activity of conditioned media from *A. actinomycetemcomitans* cultures treated with Mitomycin C. Results of drop spot assay are presented. Sources of conditioned media are listed in rows while the indicator strains are given in columns. Strains were grouped by prophage type and serotype/clade. Ser. is an abbreviation for serotype/clade. Electron micrographs of Aaphi23-like phage with a contractile tail and transposable phage with flexible tail induced from *A. actinomycetemcomitans* are shown in **d** and **e**, respectively. **f** Overview dot plot of newly sequenced genomes arranged in clusters. Dot plot compared sorted and merged sequences of reference prophages on the *x*-axis, and the control and new sequences on the *y*-axis of the plot. More details about the dot plot method can be found in legend of Fig. 1. Control and new sequences are indicated by black and gray stripes, respectively. DNA coordinates for merged sequence are given in the corners

We then looked at phage patterns across *Aggregatibacter* serotypes/clades (Fig. 4a). Cluster 25 prophages were highly prevalent in serotype/clade a strains. All five serotype/clade e strains were lysogenized with cluster 13 phages, and three of them were co-infected by cluster 10 phages (Fig. 4a). No lysogens could be detected among seven serotype/clade d strains. Next, we searched for phage patterns across clinical conditions. Cluster 25 prophages were highly prevalent in strains isolated from various conditions as well as from dental biofilms of healthy individuals (Fig. 4b). In contrast, transposable phages (i.e. from clusters 10 and 13) lysogenized only strains isolated from diseases such as periodontitis.

Subsequently, we explored the host range of *Aggregatibacter* phages. Prophages were induced from lysogens using Mitomycin C, and filtered conditioned media were tested for antimicrobial activity on *A. actinomycetemcomitans* indicator strains representing five serotypes (Fig. 4c). Conditioned media originating from strains harboring cluster 25 prophages in their genome showed broad antimicrobial activity. In contrast, samples from strains harboring transposable prophages from clusters 10 and 13 showed no activity. Super-sensitive indicator strains (SPS 32 and SPS 69) and super-resistant strain SPS 33 of *A. actinomycetemcomitans* were observed. Intriguingly, superinfection exclusion was observed in strains SPS 33 and SPS 71, but not fully functional in strains SPS 161 and SPS 164 and even absent in strain SPS 176. This may be a sign of genetic variation. Alternatively, lysis was caused indirectly, e.g., via bacteriocins or toxin induction. To address this problem, reference strains lysogenized with different phages have to be studied [49]. Virions characterized by contractile and flexible tails were detected in conditioned media from induced lysogens harboring cluster 25 and cluster 10/13 prophages, respectively (Fig. 4d, e). Full-genome sequencing performed for selected lysogens confirmed the presence of prophages (Fig. 4f; prophage sequences are provided in File S1) and identified mismatches in some of the PCR primers (Fig. S3e, f).

In summary, *Aggregatibacter* phages preferably lysogenize specific phylogenetic lineages [41, 46] and are not strongly correlated with specific clinical conditions. These phages show either an extremely narrow or a broad host range.

Phage patterns revealed across phylogenetic lineages made us wonder about their biogeographical distribution, i.e., niche tropism. We speculated that phages should undergo environmental selection and could, potentially, drive niche-association of their hosts. Therefore, we investigated the prevalence of *Aggregatibacter* and *Haemophilus* phages in human-associated microbiomes.

### *Aggregatibacter* and *Haemophilus* phages are prevalent in oral metagenomes and show niche specificity

The National Institutes of Health Human Microbiome Project (HMP) has provided one of the broadest characterizations of human-associated microbiomes to date [8, 26]. We used the HMP metagenome dataset to study the prevalence of *Aggregatibacter* and *Haemophilus* species and their temperate phages in different sites of the human body. The human oral cavity was a major reservoir of *Pasteurellaceae* species. *H. parainfluenzae* was highly abundant and prevalent (Fig. S4a, b). Few other species were prevalent, but usually at low abundance.

**Fig. 5:**
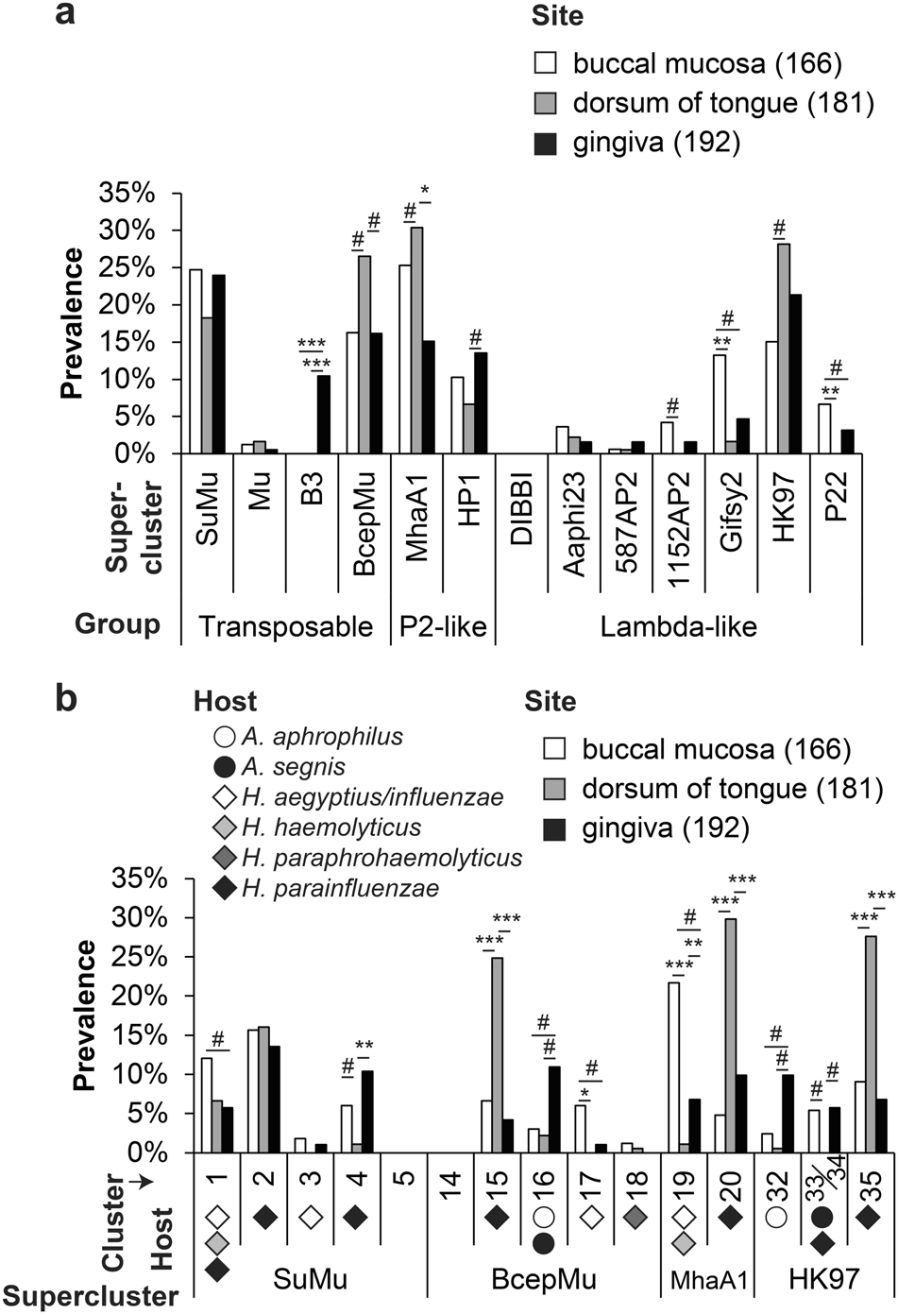
Prevalence of phage clusters among human microbiomes. Prevalence of phages grouped to superclusters (**a**) and clusters (**b**) at three oral sites, as assessed by metagenome analysis. Dataset from [26] was used. Predicted host of the phage is indicated in **b**. Fisher’s exact test (two-sided) was used to analyze the significance of the association between the phages and sites. Bonferroni correction was applied to compensate for multiple comparisons. The number of observations is given in brackets following the site name. Symbols ***, **, *, and # indicated *p* < 0.001, *p* < 0.01, *p* < 0.05, and significant *p* value prior to Bonferroni correction, respectively

By using marker protein sequences, the prevalence of phages (grouped to superclusters and clusters) was profiled at different sites of the human body. *Aggregatibacter* and *Haemophilus* phages were found exclusively in the oral cavity and pharynx. Data for the three best represented oral sites, i.e., buccal mucosa, gingiva, and tongue, is shown as, for other sites, only few samples were available (Fig. 5). Representatives of transposable superclusters SuMu and BcepMu were highly prevalent at all three sites (Fig. 5a), while Mu-like phages were rarely encountered. Among P2-like phages, both MhaA-like and HP1-like phages were quite prevalent. HK97-like phages were the most prevalent phages among lambda-like superclusters.

Subsequently, we studied the phages of the four most prevalent superclusters, i.e., SuMu, BcepMu, MHaA1, and HK97, at higher phylogenetic resolution (Fig. 5b). SuMu-like cluster 2 phages were uniformly prevalent at all sites. In contrast, the representatives of BcepMu-like clusters 15 showed strong preference for the microbiota of the tongue. Among MhaA1-like clusters (19 and 20) and HK97-like clusters [35], a clear preference for either buccal mucosa or tongue was observed. As expected, most of the detected phages belong to clusters whose members infect *H. parainfluenzae*, *H. haemolyticus*, commensal *Aggregatibacter* species or have broader host range (Fig. 5b).

In summary, we measured the high prevalence of SuMu-, BcepMu-, MhaA1-, and HK97-like phages in human oral metagenomes. Niche tropism was commonly observed for phages at higher phylogenetic resolution. A diverse group of phages infecting *H. parainfluenzae* was observed.

### Phage genomes assembled from metagenomes expanded the classification

The number and diversity of phage sequences extracted from metagenomes far exceeds that of phage isolates and could complement the prophage sequences retrieved from genomic databases [50]. High prevalence of phages observed in different oral niches prompted us to search the metagenomes for new sequences from *Aggregatibacter* and *Haemophilus* phages. IMG/VR is a database of cultured and uncultured DNA viruses and retroviruses that integrates the sequences with associated metadata, e.g., predicted host [27]. It contains over 760,000 viral genomes or genome fragments from: (i) publicly available isolate genomes, (ii) curated prophages, and, most important for us, (iii) ~8000 assembled metagenomes. We retrieved the metagenomic genome assemblies (version 2.0 from June 2018, accessed in February 2019) for all phages predicted to infect *Aggregatibacter* and/or *Haemophilus* species and classified them in the same way as the prophage sequences above.

**Fig. 6:**
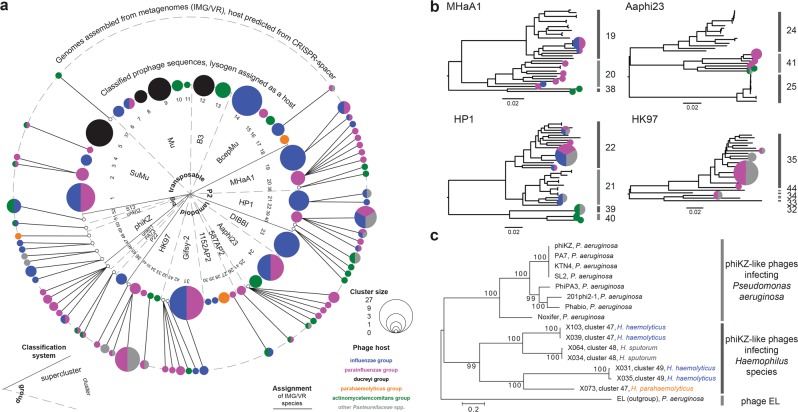
Classification of viral assemblies from metagenomes. **a** Comparison between prophage dataset and metagenome dataset. The following information is provided (starting from inner ring): phage group, phage supercluster, phage cluster, size of clusters and corresponding host species in prophage dataset, size of clusters and corresponding host species in metagenome dataset [27]. Assignment of IMG/VR assemblies to clusters is indicated by solid line linkers. **b** Dendrograms based on full-genome comparison for selected clusters constructed with VICTOR [30]. New sequences introduced by metagenomes are indicated by circle graphs [same as in **a**]. **c** The phylogenetic tree for phiKZ-like phages was constructed using sequences of major capsid protein. The evolutionary history was inferred by using the Maximum Likelihood method. The percentage of trees in which the associated taxa clustered together is shown next to the branches. The trees are drawn to scale, with branch lengths measured in the number of substitutions per site

In total, there were 115 IMG/VR singletons and 140 IMG/VR clusters encompassing 1090 viral contigs that were predicted to originate from phages infecting *Aggregatibacter* and *Haemophilus* species. Seven IMG/VR singletons and 53 IMG/VR clusters have at least one representative with a high quality draft genome (in total there were 108 such viral contigs). We assigned these high quality draft genomes to 14 superclusters and 28 clusters (Table 1, Table S1). Next, we compared the prophage dataset with the metagenome dataset (Fig. 6). The two approaches complemented each other: from a total of 52 clusters only 12 were shared, while 24 and 16 were unique for the prophage and metagenome datasets, respectively. Phages infecting important pathogens like *A. actinomycetemcomitans*, *H. influenzae*, and “*H. ducreyi*” were almost exclusively found in the prophage dataset (Fig. 6a, b), likely, because metagenomic studies poorly covered corresponding infections. Transposable phages were overrepresented in the prophage dataset (Fig. 6a), possibly, because metagenomic assemblies of transposable phages were less often categorized as high quality draft. On the other hand, the metagenome dataset was superior in providing sequences of phages infecting oral commensals. *Aggregatibacter* sp. HMT-458, *H. haemolyticus*, and *H. parainfluenzae* are poorly represented in the genome database but well covered by the HMP metagenomic study (Table S3). Finally, we found putative lytic phages characterized by big genome size (ranging from 151 to 279 kbp) exclusively in the metagenome dataset. Phages resembling *Pseudomonas* phage phiKZ (Fig. 6c) drew our special attention due to their potential therapeutic use [51].

In summary, metagenomic data complemented prophage screening. Addition of metagenomic assemblies to our classification system increased the number of phage genomes by 45%, created new clusters, and allowed cataloguing of putative lytic phages with therapeutic potential. Moreover, comparative analyses identified the bacterial species that are underrepresented in genome database and infectious diseases that have not been widely studied with metagenomics.

### Phage-host coevolution revealed by frequencies of DNA uptake signal sequences (USSs) and guanine–cytosine (GC) content

Uncovering the vast diversity of phages made us wonder if we could track phage-host coevolution. In this context, we came across a unique property of *Pasteurellaceae* species and phages, namely the presence of DNA uptake signal sequences (USSs) in their genomes.

*Pastuerallaceae* species exhibit strong preferences for genomic DNA from very close or somehow close relatives, a self-specificity likely arising from the combined effects of biases in the DNA uptake machinery and genomic overrepresentation of the USSs that this machinery prefers [52–54]. USSs have multiple “dialects” with two main clades within the *Pasteurellaceae* family preferring two distinct sequence variants (i.e., Hin-USS and Apl-USS, standing for *H*. *influenza*-like and *Actinobacillus pleuropneumoniae*-like, respectively) and having corresponding variants enriched in their genomes. Notably, in genome sequences of *H. influenzae* isolates, the uptake sequences have a higher density in core genes than in accessory genes [55]. This suggests that uptake sequences accumulate slowly, since recently acquired accessory genes would not yet have accumulated them [52]. Consequently, the frequency of USSs in a prophage may be a good proxy for phage-host coevolution time. This hypothesis is further supported by the existence of both USS-rich and USS-poor prophages/phage-like elements [56, 57].

**Fig. 7:**
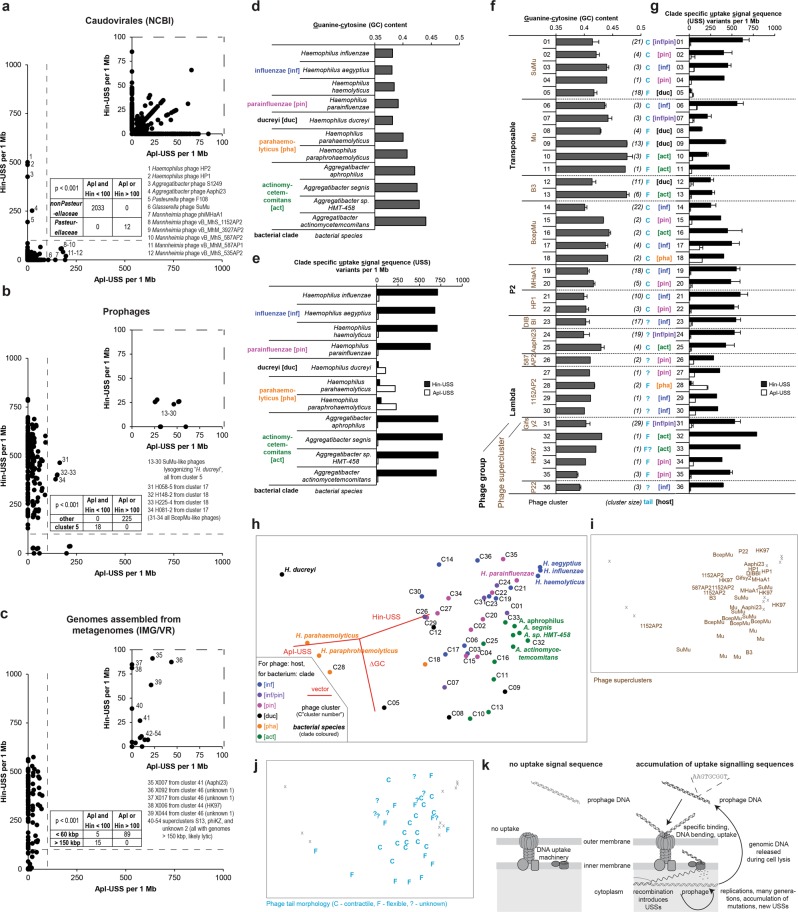
Frequencies of DNA uptake signal sequences (USSs) and guanine–cytosine content (GC%) in genome sequences of *Pasteurellaceae* species and their phages. Frequencies of clade-specific uptake signal sequence (USS) variants in both orientations and guanine–cytosine content (GC%) were measured for genomes from diverse phages and human-associated *Pasteurellaceae* species. Frequencies are given per 1 Mb (i.e., 10^6^ nt) of genome sequence. **a** Scatter plot showing frequencies of *H*. *influenzae* USS (Hin-USS) and *Actinobacillus pleuropneumoniae* USS (Apl-USS) in genomes of phages from order *Caudovirales* [25] on *y*- and *x*-axis, respectively. Enlarged fragment of plot covering low values is shown in top right corner. Contingency table summarizing studied groups is shown in the middle. Fisher’s exact test (two-sided) was used to analyze the significance of the association between the phage groups and presence of USSs at given cutoff. Phages characterized by noteworthy values are numbered and labeled. **b** like **a** but prophages classified in this study are presented. **c** like **a** but IMG/VR assemblies [27] are presented. **d** GC content was depicted for selected species grouped in clades based on genetic relationship inferred from concatenated nucleotide sequences (~2650 nt) of 16 S rRNA and three housekeeping (*infB*, *pgi*, *recA*) genes [1]. The subfamily clade labels were colored and abbreviated in square brackets throughout the figure. Genome sequences from either the representative National Center for Biotechnology Information (NCBI) strains or the type strains were retrieved from NCBI genome database. **e** Frequencies of Hin- and Apl-USS in the same genomes as in **d**. **f** GC content (mean ± s.d.) was depicted for phage clusters gathered in groups and superclusters (latter in brown throughout the figure). **g** Frequencies (mean ± s.d., per 1 Mb) of Hin-USS and Apl-USS in prophage sequences grouped like in **f**. For clusters depicted in **f** and **g** additional information is provided: the cluster size, which is the number of studied phages and written in parentheses, the predicted or confirmed morphology of phage tail written in light blue throughout the figure (C—contractile, F—flexible,?—unknown), and the abbreviated clade name for the phage host. **h** Ordination of bacterial species and phage clusters was constructed based on GC% and USS frequencies from **d** to **g**. The Bray-Curtis coefficient was calculated between every pair of samples using three variables: ΔGC (i.e., GC content reduced by the minimal GC in studied dataset), frequencies of Hin-USS, frequencies of Apl-USSs, each standardized by maximum (i.e., values were scaled so that their maxima across these three variables were always 100). Non-metric multidimensional scaling (nMDS) was used to represent the samples in two-dimensional space. Points were colored based on bacterial clades and phage host (labels starting with “C”). Superimposed is a vector plot for three variables (in red), with the vector direction for each variable reflecting the Pearson correlations of their values with the ordination axes, and length giving the multiple correlation coefficient from this linear regression on the ordination points. 2D-stress of 0.12 was observed. Same ordination was used in **i** and **j**. **i** Supercluster assignment was plotted for all phage clusters. Location of bacterial species is indicated by gray “x”. **j** Phage tail morphology for all phage clusters is given. **k** Mechanism of USS accumulation in prophages. Picture adapted from [52]

If the hypothesis holds true, phages known to lysogenize *Pasteurellaceae* species should have accumulated USSs in their genomes, all other phages not. Indeed, this is the case, as shown in Fig. 7a: USS-profiling of 2045 *Caudovirales* phage genomes [[39], accessed in February 2019)] correctly identified all the 12 phages lysogenizing *Pasteurellaceae* species (with a cutoff of either Hin- or Apl-USS frequency of 100, calculated per 1 megabase). Next, we profiled prophages identified in this study. As expected, almost all of them accumulated USSs (Fig. 7b). The only exception was the group of very closely related phages from cluster 5, suggesting that these phages were acquired by *Pasteurellaceae* more recently. Generally, Hin-dialect predominated, probably because the species using the Apl-dialect, e.g., *H. parahaemolyticus* (see below), were underrepresented in our study. Interestingly, four BcepMu-like phages (representing solely closely related clusters, either 17 or 18) seem to be “bilingual”, i.e., to accumulate both Hin- and Apl-USSs, which may suggest phage interaction with diverse hosts. Subsequently, we profiled high quality draft genomes [27] of *Aggregatibacter* and *Haemophilus* phages assembled from metagenomes (Fig. 7c). Again, almost all phages accumulated USSs and the Hin-dialect predominated. Among USS-poor phages we observed two distinct groups: (i) five phages located at boarder line mostly from supercluster Unknown 1, and (ii) all the phages with big (>150 kbp) genomes, all likely lytic.

To get better resolution of phage-host coevolution and to track host change, we coupled USS-profiling with guanine–cytosine content measure (GC%). Bacterial hosts (grouped to subfamily clades for the sake of simplicity) showed a broad range of GC% (Fig. 7d) and used either Hin- or Apl-dialects (Fig. 7e). It is unclear what the source of considerable variation of GC% among members of the same family, in the majority sharing the same habitat, is. Likewise, for phages grouped in clusters, we observed a variation in both GC% (Fig. 7f) and USS-profiles (Fig. 7g, for USS-profiles with single nucleotide mismatch see Fig. S5a). Generally, Hin-dialect predominated except for clusters 5, 17, and 18, as well as 28, where prophages lack any USS sequences, used both dialects, or favored the Apl-dialect, respectively (Fig. 7g). Frequencies of Hin-USS reached saturation at ~750, while maximal Apl-USS values were much smaller (Fig. 7e, g). To juxtapose phages with hosts, we created a 2D-ordination based on both USS-profiles and GC% (Fig. 7h, for 3D plot go to Fig. S5b). For phages, we observed a tendency to group with their corresponding hosts suggesting long-term coevolution. “*H. ducreyi*” phages were the noteworthy exception, and they likely originated either from *Aggregatibacter*-like hosts (cluster 9, high GC%, Hin-dialect) or potentially from species outside the *Pasteurellaceae* family (cluster 5, USS-poor). Traces of phage-host coevolution were also visible at phage supercluster level (Fig. 7d–g, i). Although phage clusters from some superclusters showed similar profiles, for most phage clusters (e.g., those grouped in Mu, BcepMu, HP1, and MHaA1 superclusters) clear shifts towards hosts were usually observed. For example, *Aggregatibacter* phages showed elevated GC%, or phages infecting the parahaemolyticus clade accumulated Apl-USS. In some cases, the cause of the shift remained unclear: e.g., why cluster 14 is very different from all the other BcepMu-like clusters, including cluster 17 that encompasses phages infecting the same host species, i.e., *H. influenzae*. Finally, we used tail morphologies as ordination labels (Fig. 7j), and observed that clusters containing phages with flexible tail showed more extreme profiles, when compared to clusters with myoviruses (characterized by contractile tail). This might suggest that certain types of phages are more prone to the observed shifts.

To sum up, USS frequencies in combination with GC% can be used to study phage-host coevolution if bacterial taxa (or their ancestors) exhibit specificity in DNA uptake. The longer a temperate phage co-evolves with its host, the likelier accumulation of USSs (Fig. 7k) and a GC% similar to the host’s is. An important unanswered question here is what exactly drives evolution of GC content in these phages.

## Discussion

Prophage diversity has been resolved in microbial genomes and metagenomes at coarse taxonomic resolutions to improve understanding of phage genetics, ecology, and evolution [11, 58–60]. Detailed analyses at finer resolution levels, i.e., for host genera or species, not only extended the understanding of phage biology but also provided a valuable reference for further explorations [16, 32, 33, 61]. Yet, comprehensive updating of the sequence resources for phages, from very biased and incomplete resources to one that is representative, ordered, characterized, and easily accessible has not been performed for most bacterial taxa. Advances in the understanding of prophage diversity are hampered by many factors: lack or unequal distribution of genomes/metagenomes for host clades/microbiomes, laborious processes of data acquisition and curation, rapidly changing taxonomy, lack of representative strain collections, absence of strain metadata, and simply the vast amount of understudied species to choose from. Here, we overcome these hurdles to study phages infecting members of the genera *Aggregatibacter* and *Haemophilus*, relevant human commensals and opportunistic pathogens. As phages infecting these species are largely unknown, we first aimed at revealing their taxonomic diversity. After establishing a phage classification scheme, we described the patterns of phage diversity across clades and niches and traced phage-host coevolution. Our findings advocate for more detailed studies exploring multiple ecological roles of these phages in the human microbiome.

Our study creates the first comprehensive classification scheme for *Aggregatibacter* and *Haemophilus* phages based on shared similarities in genome and marker-protein sequences (for comparison with existing classifications see Table S1). One of the most significant findings to emerge from prophage screening is that only three of our 33 phage clusters contain phage species previously reported in public databases, although many of these phages are highly prevalent in human oral microbiomes. In raw numbers, we analyzed 258 sequences of complete or almost complete prophages, five of which (Aaphi23, FluMu, HP1, HP2, and S1249) were described previously [19–21, 34, 62]. Classification of metagenomic assemblies yielded 107 additional sequences that form 16 new clusters, six of which likely contain lytic phages with therapeutic potential. Therefore, former microbiome studies focusing on oral phages likely suffered from incomplete reference datasets and propagation of erroneous annotations [63]. We hope that our phage reference catalogue can help others to overcome such and similar problems. We provide the genome and marker-protein sequences (Files S1 and S2) in a phylogenetic context (Table S2) that can be used to study *Aggregatibacter* and *Haemophilus* prophages at broad resolution as exemplified by analysis of the HMP dataset.

Another important issue for future research is to establish publicly available databases that provide easy access to well characterized and constantly updated sequences for oral phages. For example, data from recent work on the genomics of *Aggregatibacter* and *Haemophilus* species will broaden our catalogue [42, 43, 64]. *Aggregatibacter* and *Haemophilus* phages can also be found in metagenomics studies focused either on whole microbiomes or on viruses only (i.e., virome) [23, 65–67]. The latter are attractive since extracellular particles of phages are enriched and therefore low abundant phages can be detected and characterized [50]. On the other hand, analyzing datasets without enrichment has several advantages: it can detect lytic, temperate, and persistent infections, as well as overcome the biases arising from the enrichment procedure [50]. In all metagenomic studies the prediction of phage host(s) poses a challenge, but in silico estimations are often reliable and important guides for subsequent analyses, e.g., in vivo studies [50].

We refrained from assigning the full taxonomic affiliation to phages since the taxonomy of tailed phages is currently undergoing a major reorganization [68]. We are in a process of evaluating superclusters one by one, including sequencing additional phage genomes and screening for new members that infect hosts outside the genera *Aggregatibacter* and *Haemophilus*. We plan to gradually integrate our findings within current taxonomy.

Our results show that the quantitative and qualitative distribution of prophages across *Aggregatibacter* and *Haemophilus* species is not random. These patterns are signatures of co-evolutionary processes that maintain phage-host interaction. Very high prevalence (common presence) but low phylogenetic diversity (limited number of phage clusters) characterized prophages of the strict human pathogen “*H. ducreyi”*. These findings suggest either piggybacking where the host strain carrying these phages developed into a successful clone, or a mutualistic relationship, where transposable prophages confer a strong fitness benefit to “*H. ducreyi”* lysogens. Independent emergence of two similar prophage-rich lineages (clades I and II, see Fig. 3 and Fig. S2) put commensalism forward for consideration. There are several potential mechanisms that could drive such interactions between a pathogen and its prophages. It may be speculated that a prophage allows production of a factor that enhances adhesion, growth, or resistance of the host. Alternatively, specific disruption of a protein-coding gene by prophage integration may be advantageous during host’s adaptation [69]. Conserved locations of “*H. ducreyi”* prophages may suggest the latter scenario, as transposable prophage formation is believed to occur by integration into nearly random chromosomal locations [42, 70]. Another possible explanation is that these temperate bacteriophages act on the one hand as allelopathic agents, facilitating competition with other populations, and on the other hand as efficient host protectors against such an assault [16, 71]. In this scenario, phages that leak from a lysogenic population kill susceptible cells from a competitor population at a cost of small fraction of the lysogenic population but also protect its host from a secondary infection through superinfection exclusion. Positive frequency-dependent selection occurs because the more phage-releasing lysogens exist, the more beneficial it is to become a phage-resistant lysogen. Finally, the spontaneous or triggered phage-induced lysis of *“H. ducreyi”* strains may elicit a severe immune response due to release of endotoxins. Extracellular DNA produced in this process can be beneficial for the surviving subpopulation as an access to the key nutrients, source of DNA for genome repair or extension, or by conferring protection as a structural part of a biofilm matrix [72].

Mutualistic relationship implies that there are also benefits for the temperate phage to undergo the lysogenic cycle. One of the current paradigms for establishing lysogeny is low host abundance under stress or nutrient-limited conditions such that the phage is able to wait for more favorable host growth conditions (or host adaptation) in order to switch to lytic propagation. The alternative Piggyback-the-Winner model explains prevalence of lysogeny within nutrient rich environments (presumably such as mucous layers) by reduced phage predation control on bacterial abundance and superinfection exclusion, i.e., preventing closely related phages from infecting the same bacterial cell [73]. Additionally, if extracellular conditions may be physically detrimental to the phage virion (e.g., extreme pH), lysogeny would confer protection. Moreover, additional/alternative modes (i.e., as prophage) of dissemination, replication, and evolution may favor phage survival. Finally, lysogeny may enable broader host range by decreasing host-use trade-offs, e.g., by increasing total burst size calculated per single absorbed virion [74].

In contrast to “*H. ducreyi”* lysogens, *H. influenzae* and *H. parainfluenzae* strains are lysogenized by highly diverse populations of phages. A probable explanation is that these phages infect bacterial populations that are prevalent, abundant, active, biogeographically widespread, dynamic and diverse across multiple sites of the human mouth and pharynx [3–8, 75]. Diverse phage populations may favor host divergence, creating a positive feedback. Such coevolution can then maintain phenotypic and genetic diversity of both phages and their hosts [12]. Importantly, this process is driven by abiotic and other biotic components of the environment. The human mouth is a dynamic environment colonized by a complex and diverse microbiota, and the oral cavity is unique in providing distinct niches with physicochemical gradients, exposure to outer environment, and constant mixing by saliva flow [76, 77]. Consequently, both in situ coevolution of relevant adaptations, and colonization by phages that come in from sites in which these adaptations have already evolved, can take place. Combined, this provides an immense interaction space for diverse phages and host strains. In contrast to *Haemophilus* species, members of only three phage clusters infect strains of other oral species such as *A. actinomycetemcomitans*. However, this bacterial specialist is far less prevalent, low abundant (Fig. S4) and preferably inhabits the buccal epithelium and gingival sulcus [8, 78, 79].

Analysis of patterns at finer phylogenetic resolution of the bacterial hosts (i.e., at subspecies-like clades) gave further insights into the parasite-host ecology. Prophages varied remarkably between lineages, indicating either preserving forces or barriers in phage dissemination. Previously, a similar phenomenon was reported for siphoviruses (phages with flexible tail) that preferentially lysogenize *Staphylococcus aureus* strains from specific clonal complexes [80]. The authors suggested that spread of phages must be restricted potentially by restriction-modification systems and gave a number of examples how lysogeny can be preserved. Strikingly, in our study, similar patterns where shared by clades of different species, suggesting common ecoevolutionary driving forces.

As a first example, we noticed an extremely high prevalence of simultaneous infections with multiple transposable prophages in “*H. ducreyi”* strains from clades I and II but also in serotype/clade e strains of *A. actinomycetemcomitans*. These were co-infections of SuMu- Mu- and B3-like phages. Interestingly, in two clades of “*H. ducreyi”*, either Mu-like cluster 8 or Mu-like cluster 9 reached 100% prevalence when truncated prophages were included. On the other hand, B3-like phages where universally present in both clade II “*H. ducreyi”* strains and in serotype e strains of *A. actinomycetemcomitans*. All these bacterial strains originated from highly inflamed tissue—either genital or cutaneous ulcers or chronic gum disease. This would suggest a potential role of transposable phages in pathogenesis or in evasion of the immune response. The potential mechanisms were discussed in the previous section. Alternatively, we observed piggybacking, i.e., host strain carrying these phages developed into a highly infective clone by acquiring non-phage element(s) carrying advantageous gene(s). Conserved polylysogeny, the carriage of multiple prophages, additionally implies that interactions between prophages may be beneficial for a host, e.g., by reducing the rate of spontaneous lysis or regulation of gene expression.

The second pattern was a very high prevalence of Aaphi23-like prophages in serotype/clade a strains of *A. actinomycetemcomitans* and strains of *H. influenzae* grouped in clade II. This is in agreement with our previous findings for *A. actinomycetemcomitans* obtained by Southern blot hybridization [45]. In contrast to serotype/clade b and c strains, serotype/clade a strains of *A. actinomycetemcomitans* are usually genetically competent [81]. High prevalence of Aaphi23-like prophages in naturally competent linages suggests that DNA uptake may be a preserving force [52]. As high amounts of extracellular DNA can usually be found in oral biofilms, these prophages may protect competent lysogenic strains from harmful excessive uptake of foreign DNA by abortive killing induced via SOS response [82]. Phage mediated allopathy is another potential mechanism as we observed that Aaphi23-like phages showed broad activity spectrum and tended to have higher activity against serotype/clade b than a strains. A potential explanation is that the loss of competence in serotype/clade b and c strains was shown to be followed by the loss of Clustered Regularly Interspaced Short Palindromic Repeats (CRISPRs), bacterial adaptive immune systems that protect against parasitic DNA [83].

The third pattern was absence or very low incidence of prophages in serotype d strains of *A. actinomycetemcomitans* and clade IV strains of *H. influenzae*. This may be caused by a lack of efficient cell receptors or integration sites. Multiple phage resistance mechanisms may also form a barrier to lysogenization [14, 84]. We previously observed a high resistance of serotype d strains of *A. actinomycetemcomitans* to Aaphi23-like phages supporting this notion [45]. Alternatively, these strains thrive in particular microenvironments that might hinder phage dispersal or favor inactivation of the virions. Further experimental evaluation is required to identify the relevant explanation.

The spatial ecology of the microbial communities that inhabit the human body, in particular those of the mouth, deserves greater attention [76]. Important questions include which factors influence the size, boundary and spatial structure of microbial populations in a given niche. The influence of populations from different sites on each other is also not known. Here we profiled the prevalence of *Aggregatibacter* and *Haemophilus* phages in oral microbiomes and observed that members of related phage clusters have preferences for different oral niches. Putative clades of *H. parainfluenzae* showed niche adaptation as well [8]. Different niches have unique physical, chemical, and biological (i.e., microbiome) profiles that likely promote speciation in both phages and their bacterial hosts. Mucin, a heavily glycosylated protein, may play a key role in this, since *Pasteurellaceae* species preferentially colonize mucosal surfaces. It was reported that bacteria (including *Pasterurellaceae* species) and phages express receptors for specific mucins (e.g., outer membrane proteins or proteins containing Ig-like domains) [85–87]. We rarely observed Ig-fold in proteins from *Pasteurellaceae* phages (data not shown), so likely other protein domains contribute to mucin binding. On the one hand, immobilization and/or subdiffusive motion of phages and bacteria in mucosal layers increase the frequency of their encounters [88]. On the other hand, dispersion between niches, enabled by salivary and air flow, drags invaders. Phages may provide protection from such invading species [89]. Recently, spatially structured lytic to lysogenic switches were hypothesized to reconcile Piggyback-the-Winner and ‘Bacteriophage adherence to mucus’ models [90].

Discovery of high phage diversity prompted us to study the dynamics of phage-host coevolution. By measuring the frequencies of clade-specific DNA uptake signaling sequences (USSs) and guanine–cytosine content (GC%) we could trace phage-host coevolution and host shift events. We hope that future studies on the evolutionary ecology of prokaryotic immune mechanisms [91] and viral counter strategies [14, 15] reveal mechanisms behind observed dynamics and assign a proper time scale.

*Pasteurellaceae* species and phages provide a unique subject to study ecology and evolution. These bacteria and viruses are niche-specific and strongly host-associated, therefore barrier formation, dissemination, rate of speciation, and adaptive radiation, can be studied across different scales. They usually inhabit mucous layers that are easily accessible; for this reason, parameters like population size or host range can be obtained easier than for other habitats. Finally, *Pasteurellacae* species are usually genetically competent but some clades lost this property, consequently, the relationship between phage infection and DNA uptake can be dissected. Here we are providing a robust classification framework for phages that should be a good basis for such explorations.

## Conclusion

We established a classification scheme for *Aggregatibacter* and *Haemophilus* prophages and described the patterns of their diversity across clades and niches. Niche-specific coevolution may favor development of mutualistic relationships between members of specific bacterial clades and phage phylotypes. Further work is needed on the potential mechanistic underpinnings of prophage patterns and therapeutic use of lytic phages. In summary, *Aggregatibacter* and *Haemophilus* phages are prevalent, diverse, likely play a significant ecoevolutionary role in the human microbiome, and are therefore interesting candidates for further research.

## Experimental procedures

Information about used bacterial strains, culture conditions, species taxonomy, clades phylogeny, DNA isolation, Mitomycin C induction, drop spot assay, electron microscopy, genome sequencing, classification of metagenomic assemblies, USS-profiling, and statistical analyses can be found in Supplementary Information.

### Identification and characterization of prophages infecting *Aggregatibacter* and *Haemophilus* species

The PHASTER (PHAge Search Tool—Enhanced Release) web server [29] was used to identify and annotate prophage sequences within publicly available genomes of human-associated *Aggregatibacter* and *Haemophilus* spp. [1] retrieved from the NCBI genome database (March 2017). Defective and incomplete prophages are commonly encountered in bacterial genomes; therefore, we focused on non-duplicated, putatively intact prophages (PHASTER score >90) with genomes encoding a minimum of 40 ORFs (if not otherwise stated). We identified duplications by manual screening. Highly similar sequences were identified, metadata was retrieved when possible, and potential pseudoreplication (e.g., re-sequencing under different name, strains isolated from same clinical site or patient, colony variants of same isolate) was judged. Phage genome alignments were built using progressive Mauve [28]. Genome-based taxonomic classification was performed with VICTOR’s formula d_4_ [30]. Similarity matrix analysis was performed with Gepard using word length 10 [92]. Average nucleotide identity was calculated with OrthoANI [35]. Mash was used (k-mer size: 21; sketch-size: 1000) to estimate genome distance using MinHash [36]. Phage genome sequences were screened for open reading frames using Glimmer [93]. Translated sequences of proteins were annotated using three variants of BLAST against the following databases: (i) Prophage/Virus Database [29], (ii) Subsystem Technology [94], and (iii) Conserved Domain Database [95]. Multiple alignments of amino acid sequences were performed with MUSCLE [96]. Phylogenetic trees were constructed using Molecular Evolutionary Genetics Analysis v. 6 [97]. Evolutionary histories were inferred using the Maximum Likelihood method based on the Jones Taylor Thorton (JTT) matrix-based model [98]. The percentage of trees in which the associated taxa clustered together is shown next to the branches. Initial trees for the heuristic search were obtained by applying the Neighbor-Joining method to a matrix of pairwise distances estimated using a JTT model. The trees were drawn to scale, with branch lengths measuring the number of substitutions per site. Phage richness from the results of genome sampling was assessed by rarefaction (custom spreadsheet, 10 replicates).

### Identification of phages and their hosts in human-associated biofilm metagenomes

Metagenomic taxon abundances (MetaPhlAn2) were plotted for *Aggregatibacter* and *Haemophilus* species [8]. A species was regarded as present if its abundance was higher than 0.1%. To identify phages, metagenomic whole-genome assembly fasta files originating from the Human Microbiome Project WGS production phase I (HMP, study WGS-PP1) were downloaded via the HMP portal (https://portal.hmpdacc.org/) [26]. Files marked as “scaffolds” were removed from the present analysis, resulting in a database of 1021 contig files. Using a custom perl script, all contigs were combined into one file in which the fasta headers contained the unambiguous name of the original file in addition to the original contig identifier. All contigs were combined into one file, processed into a BLAST database, and the list of maker protein sequences (File S2) was searched against this database using tblastn with NCBI stand-alone BLAST (v. 2.5.0+). The BLAST E-value cutoff for this search was 1E-80. Based on the BLAST results table, an identity score was calculated as the percentage of query sequence covered by the BLAST hit, multiplied by the identity value of the BLAST hit. Only hits with an identity score of at least 95% were further analyzed. Prevalences of phage clusters and superclusters were plotted for different body sites.

### Detection of prophages by PCR

Used strains and primers are listed in Table S4 and Table S5, respectively. Detailed information can be found in Supplementary Information.

## Supplementary information

Supplementary material

File S1

File S2
