# Supplementary material for: Diversity patterns of bacteriophages infecting Aggregatibacter and Haemophilus species across clades and niches
Source: ISME J. 2019 Jun 14;13(10):2500–22. doi: 10.1038/s41396-019-0450-8 (PMC6776037; doi:10.1038/s41396-019-0450-8)
Supplement: Supplementary file 1 — Supplementary material [file 41396_2019_450_MOESM1_ESM.pdf]

Supplementary Information

Diversity patterns of bacteriophages infecting *Aggregatibacter* and *Haemophilus* species across clades and niches

Running title: Phages of *Aggregatibacter* and *Haemophilus* species

Szymon P. Szafrański [1, 2, 3 *], Mogens Kilian [4], Ines Yang [1, 2], Gesa Bei der Wieden [1, 2]; Andreas Winkel [1, 2], Jan Hegermann [5, 6], and Meike Stiesch [1, 2, 3]

[1] Department of Prosthetic Dentistry and Biomedical Materials Science, Hannover Medical School (MHH), Hannover, Germany

[2] Lower Saxony Centre for Biomedical Engineering, Implant Research and Development (NIFE), Hannover, Germany

[3] Cluster of Excellence RESIST (EXC 2155), Hannover, Germany

[4] Department of Biomedicine, Faculty of Health, Aarhus University, Aarhus, Denmark

[5] Research Core Unit Electron Microscopy, Hannover Medical School (MHH), Hannover, Germany

[6] Cluster of Excellence REBIRTH (EXC 62), Hannover, Germany.

[*] Correspondence to: Szymon P. Szafrański: Email: Szafranski.Szymon@mh-hannover.de

Competing Interest: The authors declare that they have no conflict of interest.

Supplementary materials and methods

Bacterial strains and cultures

The 157 *A. actinomycetemcomitans* strains and 32 strains of 7 related species examined in the study are listed in Tab. S4. These strains originate from multiple collections and cover different geographic origins, serotypes, and a range of diseases. Serotypes of strains that had not been previously characterized were assigned by PCR for (Tab. S5). The following solid media were used: for *A. actinomycetemcomitans* strains sTHBYA medium, i.e., Todd Hewitt Broth (Becton Dickinson, U.S.), 30 g/l, supplemented with yeast extract (Carl Roth, Germany), 5 g/l, heat inactivated horse serum (Sigma, Germany), 5 %, defibrinated sheep blood (Thermo Fisher Scientific, U.S), 5 %, and Agar-Agar, Kobe I (Carl Roth) 14 g/l; and for all other species Enriched Chocolate Agar Medium (Thermo Fisher Scientific). For liquid cultures, *A. actinomycetemcomitans* strains were cultivated in Todd Hewitt Broth with yeast extract (THBY). Incubation was performed at 37 °C in CO_2-enriched atmosphere (5 %).

Taxonomy and phylogeny of strains

Taxonomy of strains was assigned/confirmed either by blasting whole genome sequence or 16S rRNA gene amplicon sequences against eHOMD 16S rRNA RefSeq v. 15.1 (1). The generic affiliation of the *Pasteurellaceae* species has been constantly updated (2, 3). The most recent changes that we adopted are: renaming the causative agent of Glasser's disease in swine from "*Haemophilus parasuis*" to "*Glaesserella parasuis*" (after The National Center for Biotechnology Information (NCBI) taxonomy database) and recognizing unnamed human oral *Aggregatibacter* and *Haemophilus* species from eHOMD database (namely HMT numbers 36, 458, 851, and 908). *"H. ducreyi"* is not closely related to other *Haemophilus* species but is still assigned to genus *Haemophilus*. Species taxonomy of 12 strains from The NCBI genome

database was assigned and for 2 other strains was corrected: *H. influenzae* strains 156_HINF and 841_HINF were renamed to *H. parainfluenzae*. We were not able to unambiguously distinguish between "*Haemophilus* sp. HMT-36" and "*Haemophilus* sp. HMT-908" based on 16S rRNA gene sequences so such strains were reported as "*Haemophilus* sp. HMT-36/908". We also took into account the discrete population structure at subspecies resolution (Tab. S2) inferred from full genome sequences of *"H. ducreyi"* (4), *H. influenzae* (5), and *A. actinomycetemcomitans* (6).

DNA isolation and detection of prophages by PCR

DNA was isolated from pelleted overnight liquid cultures using the peqGOLD Bacterial DNA Mini Kit (VWR, U.S.). Genome sequences of *Aggregatibacter* prophages were aligned and conserved regions were identified. We developed PCR reactions for detection of three types of prophages in *A. actinomycetemcomitans* genomes by nine sets of primers (Tab. S5), three for each type, which were designed based on conserved regions in their genomes (Fig. S3b). Primers targeting regions R2 gave the best results for a test set and were applied for main screening (Fig. S3c). PCR-based screening was performed in triplicate on collections of *Aggregatibacter* and *Haemophilus* strains listed in Tab. S4. PCR products were analyzed by agarose gel electrophoresis. 1 % agarose gel pre-stained with peqGREEN dye [(VWR), 6 µl per 100 ml of gel] was loaded with PCR products and electrophoresis was performed in TAE buffer (Carl Roth) at 100 V for 1 h. Subsequently, gels were scanned in c150 imaging system (Azure Biosystems, US). Images were obtained with UV302 and exposure time of 10 sec. The PCR assay was regarded as positive if it reproducibly produced a strong band.

Mitomycin C induction and drop spot assays

Early log-phase broth cultures (absorbance at 600 nm ranging from 0.2 to 0.3) were treated with Mitomycin C (Carl Roth), 0.1 – 1 µg/ml, for 15 min. Subsequently old medium was replaced

with pre-warmed, fresh medium, and growth was monitored until lysis occurred or stationary phase was reached. Cells were pelleted (4,500 x g, 7 min) and conditioned media were sterile filtered through a 0.22 µm PVDF membrane (Carl Roth). Samples were stored at 4 °C. For drop spot assays, overnight cultures were diluted 32 times in semi-solid THBY medium containing 7 g/l agar and 1 mM $CaCl_2$ at 40 °C, and a lawn was poured (4 ml for Petri dish with diameter of 10 cm) on a thin layer of solid THBY medium (14 g/l agar). After drying for 15 min, 10 µl of sterile conditioned media (diluted if necessary) were spotted on lawns of indicator strains. Lysis zones or plaques were observed after 2 days of incubation.

Electron microscopy

Virions were sedimented from cooled sterile-filtered conditioned media by centrifugation for 90 min at 25,000 x g (F15-8x50cy rotor, Thermo Fisher Scientific). Supernatant was discarded, replaced by 0.1 M ammonium acetate, pH 7 solution, and centrifuged again (7). The procedure was repeated twice. Concentrated phages were adsorbed onto a carbon film for 30 – 120 sec, washed with molecular grade water, negatively stained with neutral 3% (w/v) phosphotungstic acid (pH 7.0, Sigma-Aldrich, Germany), attached to a copper specimen grid (400 mesh, Plano, Germany) and examined in a Morgagni transmission electron microscope (FEI, U.S.) at an acceleration voltage of 80 kV.

Genome sequencing

The following *A. actinomycetemcomitans* strains were studied: SPS 32, SPS 71, SPS 159, SPS 161, SPS 164, SPS 172, SPS 176, SPS 229, SPS 234, SPS 252, SPS 269, SPS 297, and SPS 312 (see Table S4). Genomic DNA (gDNA) was isolated from pelleted overnight liquid cultures using a phenol-chloroform-isoamyl alcohol (PCI) extraction. Bacterial cells were incubated for 1 - 4 h at 56°C in lysis buffer [sodium dodecyl sulfate (SDS), 0.6%; proteinase K, 0.12 mg/ml;

tris(hydroxymethyl)aminomethane (TRIS) , 10 mM, pH 8; ethylenediaminetetraacetic acid (EDTA), 1 mM]. Subsequently, 3 – 4 rounds of PCI extraction were performed, using equal volumes of water and organic phase, followed by a single round of chloroform extraction. Nucleic acids were precipitated with ethanol (overnight, at -80°C) and treated with RNase A (Thermo Fisher Scientific, Waltham, Massachusetts, US), 20 μg/ml at 37°C for 1 h. Removal of RNA was confirmed by agarose (0.7 %) electrophoresis. After RNA digestion, gDNA was purified by 3 - 4 rounds of PCI extraction followed by single round of chloroform extraction and ethanol precipitation. Concentration of gDNA in TRIS-EDTA buffer (1mM and 0.1 mM in water, respectively) was measured using Qubit 2.0 fluorometer (Thermo Fisher Scientific, Waltham, MA, US). Multiplexed Microbial SMRTbell Libraries were prepared according to manufacturer's instruction [see Procedure & Checklist – Preparing Multiplexed Microbial SMRTbell Libraries for the PacBio Sequel System; Part Number 101-489-000 Version 02 (May 2018)]. One and a half μg of gDNA were fragmented using g-TUBE devices (Covaris, Woburn, MA, US) and Minispin plus centrifuge (Eppendorf, Germany). Sheared gDNA was purified with AMPure PB beads. Based on Bioanalyzer output (2100 Bioanalyzer instrument, Agilent DNA 12000 Kit; both Agilent Technologies, Palo Alto, CA, US), DNA fragments had size of 10 kbp ± 1 kbp. Next, gDNA was treated with Exo VII, followed by DNA damage- and end- repair steps, and next round of AMPure PB beads purification. Subsequently, blunt barcoded adapters (custom made, similar to Barcoded Adapter Kit 8A and 8b) were ligated, samples were pooled (1 μg of each sample), followed by ligase inactivation, Exo III and VII treatment and three rounds of AMPure PB beads purification, primer annealing, and polymerase binding. Pool was sequenced on a PacBio Sequel instrument with following run conditions: 6 pM on plate loading; Sequel binding Kit 2.0; Sequel Sequencing Kit 2.1; movie time: 600 min; immobilization time: 120 min; PreExtension Time: 120 min. Using PacBio SMRT Link, sequencing reads were demultiplexed

and assembled using HGAP 4 with aggressive option on, genome length set to 2,000,000 and a seed coverage of 30 or 40. Contig number and coverage ranged from 1 to 23 and 49 – 177, respectively. The PHASTER (PHAge Search Tool – Enhanced Release) web server (8) was used to identify and annotate prophage sequences within sequenced genomes. Prophage sequences: A91-1, A92-1, A93-1, A94-1, A95-1, A96-1, A97-1, A97-3, A98-2, A98-5, A100-1, A100-3, can be found in File S1. Full genome sequences of lysogens will be published elsewhere.

Classification of metagenomic assemblies

Nucleotide sequences and metadata for IMG/VR metagenomic assemblies (9-12) were downloaded from https://genome.jgi.doe.gov/portal/IMG_VR/IMG_VR.home.html (IMG_VR/IMG_VR_2018-07-01_4/ IMG_VR: IMGVR_all_nucleotides.fna.gz and IMG_VR: IMGVR_all_Sequence_information.tsv). High-quality draft genomes of putative phages infecting *Aggregatibacter* and/or *Haemophilus* species were retrieved using pyfaidx (13) and analyzed similarly as prophage sequences (see M&M section in main manuscript).

USS-profiling

The following genome sequences were profiled: i) *Caudovirales* (14) (https://www.ncbi.nlm.nih.gov/genomes/GenomesGroup.cgi?taxid=28883, accessed in February 2019): ii) prophages identified in genomes of *Aggregatibacter* and *Haemophilus* species (this study, File S1) iii) IMG/VR metagenomic assemblies (9-12) - high-quality draft genomes of putative phages infecting *Aggregatibacter* and/or *Haemophilus* species (Tab. S3) and iv) genome sequences from either the representative National Center for Biotechnology Information (NCBI) strains or the following type strains: *Aggregatibacter* sp. HMT-458 W10330, *Aggregatibacter actinomycetemcomitans* 624, *Aggregatibacter aphrophilus* ATCC 33389, *Aggregatibacter segnis* ATCC 33393, *Haemophilus aegyptius* ATCC 11116, *Haemophilus ducreyi* 35000HP,

Haemophilus haemolyticus CCUG 12834, *Haemophilus influenzae* KW20, *Haemophilus parahaemolyticus* HK385, *Haemophilus parainfluenzae* ATCC 33392 *Haemophilus paraphrohaemolyticus* CCUG 3718 were retrieved from the NCBI genome database. A custom python script was used to count the frequencies of Hin-type USSs (AAGTGCGGT) and Apl-type USSs (ACAAGCGGT) on both DNA strands and to standardize them per 1 Mb of genome sequence (Fig. 7). Frequencies of singly mismatched USSs were also calculated (Fig. S5a). PRIMER & PERMANOVA+, (PRIMER-E, Plymouth, UK) were used to perform ordinations based on GC% and USS frequencies (15).

Statistical analyses

IBM SPSS Statistics (v. 24) was used for statistical analyses. One-way ANOVA (with post hoc Tukey Honestly Significant Difference test in case of more than two groups) was applied to judge the differences between group means of number of prophages in genome for different species or clades. Fisher's exact test (two-sided) was used to examine the significance of the association between the phage clusters and superclusters to clades, niches and presence of USSs. To compensate for multiple comparisons, we applied the Bonferroni correction. Numbers of observations are given in brackets following the class names.

Supplementary figures

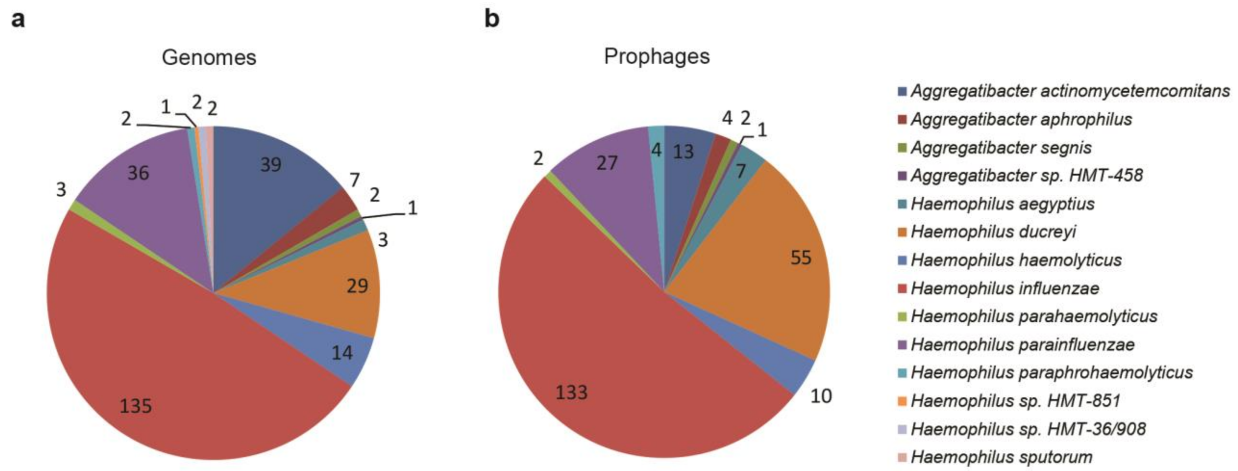

Fig. S1. Bacterial genomes and prophages.

Publically available genomes were screened for phage-like elements. **a)** Bacterial genomes screened for phage-like-elements. Taxonomy and number of studied strains is given. **b)** Prophages detected by VICTOR and predicted to be intact. Origin and number of prophages is reported.

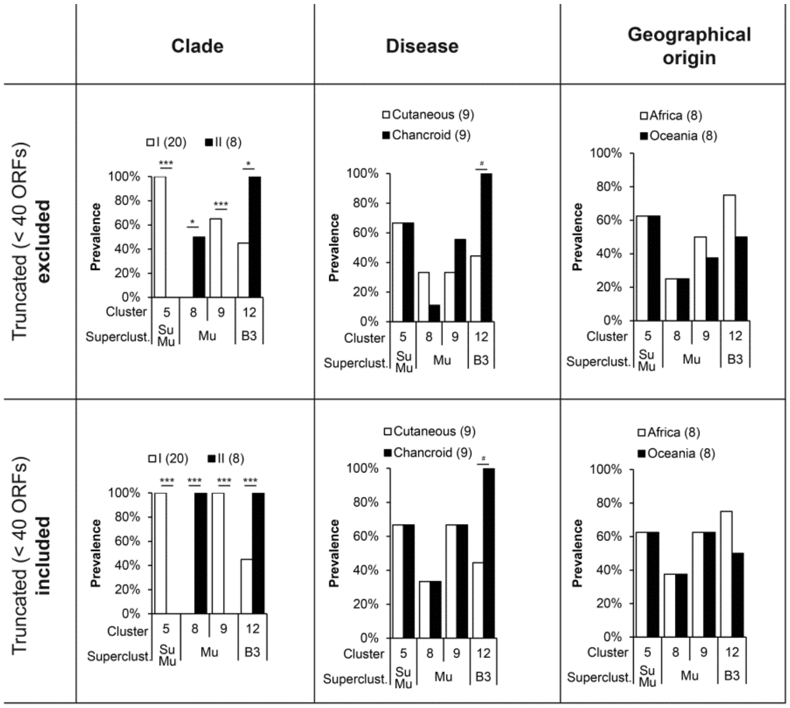

Fig. S2. Diversity patterns of phages for "*H. ducreyi*" clades, clinical origin and geographical location.

Prevalences of phage clusters were plotted across two subspecies-like clades (I and II), for two diseases (chancroid, a genital ulcerations in adults, and cutaneous ulcers in children) and for two continents (Africa and Oceania). Sequences and metadata were obtained from (16, 17). Disease and geographical groups were balanced for classes (and additionally for diseases by the latter). Predicted as intact but truncated (*i.e.*, with less than 40 predicted ORFs in genome fragment) prophages were either excluded (top) or included (bottom). Fisher's exact test (two-sided) was used to analyze the significance of the association between the phages and groups. Bonferroni correction was applied to compensate for multiple comparisons. The number of observations is given in brackets following the group name. Symbols ***, *, and # indicated $p < 0.001$, $p < 0.05$, and significant p value prior to Bonferroni correction, respectively.

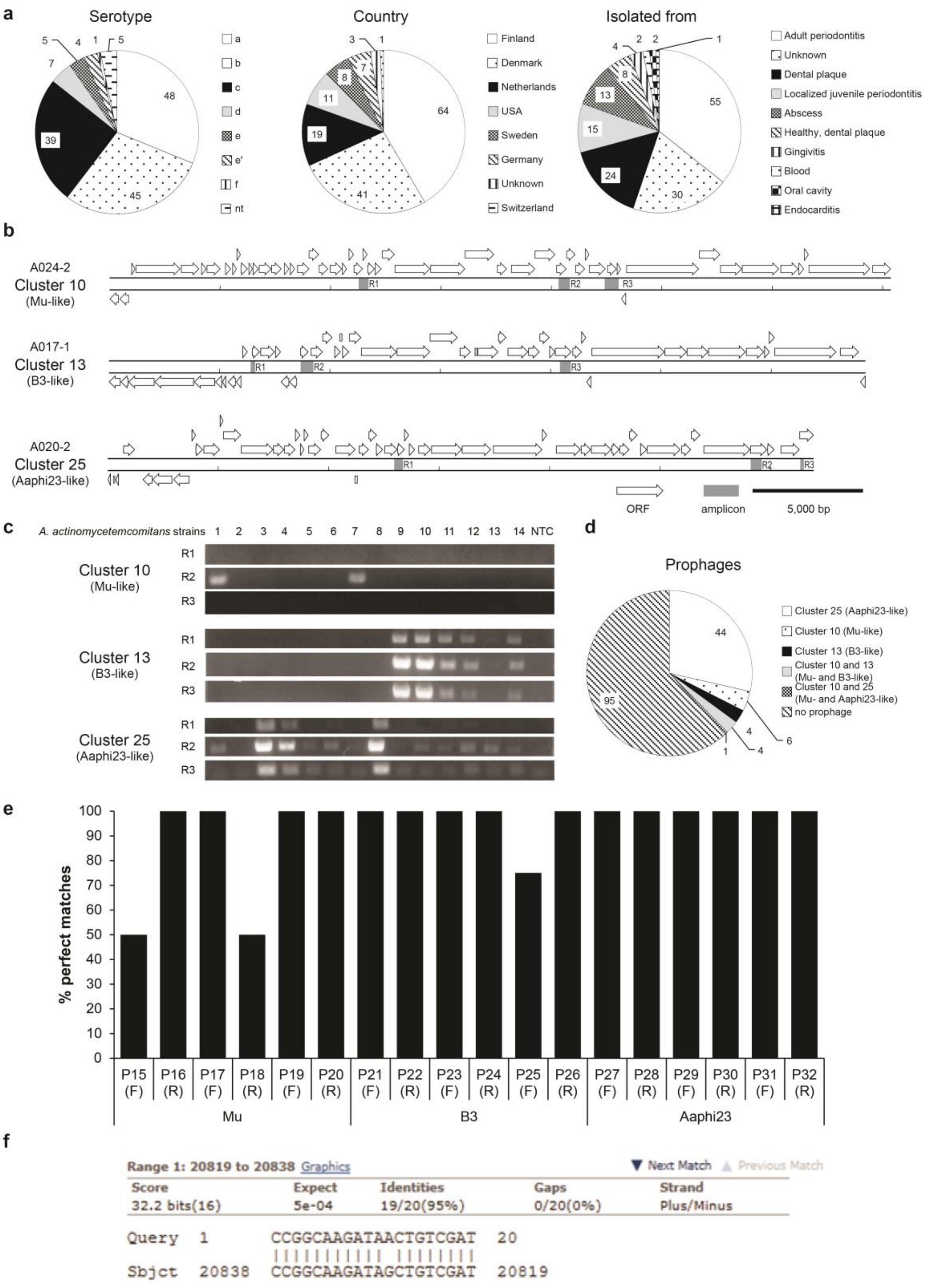

P18: CCGGCAAGATA**A**CTGCGAT could be replaced with degenerated P18new: CCGGCAAGATA**M**CTGTCGAT

Fig. S3. Detection of prophages in clinical isolates of *A. actincomycetemcomitans*.

Collection of *A. actinomycetemcomitans* strains were screened for prophages. **a)** Distributions of serotypes, countries of origin and source materials are plotted. **b)** PCR for detection of *Aggregatibacter* prophages from three clusters (10, 13, and 25). Nine sets of primers, three for each phage cluster, were designed based on conserved regions of their genomes. Amplicons are marked as gray boxes and numbered. **c)** Evaluation of primers. Fourteen strains were selected to compare the PCR tests targeting different conserve regions. **d**) Prophages detected in genomes of 154 *A. actinomycetemcomitans* strains. **e)** Re-evaluation of primers. Twelve newly sequenced prophages were included in BLASTn analysis. **f)** New degenerated primer may improve future screenings for Mu-like *Aggregatibacter* phages.

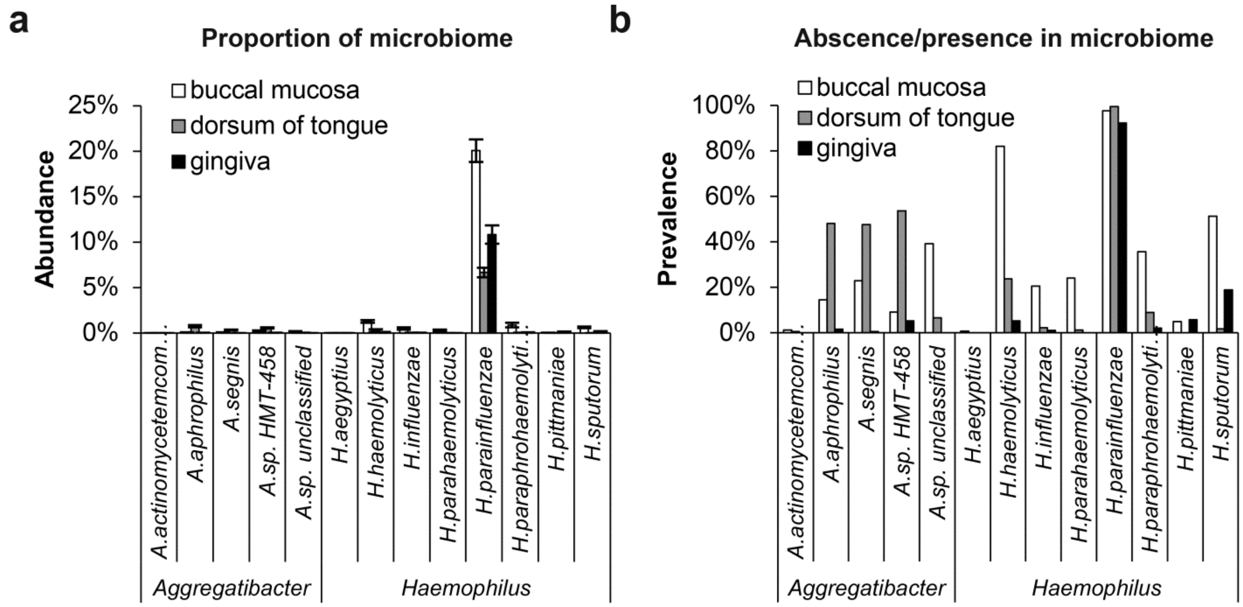

Fig. S4. Abundance and prevalence of human *Aggregatibacter* and *Haemophilus* species at three oral sites.

Metagenomes from the Human Microbiome Project (HMP) dataset (18). Mean relative abundance **(a)** and prevalence **(b)** of *Aggregatibacter* and *Haemophilus* species at three oral sites were assessed by metagenome analysis (18). Error bars represent SEM.

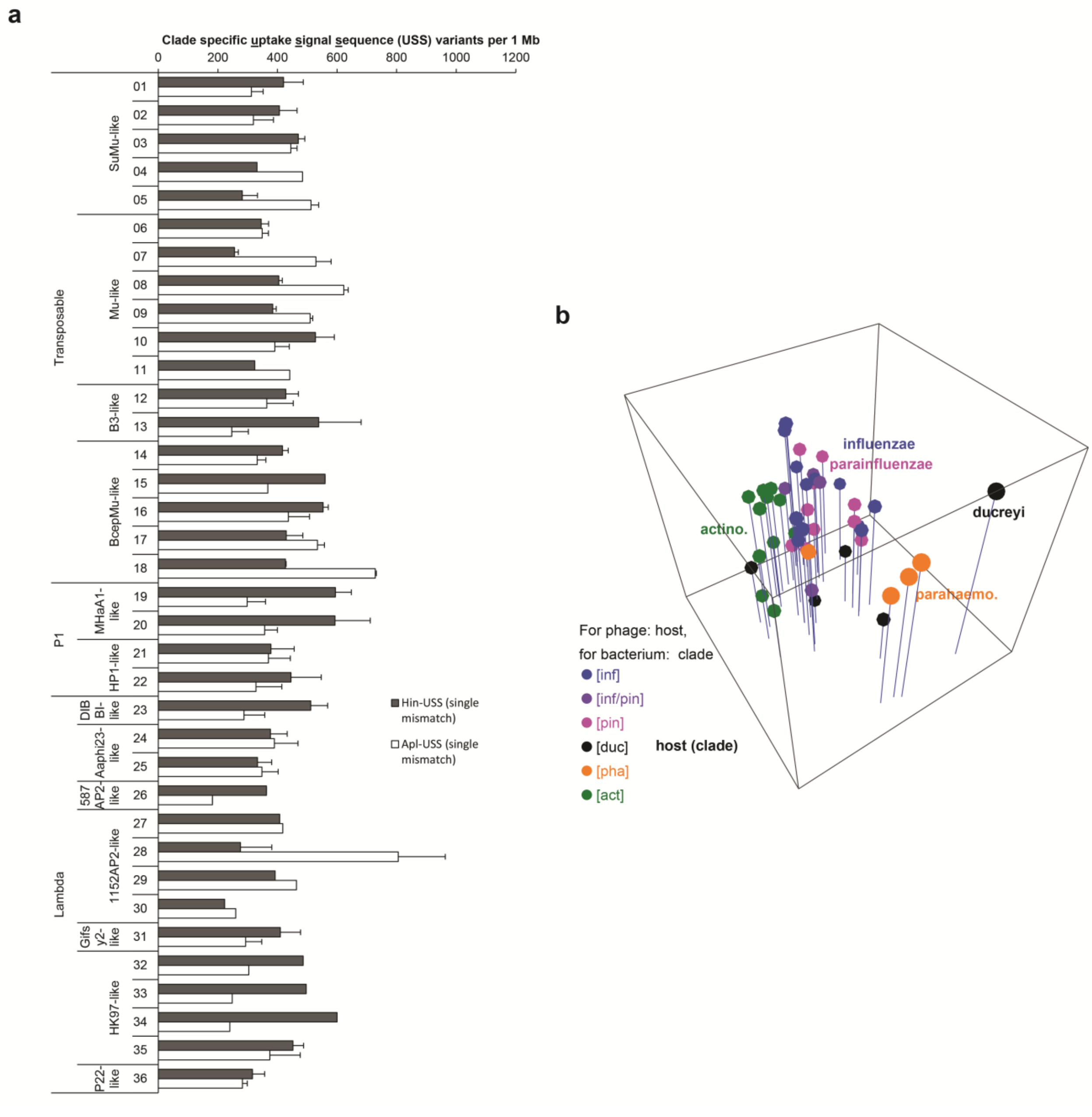

Fig. S5. Frequencies of DNA uptake signal sequences (USSs) and guanine-cytosine content (GC%) in genome sequences of *Pasteurellaceae* species and their phages

a) Frequencies (mean ± s.d., per 1 Mb) of single mismatched Hin-USS and Apl-USS in prophage sequences grouped in clusters. **b)** Ordination of bacterial species and phage clusters was constructed based on GC% and USS frequencies from. The Bray-Curtis coefficient was calculated between every pair of samples using three variables: ΔGC (i.e. GC content reduced by

the minimal GC in studied dataset), frequencies of Hin-USS, frequencies of Apl-USSs, each standardized by maximum (i.e. values were scaled so that their maxima across these three variables were always 100). Non-metric multidimensional scaling (nMDS) was used to represent the samples in three-dimensional space. Points were colored based on bacterial clades and phage host. 3D-stress of 0.06 was observed.

Supplementary tables

Classification system (compared features)

Group name (characteristics)

optional: subgroup(s)

List of (sub)group members (comment, our classification - evidence)

Baltimore Classification (how viral mRNA is produced)

Group I (double-stranded DNA viruses)

all known *Aggregatibacter* and *Haemophilus* phages

Bradley Classification (virion morphology, see **Figures 5d** and **5e**)

A (virion has a head with a hexagonal outline which may or may not be elongated; a tail with a contractile sheath is attached to it. This is usually rigid and may have various appendages, such as fibers or terminal structures)

Aggregatibacter phages phiAal7/phiAa, Aaphi23 and related Aaphi5, Aaphi76, Aaphi87, Aaphi92, Aaphi94, Aaphi97, Aaphi99, Aaphi247, AaphiST1, PAA24, PAA84, S1249, and other unnamed (all **cluster 25** – full DNA seq. or DNA fragment); *Haemophilus* phages HP1 and related HP2, S2A, S2B, and S2C (all **cluster 21** – full DNA seq. or DNA fragment), *Haemophilus* phage FluMu/Hin-Mu (**cluster 7** – full DNA seq.)

B (virion has also a six-sided head and a tail. Here, however, the tail is relatively flexible; it may or may not have terminal appendages; it is longer than the head diameter and has no contractile apparatus)

Aggregatibacter phages PAA17, PAA23, *Haemophilus* phage N3 (no or too little data to assign our classification)

C (virion also has a tail and a six-sided head, but the tail is shorter than the head diameter or maximal dimension, and may also have appendages attached to it, but, again, it is noncontractile)

unnamed *Aggregatibacter* phage (no or too little data to assign our classification)

unclassified (defective virion or virion morphology is not available)

Aggregatibacter phages Aabphi01 (phage related to **cluster 33** – 0.96 kb DNA fragment), PAA19, PAA22, (no or too little data to assign our classification)

Haemophilus phage phiflu (defective phage related to **cluster 24** - 18.2 kb DNA fragment)

National Center for Biotechnology Information (NCBI) Taxonomy (curated taxonomy, multiple features – variable between taxons)

Viruses; dsDNA viruses, no RNA stage; Caudovirales;

Myoviridae; Peduovirinae; Hp1virus; ***Haemophilus* virus HP1**

Haemophilus phage HP1c1 (**cluster 21** - full DNA seq.)

Myoviridae; Peduovirinae; Hp1virus; ***Haemophilus* virus HP2**

Haemophilus phage HP2 (**cluster 21** - full DNA seq.)

Myoviridae; **unclassified Myoviridae**

Aggregatibacter phage S1249 (**cluster 25** - full DNA seq.); *Haemophilus*[1] phage Aaphi23 (*Aggregatibacter* phage, **cluster 25** - full DNA seq.); *Haemophilus* phage S2A (**cluster 21** – 5.6 kb DNA fragment)

unclassified Caudovirales

Aggregatibacter phage Aabphi01 (phage related to **cluster 33** – 0.96 kb DNA fragment)

Virus Taxonomy, International Committee on Taxonomy of Viruses (ICTV) 2018a v1 (curated taxonomy, proteomic data and physicochemical parameters)

Caudovirales; Myoviridae; Peduovirinae; Hp1virus; ***Haemophilus* virus HP1**

Haemophilus phage HP1 (**cluster 21** - full DNA seq.)

Caudovirales; Myoviridae; Peduovirinae; Hp1virus; ***Haemophilus* virus HP2**

Haemophilus phage HP2 (**cluster 21** - full DNA seq.)

Integrated Microbial Genomes / Virus (IMG/VR) (catalog of metagenomic sequences, relies on curated set of viral protein families used as a bait to identify vial sequences directly from metagenomics assemblies, **see Figure 7**)

- Isolate viral genomes (iVGs) with host
  - *Aggregatibacter* phage S1249 (**cluster 25** - full DNA seq.), *Haemophilus* phage HP1 (**cluster 21** - full DNA seq.), *Haemophilus* phage HP2 (**cluster 21** - full DNA seq.)
- Metagenomic viral contigs (mVCs) with predicted host. Contigs are grouped into viral singletons (“sg_” prefix) and clusters (“vc_” or “vOTU_” prefix). In total there are 115 singletons and 140 clusters encompassing 1090 viral contigs (also known as viral counts). 7 singletons and 53 clusters have at least one representative with high quality draft genome (in total 108 viral contigs). These singletons and clusters were classified as below:
  - Caudovirales; Myoviridae; **Hp1virus**
    - sg_285414, sg_292341, sg_295118, vOTU_000492, vOTU_005379, vOTU_014390, vOTU_015434, vOTU_044378, vOTU_046698, vOTU_047192 (**clusters 21, 22, 39, and 40** - full DNA seq.)
  - Caudovirales; Myoviridae; **phiKZ_virus**
    - vOTU_002372, vOTU_004936, vOTU_007652, vOTU_009403, vOTU_011118, vOTU_019167, vOTU_086883 (**clusters 47 – 50** - full DNA seq.)
  - **unclassified**
    - 4 IMG/VR singletons and 39 IMG/VR clusters (**clusters 2, 4, 17, 19, 20, 26, 27, 31, 33, 35, 37, 38, 42, 43, 44, 45, 46, 51, and 52** - full DNA seq.)

Host (generic or species classification of phage host)

- By infectivity
  - Experimentally proved, e.g. based on plaque assay (**see Figure 5c**)
  - Predicted from bacterial CRISPR or by comparative phage genomics (putative, **see Figure 7a, Table S3**)
- By lysogeny
  - Confirmed by whole genome sequencing of a host (**see Figures 1-4, and 5f**)
  - Confirmed by prophage-specific PCR or Southern blot (**see Figure 5a**)
  - Predicted from nucleotide signatures, e.g. GC content or k-mer frequency, codon reflecting coevolution between phage and its host genome (putative, **see Figure 8**)

Niche (space where phage and its bacterial host co-exist, **see Figures 5b, 6, S2 and Table S3**)

- By environment
  - associated with living host
- By host’s host
  - human
- By anatomic location in host’s host
  - oral cavity
    - teeth/gingiva, tongue, buccal epithelium
  - respiratory track
    - pharynx
  - gastrointestinal tract
  - reproductive tract
  - integumentary system
  - infections in normally sterile sites
- By physiological state of host’s host
  - health, homeostasis
  - disease
    - opportunistic infection, dysbiosis

classical infection (host fulfills Koch's Postulates)

Cycle type (based on infection type)

Based on observed lysogeny, e.g. detected prophage or observed turbid plaques (delineated upfront)

temperate (can undergo either virion-productive or lysogenic cycle, virion production can occur during lytic or chronic cycle)

all[2] known *Aggregatibacter* and *Haemophilus* phages but phiKZ-like phages

Based on genetic information, e.g., presence of marker gene (e.g. coding for integrase or ParA/B)

temperate (can undergo either virion-productive or lysogenic cycle, virion production can occur during lytic or chronic cycle)

all[2] known *Aggregatibacter* and *Haemophilus* phages but phiKZ-like phages

virulent (displays only lytic cycle, i.e. no chronic or lysogenic cycle)

potentially phiKZ-like phages detected in metagenomes and predicted to infect *Haemophilus* spp. (please note that pseudolysogeny was reported for phiKZ-like phages infecting bacterial species from other familiy)

Table S1. Current classification systems for human-associated *Aggregatibacter* and *Haemophilus* species.

Links between our study and current classification systems are indicated. Baltimore Classification (19). Bradley Classification (7). NCBI Taxonomy (20). ICTV Virus Taxonomy (21, 22) IMG/VR database (9-11), also see (23). Host, see e.g. (10, 24). Niche. Cycle type (24, 25). [1] Erroneous host association, should be *Aggregatibacter* (26, 27). [2] Aabphi01 was previously erroneously described by us as virulent/strictly lytic (28).

TABLE S2

Strain ID	Region	Type	Supercluster	Cluster	Species	Strain	Clade	Refseq/Genbank	Region Length	Completeness	Score	# Total Proteins	Region Position	Most Common Phage	GC %
A001	1				*Aggregatibacter actinomycetemcomitans*	D11S-1	c	GCA_000146265.4_ASM14626v4	21.8	incomplete	30	10	1085788-1107597	Aggreg_S1249_NC_013597	41.96%
A001	2	*Transposable*	*Mu-like*	*10*	*Aggregatibacter actinomycetemcomitans*	D11S-1	c	GCA_000146265.4_ASM14626v4	30.6	intact	140	42	2075126-2105790	Escher_D108_NC_013594	48.19%
A001	3	*Lambda*	*Aaphi23-like*	*25*	*Aggregatibacter actinomycetemcomitans*	D11S-1	c	GCA_000146265.4_ASM14626v4	43.9	intact	150	63	1-43924	Haemop_Aaphi23_NC_004827	42.43%
A002	1				*Aggregatibacter actinomycetemcomitans*	H5P1	a	GCF_000226715.2_AaH5P-1v2.0	8.9	incomplete	30	8	57513-66416	Salisa_1_NC_017983	47.24%
A003	1				*Aggregatibacter actinomycetemcomitans*	I63B	d	GCF_000226735.2_AaI63Bv2.0	9.8	incomplete	40	13	152-10029	Bacill_AR9_NC_031039	44.07%
A003	2				*Aggregatibacter actinomycetemcomitans*	I63B	d	GCF_000226735.2_AaI63Bv2.0	9.2	incomplete	40	9	13754-23000	Salisa_1_NC_017983	47.10%
A004	1				*Aggregatibacter actinomycetemcomitans*	SC1083	e'	GCF_000226755.1_ASM22675v2	20.6	incomplete	40	8	109048-129689	Synech_S_CAM3_NC_031906	45.57%
A004	2				*Aggregatibacter actinomycetemcomitans*	SC1083	e'	GCF_000226755.1_ASM22675v2	37.8	incomplete	50	13	11261-49099	Erwini_vB_EamM_Phobos_NC_031043	41.64%
A005	1	*Transposable*	*B3-like*	*13*	*Aggregatibacter actinomycetemcomitans*	SCC393	e	GCF_000226775.2_AaSCC393v2.0	34.2	intact	150	44	49697-83921	Pseudo_vB_PaeS_PM105_NC_028667	47.08%
A006	1				*Aggregatibacter actinomycetemcomitans*	D18P1	f	GCF_000226795.2_AaD18P-1v2.0	7.4	incomplete	30	15	3-7501	Haemop_HP1_NC_001697	42.23%
A007	1				*Aggregatibacter actinomycetemcomitans*	SCC1398	b	GCF_000226815.2_AaSCC1398v2.0	33.7	incomplete	60	13	60015-93740	Entero_Tyrion_NC_031077	42.23%
A008	1				*Aggregatibacter actinomycetemcomitans*	I23C	b	GCF_000226835.2_AaI23Cv2.0	7.9	incomplete	40	10	29398-37320	Bacill_G_NC_023719	43.39%
A008	2				*Aggregatibacter actinomycetemcomitans*	I23C	b	GCF_000226835.2_AaI23Cv2.0	24.7	incomplete	30	20	152976-177689	Entero_Tyrion_NC_031077	42.97%
A009	1				*Aggregatibacter actinomycetemcomitans*	SCC2302	c	GCF_000226855.2_AaSCC2302v2.0	27.4	incomplete	30	12	104053-131537	Salmon_BP63_NC_031250	46.39%
A010	1				*Aggregatibacter actinomycetemcomitans*	D17P-3	a	GCF_000240985.2_AaD17P-3v2.0	26.8	questionable	80	38	23-26883	Escher_D108_NC_013594	47.44%
A010	2	*Transposable*	*B3-like*	*13*	*Aggregatibacter actinomycetemcomitans*	D17P-3	a	GCF_000240985.2_AaD17P-3v2.0	28.3	intact	100	43	11754-40114	Pseudo_vB_PaeS_PM105_NC_028667	48.06%
A011	1	*Transposable*	*B3-like*	*13*	*Aggregatibacter actinomycetemcomitans*	D17P-2	c	GCF_000241005.2_AaD17P-2v2.0	25.9	intact	120	41	1595-27572	Pseudo_vB_PaeS_PM105_NC_028667	47.66%
A011	2				*Aggregatibacter actinomycetemcomitans*	D17P-2	c	GCF_000241005.2_AaD17P-2v2.0	21.8	incomplete	30	10	135092-156901	Aggreg_S1249_NC_013597	41.96%
A011	3				*Aggregatibacter actinomycetemcomitans*	D17P-2	c	GCF_000241005.2_AaD17P-2v2.0	25.9	questionable	70	37	1503-27433	Escher_D108_NC_013594	46.16%
A012	1				*Aggregatibacter actinomycetemcomitans*	RhAA1	b	GCF_000259915.1_ASM25991v1	9.4	incomplete	30	13	357167-366650	Burkho_BcepMu_NC_005882	41.13%
A012	2	*Transposable*	*B3-like*	*13*	*Aggregatibacter actinomycetemcomitans*	RhAA1	b	GCF_000259915.1_ASM25991v1	35.4	intact	150	47	141-35639	Pseudo_B3_NC_006548	47.01%
A012	3				*Aggregatibacter actinomycetemcomitans*	RhAA1	b	GCF_000259915.1_ASM25991v1	8	incomplete	40	12	1496-9572	Bacill_G_NC_023719	40.03%
A012	4				*Aggregatibacter actinomycetemcomitans*	RhAA1	b	GCF_000259915.1_ASM25991v1	13.2	incomplete	20	19	2-13271	Aggreg_S1249_NC_013597	41.70%
A012	5	*Transposable*	*Mu-like*	*10*	*Aggregatibacter actinomycetemcomitans*	RhAA1	b	GCF_000259915.1_ASM25991v1	49.1	intact	150	69	385711-434899	Rhodov_vB_RhkS_P1_NC_031059	46.43%
A013	1				*Aggregatibacter actinomycetemcomitans*	ANH9381	b	GCF_000241025.1_ASM24102v2	7.9	incomplete	40	9	1161706-1169628	Bacill_G_NC_023719	43.42%
A013	2				*Aggregatibacter actinomycetemcomitans*	ANH9381	b	GCF_000241025.1_ASM24102v2	23.2	incomplete	30	18	1397560-1420823	Aggreg_S1249_NC_013597	42.61%
A014	1				*Aggregatibacter actinomycetemcomitans*	D7S-1	a	GCA_000163615.3_ASM16361v3	22.2	incomplete	50	11	1706890-1729163	Salisa_1_NC_017983	46.85%
A015	1				*Aggregatibacter actinomycetemcomitans*	Y4	b	GCF_000318155.1_ASM31815v1	17.1	incomplete	30	8	222-17384	Salisa_1_NC_017983	47.17%
A016	1				*Aggregatibacter actinomycetemcomitans*	AAS4A	c	GCF_000332895.2_AaAAS4av2.0	27.7	incomplete	30	12	88692-116393	Salmon_BP63_NC_031250	46.43%
A017	1	*Transposable*	*B3-like*	*13*	*Aggregatibacter actinomycetemcomitans*	A160	e	GCF_000332915.2_AaA160v2.0	34.2	intact	150	45	18332-52595	Pseudo_vB_PaeS_PM105_NC_028667	47.15%
A018	1				*Aggregatibacter actinomycetemcomitans*	SCC4092	b	GCF_000332935.2_AaSCC4092v2.0	33.7	incomplete	60	15	69962-103672	Entero_Tyrion_NC_031077	42.22%
A019	1				*Aggregatibacter actinomycetemcomitans*	S23A	b	GCF_000332955.2_AaS23Av2.0	23.2	incomplete	30	18	38034-61297	Aggreg_S1249_NC_013597	42.62%

TABLE S2

Strain ID	Region	Type	Supercluster	Cluster	Species	Strain	Clade	Refseq/Genbank	Region Length	Completeness	Score	# Total Proteins	Region Position	Most Common Phage	GC %
A020	1				*Aggregatibacter actinomycetemcomitans*	DSM 8324	c	GCF_000372365.1_ASM37236v1	14.3	incomplete	20	12	92160-106532	Haemop_Aaphi23_NC_004827	44.44%
A020	2	*Lambda*	*Aaphi23-like*	*25*	*Aggregatibacter actinomycetemcomitans*	DSM 8324	c	GCF_000372365.1_ASM37236v1	31.9	intact	109	45	72-32032	Haemop_Aaphi23_NC_004827	42.64%
A021	1				*Aggregatibacter actinomycetemcomitans*	HK1651	b	GCF_000604045.1_ASM60404v1	3.1	incomplete	10	7	1235036-1238230	Escher_D108_NC_013594	42.57%
A021	2				*Aggregatibacter actinomycetemcomitans*	HK1651	b	GCF_000604045.1_ASM60404v1	7.1	incomplete	20	13	1281165-1288347	Aggreg_S1249_NC_013597	39.22%
A021	3				*Aggregatibacter actinomycetemcomitans*	HK1651	b	GCF_000604045.1_ASM60404v1	7	incomplete	10	8	1839494-1846508	Entero_phi92_NC_023693	38.76%
A022	1				*Aggregatibacter actinomycetemcomitans*	RhAA1	b	GCF_001190115.1_ASM119011v1	9.4	incomplete	30	13	357167-366650	Burkho_BcepMu_NC_005882	41.13%
A022	2	*Transposable*	*B3-like*	*13*	*Aggregatibacter actinomycetemcomitans*	RhAA1	b	GCF_001190115.1_ASM119011v1	35.4	intact	150	47	141-35639	Pseudo_B3_NC_006548	47.01%
A022	3				*Aggregatibacter actinomycetemcomitans*	RhAA1	b	GCF_001190115.1_ASM119011v1	8	incomplete	40	12	1496-9572	Bacill_G_NC_023719	40.03%
A022	4				*Aggregatibacter actinomycetemcomitans*	RhAA1	b	GCF_001190115.1_ASM119011v1	13.2	incomplete	20	19	2-13271	Aggreg_S1249_NC_013597	41.70%
A022	5	*Transposable*	*Mu-like*	*10*	*Aggregatibacter actinomycetemcomitans*	RhAA1	b	GCF_001190115.1_ASM119011v1	49.1	intact	150	69	385712-434900	Rhodov_vB_RhkS_P1_NC_031059	46.43%
A023	1				*Aggregatibacter actinomycetemcomitans*	HK1651	b	GCF_001191585.1_ASM119158v1	3.1	incomplete	10	7	1234981-1238175	Escher_D108_NC_013594	42.63%
A023	2				*Aggregatibacter actinomycetemcomitans*	HK1651	b	GCF_001191585.1_ASM119158v1	7.1	incomplete	20	13	1281114-1288299	Aggreg_S1249_NC_013597	39.26%
A023	3				*Aggregatibacter actinomycetemcomitans*	HK1651	b	GCF_001191585.1_ASM119158v1	7	incomplete	10	8	1839429-1846443	Entero_phi92_NC_023693	38.80%
A024	1	*Lambda*	*Aaphi23-like*	*25*	*Aggregatibacter actinomycetemcomitans*	NUM4039	g	GCF_001547775.1_ASM154777v1	48.3	intact	120	71	1113480-1161870	Haemop_Aaphi23_NC_004827	43.12%
A024	2	*Transposable*	*Mu-like*	*10*	*Aggregatibacter actinomycetemcomitans*	NUM4039	g	GCF_001547775.1_ASM154777v1	35.3	intact	140	48	1848707-1884075	Escher_D108_NC_013594	48.01%
A025	1				*Aggregatibacter actinomycetemcomitans*	VT1169	b	GCF_001594245.1_ASM159424v1	7.9	incomplete	40	9	322261-330183	Bacill_G_NC_023719	43.38%
A025	2				*Aggregatibacter actinomycetemcomitans*	VT1169	b	GCF_001594245.1_ASM159424v1	22.9	incomplete	30	16	558331-581288	Haemop_Aaphi23_NC_004827	42.75%
A026	1	*Lambda*	*Aaphi23-like*	*25*	*Aggregatibacter actinomycetemcomitans*	624	a	GCF_001594265.1_ASM159426v1	49.5	intact	129	67	1806040-1855588	Haemop_Aaphi23_NC_004827	42.96%
A027	-				*Aggregatibacter actinomycetemcomitans*	SA3733	d	GCA_001596395.1_SA3733v1.0	-	no prophage	-	-	-	-	-
A028	1				*Aggregatibacter actinomycetemcomitans*	SA2149	c	GCF_001596225.1_SA2149v1.0	21.8	incomplete	30	10	50673-72482	Aggreg_S1249_NC_013597	41.93%
A029	1				*Aggregatibacter actinomycetemcomitans*	SC936	e'	GCF_001596235.1_SC936v1.0	37.6	incomplete	60	26	437-38094	Gordon_Yeezy_NC_031269	42.17%
A029	2				*Aggregatibacter actinomycetemcomitans*	SC936	e'	GCF_001596235.1_SC936v1.0	33.2	incomplete	50	22	16562-49853	Haemop_Aaphi23_NC_004827	43.94%
A030	1	*Transposable*	*B3-like*	*13*	*Aggregatibacter actinomycetemcomitans*	SA2876	e	GCF_001596245.1_SA2876v1.0	34.2	intact	150	45	49707-83970	Pseudo_vB_PaeS_PM105_NC_028667	47.15%
A031	1				*Aggregatibacter actinomycetemcomitans*	SA508	d	GCF_001596255.1_SA508v1.0	8.9	incomplete	30	8	2080-10983	Salisa_1_NC_017983	47.25%
A032	1				*Aggregatibacter actinomycetemcomitans*	SC29R	f	GCF_001596305.1_SC29Rv1.0	9.2	incomplete	30	9	1-9230	Salisa_1_NC_017983	47.07%
A032	2				*Aggregatibacter actinomycetemcomitans*	SC29R	f	GCF_001596305.1_SC29Rv1.0	7	incomplete	20	15	63-7157	Haemop_HP1_NC_001697	42.44%
A032	3				*Aggregatibacter actinomycetemcomitans*	SC29R	f	GCF_001596305.1_SC29Rv1.0	7	incomplete	10	8	1305-8393	Entero_phi92_NC_023693	39.51%
A033	-				*Aggregatibacter actinomycetemcomitans*	ANH9776	e'	GCF_001596315.1_ANH9776v1.0	-	no prophage	-	-	-	-	-
A034	-				*Aggregatibacter actinomycetemcomitans*	SA2200	d	GCF_001596325.1_SA2200v1.0	-	no prophage	-	-	-	-	-
A035	1				*Aggregatibacter actinomycetemcomitans*	SA269	d	GCF_001596335.1_SA269v1.0	8.9	incomplete	30	8	12037-20940	Salisa_1_NC_017983	47.26%
A036	1				*Aggregatibacter actinomycetemcomitans*	SA3033	d	GCF_001596385.1_SA3033v1.0	9.8	incomplete	40	12	17209-27086	Erwini_vB_EamP_Frozen_NC_031062	44.07%
A036	2				*Aggregatibacter actinomycetemcomitans*	SA3033	d	GCF_001596385.1_SA3033v1.0	9.2	incomplete	40	9	7-9253	Salisa_1_NC_017983	47.10%

TABLE S2

Strain ID	Region	Type	Supercluster	Cluster	Species	Strain	Clade	Refseq/Genbank	Region Length	Completeness	Score	# Total Proteins	Region Position	Most Common Phage	GC %
A037	1				*Aggregatibacter actinomycetemcomitans*	SC383s	c	GCF_001596415.1_SC383sv1.0	21.8	incomplete	30	10	50579-72388	Aggreg_S1249_NC_013597	41.93%
A038	1				*Aggregatibacter actinomycetemcomitans*	SA3096	e'	GCF_001596425.1_SA3096v1.0	13.5	incomplete	40	8	14035-27540	Entero_P4_NC_001609	45.31%
A039	1				*Aggregatibacter actinomycetemcomitans*	IDH781	d	GCF_001690155.1_ASM169015v1	21.2	incomplete	60	12	115372-136643	Salisa_1_NC_017983	45.95%
A039	2				*Aggregatibacter actinomycetemcomitans*	IDH781	d	GCF_001690155.1_ASM169015v1	4.7	incomplete	40	7	770709-775499	Sulfol_virus_NC_003214	44.29%
A039	3				*Aggregatibacter actinomycetemcomitans*	IDH781	d	GCF_001690155.1_ASM169015v1	9.8	incomplete	40	14	794973-804850	Erwini_vB_EamP_Frozen_NC_031062	44.06%
A039	4				*Aggregatibacter actinomycetemcomitans*	IDH781	d	GCF_001690155.1_ASM169015v1	6.8	incomplete	40	10	1849700-1856500	Bacill_G_NC_023719	42.96%
A040	1	*Lambda*	*HK97-like*	*32*		NJ8700		GCF_000022985.1_ASM2298v1	49.3	intact	150	61	462224-511613	Mannhe_vB_MhS_1152AP2_NC_028956	43.07%
A040	2					NJ8700		GCF_000022985.1_ASM2298v1	23.9	incomplete	60	20	2249878-2273824	Burkho_BcepMu_NC_005882	41.39%
A041	1	*Lambda*	*HK97-like*	*33*		ATCC 33393		GCF_000185305.1_ASM18530v1	40.3	intact	150	45	1197412-1237713	Mannhe_vB_MhS_587AP2_NC_028743	42.05%
A041	2					ATCC 33393		GCF_000185305.1_ASM18530v1	29.3	questionable	90	28	1573398-1602743	Salmon_ST64B_NC_004313	41.49%
A042	1					ATCC 33389		GCF_000226495.2_ASM22649v3	23.9	incomplete	50	19	31094-55065	Burkho_BcepMu_NC_005882	41.18%
A042	2	*Transposable*	*Mu-like*	*11*		ATCC 33389		GCF_000226495.2_ASM22649v3	33.9	intact	140	45	241-34223	Escher_D108_NC_013594	47.23%
A043	1					F0387		GCF_000231255.1_Aggr_aphr_F0387_V1	32.2	questionable	70	22	327999-360217	Burkho_BcepMu_NC_005882	42.34%
A044	1	*Transposable*	*B3-like*	*13*	*Aggregatibacter* sp. HMT-458	W10330		GCF_000466335.1_ASM46633v1	36.6	intact	150	53	78445-115089	Pseudo_B3_NC_006548	47.80%
A045	1	*Transposable*	*BcepMu-like*	*16*		933_AAPH		GCF_001059425.1_ASM105942v1	33.5	intact	120	48	134299-167897	Burkho_BcepMu_NC_005882	44.14%
A046	1	*Lambda*	*HK97-like*	*32*		NJ8700		GCF_001188835.1_ASM118883v1	49.3	intact	150	62	462213-511604	Mannhe_vB_MhS_1152AP2_NC_028956	43.07%
A046	2					NJ8700		GCF_001188835.1_ASM118883v1	23.9	incomplete	60	20	2250100-2274046	Burkho_BcepMu_NC_005882	41.39%
A047	1	*Transposable*	*BcepMu-like*	*16*		W10433		GCF_001262035.1_ASM126203v1	36.9	intact	120	53	754163-791141	Burkho_phiE255_NC_009237	44.56%
A048	1	*Transposable*	*Mu-like*	*11*		ATCC 19415		GCF_001680765.1_ASM168076v1	33.9	intact	140	45	12060-46042	Escher_D108_NC_013594	47.23%
A048	2					ATCC 19415		GCF_001680765.1_ASM168076v1	23.9	incomplete	50	20	44329-68300	Burkho_BcepMu_NC_005882	41.18%
A049	1					ATCC 7901		GCF_001680805.1_ASM168080v1	26.5	incomplete	40	19	361726-388297	Burkho_BcepMu_NC_005882	41.75%
H001	1				*Haemophilus influenzae*	Rd KW20	2	GCF_000027305.1_ASM2730v1	18.2	incomplete	50	24	1495049-1513250	Acinet_vB_AbaS_TRS1_NC_031098	39.26%
H001	2	*Transposable*	*Mu-like*	*07*	*Haemophilus influenzae*	Rd KW20	2	GCF_000027305.1_ASM2730v1	38.4	intact	140	53	1558774-1597183	Escher_D108_NC_013594	43.92%
H001	3				*Haemophilus influenzae*	Rd KW20	2	GCF_000027305.1_ASM2730v1	6.4	incomplete	50	8	1636791-1643216	Mannhe_vB_MhM_3927AP2_NC_02876[illegible]	39.96%
H002	1				*Haemophilus influenzae*	86-028NP	1	GCF_000012185.1_ASM1218v1	11.4	incomplete	20	13	124955-136402	Salmon_SJ46_NC_031129	43.09%
H002	2	*Lambda*	*DIBBI-like*	*23*	*Haemophilus influenzae*	86-028NP	1	GCF_000012185.1_ASM1218v1	80.5	intact	150	77	1394645-1475226	Pseudo_JBD25_NC_027992	40.02%
H002	3				*Haemophilus influenzae*	86-028NP	1	GCF_000012185.1_ASM1218v1	17.9	questionable	70	31	1579927-1597851	Haemop_Aaphi23_NC_004827	38.61%
H002	4				*Haemophilus influenzae*	86-028NP	1	GCF_000012185.1_ASM1218v1	20.8	intact	150	30	1707312-1728142	Mannhe_phiMHaA1_NC_008201	40.18%
H003	1				*Haemophilus influenzae*	3655	3	GCF_000169775.1_ASM16977v1	24.4	incomplete	60	33	508-24913	Burkho_phiE255_NC_009237	39.90%
H003	2				*Haemophilus influenzae*	3655	3	GCF_000169775.1_ASM16977v1	26.3	intact	140	37	512-26859	Escher_D108_NC_013594	44.19%
H003	3				*Haemophilus influenzae*	3655	3	GCF_000169775.1_ASM16977v1	10.9	incomplete	30	16	86081-97078	Phormi_MIS_PhV1A_NC_029032	37.92%
H003	4				*Haemophilus influenzae*	3655	3	GCF_000169775.1_ASM16977v1	19	incomplete	50	34	37295-56378	Aggreg_S1249_NC_013597	39.14%

TABLE S2

Strain ID	Region	Type	Supercluster	Cluster	Species	Strain	Clade	Refseq/Genbank	Region Length	Completeness	Score	# Total Proteins	Region Position	Most Common Phage	GC %
H003	5				*Haemophilus influenzae*	3655	3	GCF_000169775.1_ASM16977v1	15.5	questionable	90	19	226426-242012	Shigel_SfIV_NC_022749	38.03%
H004	1				*Haemophilus influenzae*	22.01.2021		GCF_000169735.1_ASM16973v1	27.1	questionable	90	43	5813-32924	Aggreg_S1249_NC_013597	42.08%
H004	2				*Haemophilus influenzae*	22.01.2021		GCF_000169735.1_ASM16973v1	8.4	incomplete	60	17	36534-44933	Haemop_SuMu_NC_019455	39.51%
H004	3				*Haemophilus influenzae*	22.01.2021		GCF_000169735.1_ASM16973v1	35.2	questionable	90	40	75415-110649	Haemop_Aaphi23_NC_004827	38.35%
H005	1	*Lambda*	*Aaphi23-like*	*24*	*Haemophilus influenzae*	R3021		GCF_000169755.1_ASM16975v1	27.5	intact	106	41	2-27529	Aggreg_S1249_NC_013597	40.63%
H005	2				*Haemophilus influenzae*	R3021		GCF_000169755.1_ASM16975v1	15.5	intact	150	22	65849-81408	Mannhe_vB_MhM_587AP1_NC_028898	40.30%
H005	3				*Haemophilus influenzae*	R3021		GCF_000169755.1_ASM16975v1	21.5	incomplete	30	13	152851-174425	Mannhe_vB_MhS_587AP2_NC_028743	38.29%
H006	1				*Haemophilus influenzae*	PittAA	3	GCF_000169795.1_ASM16979v1	10	incomplete	30	18	3-10087	Mannhe_vB_MhM_3927AP2_NC_02876[illegible]	40.64%
H006	2				*Haemophilus influenzae*	PittAA	3	GCF_000169795.1_ASM16979v1	10	incomplete	30	21	2-10047	Pseudo_JBD25_NC_027992	44.17%
H006	3				*Haemophilus influenzae*	PittAA	3	GCF_000169795.1_ASM16979v1	21.3	intact	150	33	3-21398	Acinet_vB_AbaS_TRS1_NC_031098	41.77%
H006	4				*Haemophilus influenzae*	PittAA	3	GCF_000169795.1_ASM16979v1	23.6	questionable	70	32	1-23665	Burkho_BcepMu_NC_005882	44.94%
H006	5				*Haemophilus influenzae*	PittAA	3	GCF_000169795.1_ASM16979v1	25	questionable	70	35	1356-26374	Burkho_BcepMu_NC_005882	39.79%
H006	6				*Haemophilus influenzae*	PittAA	3	GCF_000169795.1_ASM16979v1	4.9	incomplete	20	7	65793-70740	Entero_SfMu_NC_027382	41.17%
H007	1				*Haemophilus influenzae*	PittII	5	GCF_000169835.1_ASM16983v1	3.6	incomplete	50	8	2200-5896	Haemop_SuMu_NC_019455	39.14%
H007	2				*Haemophilus influenzae*	PittII	5	GCF_000169835.1_ASM16983v1	24	intact	150	28	164-24216	Entero_c_1_NC_019706	42.09%
H007	3	*P1*	*MHaA1-like*	*19*	*Haemophilus influenzae*	PittII	5	GCF_000169835.1_ASM16983v1	32.1	intact	150	46	1924-34112	Mannhe_phiMHaA1_NC_008201	41.02%
H007	4				*Haemophilus influenzae*	PittII	5	GCF_000169835.1_ASM16983v1	29	intact	129	39	89-29109	Haemop_HP2_NC_003315	39.90%
H007	5				*Haemophilus influenzae*	PittII	5	GCF_000169835.1_ASM16983v1	22.2	incomplete	40	26	56138-78414	Salmon_SJ46_NC_031129	39.13%
H007	6				*Haemophilus influenzae*	PittII	5	GCF_000169835.1_ASM16983v1	35.2	questionable	80	38	133754-168990	Haemop_Aaphi23_NC_004827	38.36%
H008	1	*Transposable*	*BcepMu-like*	*14*	*Haemophilus influenzae*	PittHH		GCF_000169815.1_ASM16981v1	22.5	intact	100	40	35500-58079	Burkho_BcepMu_NC_005882	41.19%
H008	2	*P1*	*MHaA1-like*	*19*	*Haemophilus influenzae*	PittHH		GCF_000169815.1_ASM16981v1	27.6	intact	150	40	5381-33021	Mannhe_vB_MhM_587AP1_NC_028898	40.49%
H008	3				*Haemophilus influenzae*	PittHH		GCF_000169815.1_ASM16981v1	11.6	incomplete	30	19	74204-85822	Haemop_Aaphi23_NC_004827	38.99%
H009	1				*Haemophilus influenzae*	22.04.2021		GCF_000169855.1_ASM16985v1	17.3	intact	150	27	11756-29095	Mannhe_vB_MhM_587AP1_NC_028898	41.30%
H009	2				*Haemophilus influenzae*	22.04.2021		GCF_000169855.1_ASM16985v1	26.8	incomplete	50	26	627-27466	Mannhe_vB_MhS_1152AP2_NC_028956	36.56%
H010	1				*Haemophilus influenzae*	PittEE	3	GCF_000016465.1_ASM1646v1	12.9	incomplete	40	20	958225-971182	Mannhe_vB_MhS_587AP2_NC_028743	36.67%
H010	2				*Haemophilus influenzae*	PittEE	3	GCF_000016465.1_ASM1646v1	25.6	incomplete	30	9	1072930-1098530	Haemop_SuMu_NC_019455	37.85%
H011	1				*Haemophilus influenzae*	PittGG	2	GCF_000016485.1_ASM1648v1	5.9	incomplete	50	9	6238-12187	Mannhe_vB_MhM_3927AP2_NC_02876[illegible]	39.68%
H011	2	*Lambda*	*Aaphi23-like*	*24*	*Haemophilus influenzae*	PittGG	2	GCF_000016485.1_ASM1648v1	24.7	intact	110	42	146438-171204	Acinet_vB_AbaS_TRS1_NC_031098	38.22%
H011	3	*Lambda*	*DIBBI-like*	*23*	*Haemophilus influenzae*	PittGG	2	GCF_000016485.1_ASM1648v1	56.7	intact	130	69	868570-925310	Pseudo_phi2_NC_030931	40.51%
H011	4				*Haemophilus influenzae*	PittGG	2	GCF_000016485.1_ASM1648v1	13.7	incomplete	30	18	1775603-1789345	Caulob_Cr30_NC_025422	37.79%
H012	1				*Haemophilus influenzae*	7P49H1	3	GCF_000173315.1_ASM17331v1	25.6	incomplete	30	9	19940-45544	Haemop_SuMu_NC_019455	37.80%
H012	2				*Haemophilus influenzae*	7P49H1	3	GCF_000173315.1_ASM17331v1	11.8	incomplete	40	22	31339-43237	Mannhe_vB_MhS_1152AP2_NC_028956	37.87%

TABLE S2

Strain ID	Region	Type	Supercluster	Cluster	Species	Strain	Clade	Refseq/Genbank	Region Length	Completeness	Score	# Total Proteins	Region Position	Most Common Phage	GC %
H013	1	*P1*	*MHaA1-like*	*19*	*Haemophilus influenzae*	6P18H1	3	GCF_000173335.1_ASM17333v1	33.7	intact	150	40	735-34499	Mannhe_phiMHaA1_NC_008201	40.42%
H013	2	*Transposable*	*SuMu-like*	*01*	*Haemophilus influenzae*	6P18H1	3	GCF_000173335.1_ASM17333v1	43.1	intact	150	60	305-43482	Mannhe_vB_MhM_3927AP2_NC_02876[illegible]	41.55%
H013	3	*Lambda*	*DIBBI-like*	*23*	*Haemophilus influenzae*	6P18H1	3	GCF_000173335.1_ASM17333v1	49.7	intact	140	81	200208-249943	Pseudo_vB_PaeM_MAG1_NC_031073	40.69%
H013	4				*Haemophilus influenzae*	6P18H1	3	GCF_000173335.1_ASM17333v1	19	incomplete	60	35	254120-273163	Aggreg_S1249_NC_013597	39.16%
H014	1	*Lambda*	*DIBBI-like*	*23*	*Haemophilus influenzae*	NT127	3	GCF_000175435.1_ASM17543v1	42.1	intact	140	70	1-42100	Aggreg_S1249_NC_013597	41.24%
H014	2				*Haemophilus influenzae*	NT127	3	GCF_000175435.1_ASM17543v1	11.4	incomplete	20	19	12001-23458	Aggreg_S1249_NC_013597	38.46%
H014	3				*Haemophilus influenzae*	NT127	3	GCF_000175435.1_ASM17543v1	15	intact	140	20	1929-16936	Mannhe_vB_MhM_587AP1_NC_028898	41.17%
H014	4				*Haemophilus influenzae*	NT127	3	GCF_000175435.1_ASM17543v1	14.1	intact	100	23	62893-77083	Mannhe_phiMHaA1_NC_008201	40.89%
H014	5				*Haemophilus influenzae*	NT127	3	GCF_000175435.1_ASM17543v1	6.4	incomplete	40	8	126642-133099	Mannhe_vB_MhM_3927AP2_NC_02876[illegible]	39.27%
H015	1				*Haemophilus influenzae*	RdAW	2	GCF_000175455.1_ASM17545v1	14.4	incomplete	60	25	31735-46134	Aggreg_S1249_NC_013597	39.54%
H015	2	*Transposable*	*Mu-like*	*07*	*Haemophilus influenzae*	RdAW	2	GCF_000175455.1_ASM17545v1	41.4	intact	140	57	8886-50309	Escher_D108_NC_013594	43.70%
H015	3				*Haemophilus influenzae*	RdAW	2	GCF_000175455.1_ASM17545v1	6.4	incomplete	50	9	86900-93326	Mannhe_vB_MhM_3927AP2_NC_02876[illegible]	39.96%
H016	-				*Haemophilus influenzae*	HK1212	?	GCA_000171475.1_ASM17147v1	-	no prophage	-	-	-	-	-
H017	1				*Haemophilus influenzae*	10810	2	GCF_000210875.1_ASM21087v1	11.4	incomplete	20	13	119906-131353	Salmon_SJ46_NC_031129	43.09%
H017	2	*Lambda*	*Gifsy2-like*	*31*	*Haemophilus influenzae*	10810	2	GCF_000210875.1_ASM21087v1	41.6	intact	150	58	357152-398803	Mannhe_vB_MhS_587AP2_NC_028743	40.80%
H017	3				*Haemophilus influenzae*	10810	2	GCF_000210875.1_ASM21087v1	15.4	questionable	70	20	561971-577394	Shigel_SfIV_NC_022749	40.00%
H017	4	*Lambda*	*Aaphi23-like*	*24*	*Haemophilus influenzae*	10810	2	GCF_000210875.1_ASM21087v1	35.2	intact	110	57	1644619-1679868	Acinet_vB_AbaS_TRS1_NC_031098	40.30%
H017	5				*Haemophilus influenzae*	10810	2	GCF_000210875.1_ASM21087v1	31	intact	120	24	1750054-1781151	Haemop_SuMu_NC_019455	39.84%
H017	6				*Haemophilus influenzae*	10810	2	GCF_000210875.1_ASM21087v1	19.2	incomplete	50	18	1782258-1801529	Mannhe_vB_MhS_587AP2_NC_028743	38.38%
H018	1	*P1*	*HP1-like*	*21*	*Haemophilus influenzae*	R2866		GCF_000165525.1_ASM16552v1	47.1	intact	145	44	523442-570597	Haemop_HP2_NC_003315	38.42%
H018	2				*Haemophilus influenzae*	R2866		GCF_000165525.1_ASM16552v1	11.4	incomplete	20	13	564225-575672	Salmon_SJ46_NC_031129	43.20%
H018	3				*Haemophilus influenzae*	R2866		GCF_000165525.1_ASM16552v1	35.2	questionable	90	37	982762-1017992	Haemop_Aaphi23_NC_004827	38.36%
H018	4				*Haemophilus influenzae*	R2866		GCF_000165525.1_ASM16552v1	5.9	incomplete	40	7	1122433-1128426	Mannhe_vB_MhM_3927AP2_NC_02876[illegible]	39.64%
H018	5	*P1*	*MHaA1-like*	*19*	*Haemophilus influenzae*	R2866		GCF_000165525.1_ASM16552v1	34.3	intact	150	43	1741094-1775480	Mannhe_phiMHaA1_NC_008201	40.60%
H019	1				*Haemophilus influenzae*	R2846	3	GCF_000165575.1_ASM16557v1	13.3	incomplete	20	19	942826-956202	Mannhe_vB_MhS_1152AP2_NC_028956	38.44%
H019	2				*Haemophilus influenzae*	R2846	3	GCF_000165575.1_ASM16557v1	28.7	incomplete	30	8	1076524-1105272	Haemop_SuMu_NC_019455	37.57%
H020	1	*P1*	*HP1-like*	*21*	*Haemophilus influenzae*	F3031	2	GCF_000197875.1_ASM19787v1	45.4	intact	130	48	72399-117875	Haemop_HP1_NC_001697	39.84%
H020	2	*Lambda*	*DIBBI-like*	*23*	*Haemophilus influenzae*	F3031	2	GCF_000197875.1_ASM19787v1	57.8	intact	140	69	313930-371759	Strept_phiARI0468_1_NC_031929	40.50%
H020	3	*Transposable*	*BcepMu-like*	*14*	*Haemophilus influenzae*	F3031	2	GCF_000197875.1_ASM19787v1	36.9	intact	110	53	415952-452944	Burkho_BcepMu_NC_005882	39.70%
H020	4	*Lambda*	*Aaphi23-like*	*24*	*Haemophilus influenzae*	F3031	2	GCF_000197875.1_ASM19787v1	58.9	intact	150	84	854475-913427	Aggreg_S1249_NC_013597	39.09%
H020	5				*Haemophilus influenzae*	F3031	2	GCF_000197875.1_ASM19787v1	16.5	incomplete	30	17	1021125-1037690	Bacill_BCD7_NC_019515	38.20%
H020	6				*Haemophilus influenzae*	F3031	2	GCF_000197875.1_ASM19787v1	20.5	questionable	70	34	1249277-1269853	Mannhe_vB_MhM_3927AP2_NC_02876[illegible]	42.79%

TABLE S2

Strain ID	Region	Type	Supercluster	Cluster	Species	Strain	Clade	Refseq/Genbank	Region Length	Completeness	Score	# Total Proteins	Region Position	Most Common Phage	GC %
H021	1	*P1*	*MHaA1-like*	*19*	*Haemophilus influenzae*	F3047	2	GCF_000200475.1_ASM20047v1	37.5	intact	150	51	454123-491635	Mannhe_vB_MhM_587AP1_NC_028898	40.18%
H021	2	*Lambda*	*DIBBI-like*	*23*	*Haemophilus influenzae*	F3047	2	GCF_000200475.1_ASM20047v1	48	intact	140	71	829118-877204	Aggreg_S1249_NC_013597	40.98%
H021	3	*Lambda*	*P22-like*	*36*	*Haemophilus influenzae*	F3047	2	GCF_000200475.1_ASM20047v1	55.2	intact	130	52	1366194-1421440	Mannhe_vB_MhS_587AP2_NC_028743	39.41%
H021	4	*Lambda*	*Aaphi23-like*	*24*	*Haemophilus influenzae*	F3047	2	GCF_000200475.1_ASM20047v1	53	intact	150	75	1641362-1694394	Aggreg_S1249_NC_013597	39.27%
H021	5				*Haemophilus influenzae*	F3047	2	GCF_000200475.1_ASM20047v1	16.6	incomplete	30	17	1803718-1820347	Bacill_BCD7_NC_019515	38.23%
H021	6				*Haemophilus influenzae*	F3047	2	GCF_000200475.1_ASM20047v1	19.5	incomplete	60	31	1953339-1972847	Mannhe_vB_MhM_3927AP2_NC_02876[illegible]	42.55%
H022	1				*Haemophilus influenzae*	KR494	4	GCF_000465255.1_ASM46525v1	28.5	intact	150	36	694054-722576	Mannhe_vB_MhM_3927AP2_NC_02876[illegible]	42.44%
H022	2				*Haemophilus influenzae*	KR494	4	GCF_000465255.1_ASM46525v1	11.3	intact	120	17	1377969-1389278	Mannhe_vB_MhM_587AP1_NC_028898	39.00%
H023	1				*Haemophilus influenzae*	584	3	GCF_000636035.1_NTHi584v1	6.7	incomplete	10	16	88278-95040	Aggreg_S1249_NC_013597	38.30%
H023	2	*Transposable*	*SuMu-like*	*03*	*Haemophilus influenzae*	584	3	GCF_000636035.1_NTHi584v1	35	intact	150	49	98409-133418	Haemop_SuMu_NC_019455	44.15%
H023	3				*Haemophilus influenzae*	584	3	GCF_000636035.1_NTHi584v1	6.7	questionable	80	12	3718-10453	Mannhe_vB_MhM_3927AP2_NC_02876[illegible]	39.68%
H024	1	*Transposable*	*SuMu-like*	*03*	*Haemophilus influenzae*	411	3	GCF_000636055.1_NTHi411v1	40.3	intact	150	49	248505-288897	Haemop_SuMu_NC_019455	43.53%
H024	2				*Haemophilus influenzae*	411	3	GCF_000636055.1_NTHi411v1	6.7	incomplete	10	16	272832-279594	Aggreg_S1249_NC_013597	38.30%
H024	3				*Haemophilus influenzae*	411	3	GCF_000636055.1_NTHi411v1	6.7	questionable	80	12	369732-376467	Mannhe_vB_MhM_3927AP2_NC_02876[illegible]	39.68%
H025	1				*Haemophilus influenzae*	1104	3	GCF_000636075.1_NTHi1104v1	13.1	questionable	80	15	45520-58620	Mannhe_vB_MhM_3927AP2_NC_02876[illegible]	37.17%
H025	2	*Transposable*	*SuMu-like*	*03*	*Haemophilus influenzae*	1104	3	GCF_000636075.1_NTHi1104v1	35	intact	150	48	31662-66671	Haemop_SuMu_NC_019455	44.16%
H025	3				*Haemophilus influenzae*	1104	3	GCF_000636075.1_NTHi1104v1	6.7	incomplete	10	16	88847-95609	Aggreg_S1249_NC_013597	38.30%
H026	1				*Haemophilus influenzae*	CGSHiCZ412602	3	GCF_000698365.1_ASM69836v1	18.8	incomplete	60	32	1085782-1104669	Aggreg_S1249_NC_013597	39.65%
H026	2				*Haemophilus influenzae*	CGSHiCZ412602	3	GCF_000698365.1_ASM69836v1	26.4	incomplete	30	7	1211689-1238156	Haemop_SuMu_NC_019455	37.57%
H027	1	*Lambda*	*Gifsy2-like*	*31*	*Haemophilus influenzae*	Hi375	1	GCF_000767075.1_ASM76707v1	48.6	intact	150	68	642088-690717	Salmon_103203_sal5_NC_031946	39.86%
H027	2				*Haemophilus influenzae*	Hi375	1	GCF_000767075.1_ASM76707v1	21.3	intact	150	31	1286089-1307476	Mannhe_phiMHaA1_NC_008201	40.04%
H027	3				*Haemophilus influenzae*	Hi375	1	GCF_000767075.1_ASM76707v1	17.1	questionable	70	28	1418021-1435201	Haemop_Aaphi23_NC_004827	38.59%
H028	1				*Haemophilus influenzae*	60294N1	3	GCF_000818925.1_ASM81892v1	23.5	incomplete	30	21	115711-139238	Mannhe_vB_MhS_535AP2_NC_028853	38.41%
H028	2	*Transposable*	*SuMu-like*	*01*	*Haemophilus influenzae*	60294N1	3	GCF_000818925.1_ASM81892v1	36.8	intact	150	51	86-36894	Haemop_SuMu_NC_019455	42.31%
H028	3				*Haemophilus influenzae*	60294N1	3	GCF_000818925.1_ASM81892v1	6.4	incomplete	50	9	252891-259345	Mannhe_vB_MhM_3927AP2_NC_02876[illegible]	39.92%
H029	1				*Haemophilus influenzae*	RMHi93	3	GCF_000833735.1_ASM83373v1	23	incomplete	30	20	9347-32376	Mannhe_vB_MhS_587AP2_NC_028743	36.80%
H029	2				*Haemophilus influenzae*	RMHi93	3	GCF_000833735.1_ASM83373v1	15.2	incomplete	60	27	1447-16653	Strept_phiARI0462_NC_031942	39.98%
H029	3				*Haemophilus influenzae*	RMHi93	3	GCF_000833735.1_ASM83373v1	14.8	incomplete	30	7	33354-48168	Haemop_SuMu_NC_019455	38.27%
H029	4				*Haemophilus influenzae*	RMHi93	3	GCF_000833735.1_ASM83373v1	38.1	intact	96	38	64917-103075	Salmon_BP63_NC_031250	39.21%
H029	5				*Haemophilus influenzae*	RMHi93	3	GCF_000833735.1_ASM83373v1	17.1	intact	150	24	73729-90885	Mannhe_phiMHaA1_NC_008201	40.33%
H029	6	*Transposable*	*BcepMu-like*	*14*	*Haemophilus influenzae*	RMHi93	3	GCF_000833735.1_ASM83373v1	27.8	intact	110	43	31293-59175	Burkho_BcepMu_NC_005882	40.59%
H030	1				*Haemophilus influenzae*	MiHi64	3	GCF_000833745.1_ASM83374v1	12.4	incomplete	40	19	3-12498	Aggreg_S1249_NC_013597	38.93%

TABLE S2

Strain ID	Region	Type	Supercluster	Cluster	Species	Strain	Clade	Refseq/Genbank	Region Length	Completeness	Score	# Total Proteins	Region Position	Most Common Phage	GC %
H030	2	*Transposable*	*SuMu-like*	*01*	*Haemophilus influenzae*	MiHi64	3	GCF_000833745.1_ASM83374v1	46	intact	150	58	14096-60108	Haemop_SuMu_NC_019455	41.50%
H031	1				*Haemophilus influenzae*	MiHi270	1	GCF_000833755.1_ASM83375v1	6.9	incomplete	50	16	108-7007	Aggreg_S1249_NC_013597	39.75%
H031	2				*Haemophilus influenzae*	MiHi270	1	GCF_000833755.1_ASM83375v1	12.2	incomplete	50	20	79763-92006	Haemop_Aaphi23_NC_004827	39.33%
H031	3				*Haemophilus influenzae*	MiHi270	1	GCF_000833755.1_ASM83375v1	5.9	incomplete	40	8	24851-30772	Mannhe_vB_MhM_3927AP2_NC_02876	39.40%
H031	4				*Haemophilus influenzae*	MiHi270	1	GCF_000833755.1_ASM83375v1	11.1	incomplete	10	16	50826-61976	Gordon_Bowser_NC_030930	38.28%
H031	5				*Haemophilus influenzae*	MiHi270	1	GCF_000833755.1_ASM83375v1	9.7	intact	110	16	514-10285	Mannhe_vB_MhM_587AP1_NC_028898	40.86%
H031	6				*Haemophilus influenzae*	MiHi270	1	GCF_000833755.1_ASM83375v1	18.6	incomplete	50	26	383-18995	Mannhe_vB_MhS_587AP2_NC_028743	39.98%
H032	1				*Haemophilus influenzae*	Hi381	1	GCF_000833765.1_ASM83376v1	10.9	incomplete	50	18	79789-90756	Haemop_Aaphi23_NC_004827	39.06%
H032	2	*Lambda*	*Gifsy2-like*	*31*	*Haemophilus influenzae*	Hi381	1	GCF_000833765.1_ASM83376v1	31.7	intact	150	43	587-32343	Aggreg_S1249_NC_013597	40.32%
H032	3				*Haemophilus influenzae*	Hi381	1	GCF_000833765.1_ASM83376v1	27.7	incomplete	40	15	102932-130694	Mannhe_vB_MhM_3927AP2_NC_02876	38.23%
H032	4				*Haemophilus influenzae*	Hi381	1	GCF_000833765.1_ASM83376v1	21.3	intact	150	30	102758-124089	Mannhe_vB_MhM_587AP1_NC_028898	40.02%
H032	5				*Haemophilus influenzae*	Hi381	1	GCF_000833765.1_ASM83376v1	8	incomplete	40	12	1041-9099	Pseudo_phi2_NC_030931	40.96%
H033	1				*Haemophilus influenzae*	Hi361	1	GCF_000833815.1_ASM83381v1	27.7	incomplete	40	15	27291-55054	Mannhe_vB_MhM_3927AP2_NC_02876	38.23%
H033	2				*Haemophilus influenzae*	Hi361	1	GCF_000833815.1_ASM83381v1	9.7	intact	110	16	515-10286	Mannhe_vB_MhM_587AP1_NC_028898	40.86%
H033	3				*Haemophilus influenzae*	Hi361	1	GCF_000833815.1_ASM83381v1	15.9	incomplete	40	20	394-16304	Aggreg_S1249_NC_013597	37.17%
H033	4	*Lambda*	*Gifsy2-like*	*31*	*Haemophilus influenzae*	Hi361	1	GCF_000833815.1_ASM83381v1	38.9	intact	150	44	195596-234517	Pseudo_MD8_NC_031091	40.16%
H034	1	*Transposable*	*BcepMu-like*	*14*	*Haemophilus influenzae*	Hi322	3	GCF_000833835.1_ASM83383v1	28	intact	110	42	223789-251822	Burkho_BcepMu_NC_005882	40.57%
H034	2				*Haemophilus influenzae*	Hi322	3	GCF_000833835.1_ASM83383v1	25.9	intact	150	37	5863-31849	Mannhe_vB_MhM_587AP1_NC_028898	41.21%
H034	3	*Lambda*	*Gifsy2-like*	*31*	*Haemophilus influenzae*	Hi322	3	GCF_000833835.1_ASM83383v1	54.9	intact	150	56	16-54920	Strept_phiARI0468_4_NC_031915	39.30%
H034	4				*Haemophilus influenzae*	Hi322	3	GCF_000833835.1_ASM83383v1	15.7	incomplete	20	8	33358-49141	Haemop_SuMu_NC_019455	38.29%
H034	5				*Haemophilus influenzae*	Hi322	3	GCF_000833835.1_ASM83383v1	11.6	incomplete	30	18	75397-87037	Haemop_Aaphi23_NC_004827	38.97%
H035	1				*Haemophilus influenzae*	Hi359	1	GCF_000833855.1_ASM83385v1	9.7	intact	110	18	513-10256	Mannhe_vB_MhM_587AP1_NC_028898	40.90%
H035	2				*Haemophilus influenzae*	Hi359	1	GCF_000833855.1_ASM83385v1	10.9	incomplete	50	20	2-10988	Aggreg_S1249_NC_013597	39.62%
H035	3				*Haemophilus influenzae*	Hi359	1	GCF_000833855.1_ASM83385v1	5.9	incomplete	40	8	24899-30820	Mannhe_vB_MhM_3927AP2_NC_02876	39.38%
H035	4				*Haemophilus influenzae*	Hi359	1	GCF_000833855.1_ASM83385v1	8.1	incomplete	20	14	74357-82514	Acinet_LZ35_NC_031117	39.16%
H036	1				*Haemophilus influenzae*	Hi345	3	GCF_000833875.1_ASM83387v1	12.9	incomplete	40	22	80947-93897	Mannhe_vB_MhS_1152AP2_NC_028956	36.72%
H036	2				*Haemophilus influenzae*	Hi345	3	GCF_000833875.1_ASM83387v1	25.3	incomplete	30	10	40423-65811	Haemop_SuMu_NC_019455	37.71%
H037	1				*Haemophilus influenzae*	Hi378	1	GCF_000833895.1_ASM83389v1	20.6	questionable	70	36	81971-102591	Acinet_LZ35_NC_031117	38.55%
H037	2				*Haemophilus influenzae*	Hi378	1	GCF_000833895.1_ASM83389v1	5.4	incomplete	20	11	2-5472	Mannhe_vB_MhS_535AP2_NC_028853	38.69%
H037	3	*Lambda*	*Gifsy2-like*	*31*	*Haemophilus influenzae*	Hi378	1	GCF_000833895.1_ASM83389v1	38.2	intact	150	51	1-38272	Pseudo_phi2_NC_030931	41.00%
H037	4				*Haemophilus influenzae*	Hi378	1	GCF_000833895.1_ASM83389v1	25.3	intact	150	39	35571-60895	Mannhe_vB_MhM_587AP1_NC_028898	40.45%
H037	5				*Haemophilus influenzae*	Hi378	1	GCF_000833895.1_ASM83389v1	34.9	incomplete	30	25	29812-64765	Salmon_SJ46_NC_031129	38.67%

TABLE S2

Strain ID	Region	Type	Supercluster	Cluster	Species	Strain	Clade	Refseq/Genbank	Region Length	Completeness	Score	# Total Proteins	Region Position	Most Common Phage	GC %
H038	1				*Haemophilus influenzae*	Hi394	1	GCF_000833915.1_ASM83391v1	9.5	incomplete	30	16	1-9510	Mannhe_vB_MhS_587AP2_NC_028743	37.16%
H038	2				*Haemophilus influenzae*	Hi394	1	GCF_000833915.1_ASM83391v1	9.7	incomplete	30	17	1384-11100	Salmon_118970_sal3_NC_031940	38.25%
H038	3				*Haemophilus influenzae*	Hi394	1	GCF_000833915.1_ASM83391v1	7.8	incomplete	50	12	2-7802	Haemop_SuMu_NC_019455	39.28%
H038	4	*Transposable*	*BcepMu-like*	*14*	*Haemophilus influenzae*	Hi394	1	GCF_000833915.1_ASM83391v1	36.1	intact	120	52	202788-238922	Burkho_phiE255_NC_009237	39.69%
H038	5				*Haemophilus influenzae*	Hi394	1	GCF_000833915.1_ASM83391v1	22.3	intact	150	27	540-22930	Entero_c_1_NC_019706	42.25%
H038	6				*Haemophilus influenzae*	Hi394	1	GCF_000833915.1_ASM83391v1	16.5	intact	150	26	2241-18838	Mannhe_phiMHaA1_NC_008201	40.13%
H038	7				*Haemophilus influenzae*	Hi394	1	GCF_000833915.1_ASM83391v1	11	incomplete	50	20	468-11535	Aggreg_S1249_NC_013597	39.61%
H039	1				*Haemophilus influenzae*	Hi403	1	GCF_000833935.1_ASM83393v1	20.9	incomplete	30	12	170158-191135	Mannhe_vB_MhS_587AP2_NC_028743	38.44%
H039	2				*Haemophilus influenzae*	Hi403	1	GCF_000833935.1_ASM83393v1	11.2	incomplete	30	21	55-11343	Gordon_GMA4_NC_030939	39.55%
H039	3				*Haemophilus influenzae*	Hi403	1	GCF_000833935.1_ASM83393v1	7.5	incomplete	20	13	74298-81819	Mannhe_vB_MhS_535AP2_NC_028853	39.13%
H039	4				*Haemophilus influenzae*	Hi403	1	GCF_000833935.1_ASM83393v1	18.9	intact	150	26	101859-120787	Mannhe_phiMHaA1_NC_008201	40.45%
H039	5				*Haemophilus influenzae*	Hi403	1	GCF_000833935.1_ASM83393v1	5.9	incomplete	40	8	1514-7436	Mannhe_vB_MhM_3927AP2_NC_02876	39.39%
H039	6				*Haemophilus influenzae*	Hi403	1	GCF_000833935.1_ASM83393v1	11	incomplete	50	20	2-11099	Aggreg_S1249_NC_013597	39.60%
H040	1				*Haemophilus influenzae*	1209	3	GCF_000877255.1_ASM87725v1	24.7	intact	150	32	602509-627253	Mannhe_vB_MhM_587AP1_NC_028898	40.94%
H040	2				*Haemophilus influenzae*	1209	3	GCF_000877255.1_ASM87725v1	11.6	incomplete	30	17	736367-747974	Haemop_Aaphi23_NC_004827	38.98%
H040	3	*Lambda*	*Gifsy2-like*	*31*	*Haemophilus influenzae*	1209	3	GCF_000877255.1_ASM87725v1	75.7	intact	150	75	862364-938130	Strept_phiARI0468_4_NC_031915	38.73%
H041	1				*Haemophilus influenzae*	477	1	GCF_000931575.1_ASM93157v1	5.9	incomplete	40	7	95536-101457	Mannhe_vB_MhM_3927AP2_NC_02876	39.41%
H041	2				*Haemophilus influenzae*	477	1	GCF_000931575.1_ASM93157v1	38.9	questionable	90	37	217192-256123	Haemop_Aaphi23_NC_004827	37.71%
H041	3				*Haemophilus influenzae*	477	1	GCF_000931575.1_ASM93157v1	21.3	intact	150	32	350901-372238	Mannhe_phiMHaA1_NC_008201	39.99%
H041	4	*Lambda*	*DIBBI-like*	*23*	*Haemophilus influenzae*	477	1	GCF_000931575.1_ASM93157v1	61	intact	150	73	1451503-1512517	Pseudo_phi2_NC_030931	39.80%
H042	1				*Haemophilus influenzae*	C486	3	GCF_000931605.1_ASM93160v1	25.6	incomplete	30	8	1156970-1182570	Haemop_SuMu_NC_019455	37.84%
H042	2				*Haemophilus influenzae*	C486	3	GCF_000931605.1_ASM93160v1	12.5	incomplete	40	14	1298748-1311330	Mannhe_vB_MhS_587AP2_NC_028743	37.09%
H042	3	*Transposable*	*SuMu-like*	*01*	*Haemophilus influenzae*	C486	3	GCF_000931605.1_ASM93160v1	57.4	intact	150	51	1671039-1728494	Mannhe_vB_MhM_3927AP2_NC_02876	40.35%
H043	1				*Haemophilus influenzae*	723	1	GCF_000931625.1_ASM93162v1	3.6	incomplete	50	7	2-3628	Haemop_SuMu_NC_019455	38.60%
H043	2				*Haemophilus influenzae*	723	1	GCF_000931625.1_ASM93162v1	17.8	questionable	70	32	124867-142743	Haemop_Aaphi23_NC_004827	38.61%
H043	3				*Haemophilus influenzae*	723	1	GCF_000931625.1_ASM93162v1	20	intact	150	28	252293-272298	Mannhe_phiMHaA1_NC_008201	40.34%
H043	4	*Transposable*	*SuMu-like*	*01*	*Haemophilus influenzae*	723	1	GCF_000931625.1_ASM93162v1	47.1	intact	150	60	867863-915025	Mannhe_vB_MhM_3927AP2_NC_02876	41.28%
H043	5	*Lambda*	*Gifsy2-like*	*31*	*Haemophilus influenzae*	723	1	GCF_000931625.1_ASM93162v1	51.9	intact	150	57	1835195-1887181	Pseudo_MD8_NC_031091	40.59%
H044	1	*Lambda*	*Gifsy2-like*	*31*	*Haemophilus influenzae*	NCTC8143	1	GCF_001457655.1_NCTC8143	49.5	intact	150	66	642039-691614	Salmon_103203_sal5_NC_031946	40.02%
H044	2	*Transposable*	*SuMu-like*	*01*	*Haemophilus influenzae*	NCTC8143	1	GCF_001457655.1_NCTC8143	43.8	intact	150	48	1011808-1055698	Haemop_SuMu_NC_019455	41.48%
H044	3				*Haemophilus influenzae*	NCTC8143	1	GCF_001457655.1_NCTC8143	21.3	intact	150	30	1326271-1347647	Mannhe_phiMHaA1_NC_008201	40.04%
H044	4				*Haemophilus influenzae*	NCTC8143	1	GCF_001457655.1_NCTC8143	17.1	questionable	70	28	1458314-1475494	Haemop_Aaphi23_NC_004827	38.57%

TABLE S2

Strain ID	Region	Type	Supercluster	Cluster	Species	Strain	Clade	Refseq/Genbank	Region Length	Completeness	Score	# Total Proteins	Region Position	Most Common Phage	GC %
H044	5				*Haemophilus influenzae*	NCTC8143	1	GCF_001457655.1_NCTC8143	5.9	incomplete	40	7	1596623-1602545	Mannhe_vB_MhM_3927AP2_NC_02876	39.41%
H045	1	*Transposable*	*BcepMu-like*	*14*	*Haemophilus influenzae*	2019	3	GCF_000968335.1_ASM96833v1	33.5	intact	110	47	83591-117155	Burkho_phiE255_NC_009237	39.96%
H045	2	*Lambda*	*DIBBI-like*	*23*	*Haemophilus influenzae*	2019	3	GCF_000968335.1_ASM96833v1	58.5	intact	130	73	468253-526753	Salmon_118970_sal3_NC_031940	40.14%
H045	3	*Transposable*	*BcepMu-like*	*17*	*Haemophilus influenzae*	2019	3	GCF_000968335.1_ASM96833v1	43.9	intact	100	62	1267101-1311076	Burkho_BcepMu_NC_005882	43.66%
H045	4				*Haemophilus influenzae*	2019	3	GCF_000968335.1_ASM96833v1	18.1	incomplete	60	27	1616054-1634246	Aggreg_S1249_NC_013597	38.91%
H045	5	*P1*	*MHaA1-like*	*19*	*Haemophilus influenzae*	2019	3	GCF_000968335.1_ASM96833v1	41.9	intact	150	47	1740482-1782440	Mannhe_phiMHaA1_NC_008201	40.60%
H046	1	*Transposable*	*BcepMu-like*	*17*	*Haemophilus influenzae*	2019	3	GCF_001007605.1_2019	32.9	intact	110	47	1-32912	Burkho_phiE255_NC_009237	39.95%
H046	2	*P1*	*MHaA1-like*	*19*	*Haemophilus influenzae*	2019	3	GCF_001007605.1_2019	41.9	intact	150	44	103345-145313	Mannhe_phiMHaA1_NC_008201	40.58%
H046	3				*Haemophilus influenzae*	2019	3	GCF_001007605.1_2019	15.5	incomplete	60	30	37782-53355	Aggreg_S1249_NC_013597	39.01%
H046	4	*Lambda*	*DIBBI-like*	*23*	*Haemophilus influenzae*	2019	3	GCF_001007605.1_2019	45.1	intact	130	73	99-45273	Salmon_118970_sal3_NC_031940	40.75%
H046	5				*Haemophilus influenzae*	2019	3	GCF_001007605.1_2019	35.8	questionable	90	49	99-35898	Burkho_BcepMu_NC_005882	44.44%
H047	1				*Haemophilus influenzae*	1057_HINF	1	GCF_001053795.1_ASM105379v1	5.9	incomplete	40	9	23248-29168	Mannhe_vB_MhM_3927AP2_NC_02876	39.39%
H047	2				*Haemophilus influenzae*	1057_HINF	1	GCF_001053795.1_ASM105379v1	16.5	intact	150	26	10948-27509	Mannhe_vB_MhM_587AP1_NC_028898	40.08%
H047	3				*Haemophilus influenzae*	1057_HINF	1	GCF_001053795.1_ASM105379v1	12.4	incomplete	20	20	262-12760	Acinet_LZ35_NC_031117	39.88%
H047	4				*Haemophilus influenzae*	1057_HINF	1	GCF_001053795.1_ASM105379v1	22.5	questionable	90	33	971-23542	Erwini_phiEt88_NC_015295	41.99%
H048	1				*Haemophilus influenzae*	1061_HINF	3	GCF_001053815.1_ASM105381v1	21	incomplete	50	27	336-21412	Mannhe_vB_MhS_587AP2_NC_028743	39.60%
H049	1				*Haemophilus influenzae*	552_HINF	3	GCF_001057195.1_ASM105719v1	21.9	incomplete	30	18	126022-147927	Salmon_118970_sal3_NC_031940	37.94%
H049	2	*P1*	*HP1-like*	*21*	*Haemophilus influenzae*	552_HINF	3	GCF_001057195.1_ASM105719v1	32.1	intact	140	43	89-32219	Haemop_HP2_NC_003315	40.71%
H049	3				*Haemophilus influenzae*	552_HINF	3	GCF_001057195.1_ASM105719v1	25.1	questionable	70	37	806-25987	Aggreg_S1249_NC_013597	42.04%
H049	4				*Haemophilus influenzae*	552_HINF	3	GCF_001057195.1_ASM105719v1	7.3	incomplete	60	8	3-7326	Haemop_SuMu_NC_019455	37.52%
H049	5	*Transposable*	*BcepMu-like*	*14*	*Haemophilus influenzae*	552_HINF	3	GCF_001057195.1_ASM105719v1	31.4	intact	110	45	458-31945	Burkho_BcepMu_NC_005882	39.96%
H049	6	*Transposable*	*SuMu-like*	*01*	*Haemophilus influenzae*	552_HINF	3	GCF_001057195.1_ASM105719v1	37.7	intact	150	53	454-38167	Mannhe_vB_MhM_3927AP2_NC_02876	41.76%
H049	7				*Haemophilus influenzae*	552_HINF	3	GCF_001057195.1_ASM105719v1	10.7	incomplete	40	16	69-10798	Aggreg_S1249_NC_013597	39.85%
H049	8				*Haemophilus influenzae*	552_HINF	3	GCF_001057195.1_ASM105719v1	32	incomplete	40	31	69-32100	Acinet_LZ35_NC_031117	38.20%
H050	1	*Lambda*	*Gifsy2-like*	*31*	*Haemophilus influenzae*	839_HINF		GCF_001059185.1_ASM105918v1	49.1	intact	150	78	1-49141	Mannhe_vB_MhS_587AP2_NC_028743	39.33%
H050	2				*Haemophilus influenzae*	839_HINF		GCF_001059185.1_ASM105918v1	17.1	questionable	70	24	29544-46700	Shigel_SfIV_NC_022749	37.55%
H051	1				*Haemophilus influenzae*	167_HINF	?	GCF_001071575.1_ASM107157v1	26.9	incomplete	30	25	35196-62138	Salmon_SJ46_NC_031129	38.48%
H052	1				*Haemophilus influenzae*	1059_HINF	3	GCF_001052725.1_ASM105272v1	26.4	incomplete	50	38	19745-46209	Mannhe_vB_MhS_587AP2_NC_028743	39.41%
H053	1				*Haemophilus influenzae*	1124_HINF	1	GCF_001053885.1_ASM105388v1	5.9	incomplete	40	8	23460-29381	Mannhe_vB_MhM_3927AP2_NC_02876	39.40%
H053	2				*Haemophilus influenzae*	1124_HINF	1	GCF_001053885.1_ASM105388v1	9.9	incomplete	30	16	1-9917	Pseudo_phi2_NC_030931	39.34%
H053	3				*Haemophilus influenzae*	1124_HINF	1	GCF_001053885.1_ASM105388v1	17.4	intact	150	24	10950-28399	Mannhe_phiMHaA1_NC_008201	40.61%
H054	1				*Haemophilus parainfluenzae*	156_HINF		GCF_001054455.1_ASM105445v1	24.2	questionable	80	18	60028-84322	Shigel_SfIV_NC_022749	37.88%

TABLE S2

Strain ID	Region	Type	Supercluster	Cluster	Species	Strain	Clade	Refseq/Genbank	Region Length	Completeness	Score	# Total Proteins	Region Position	Most Common Phage	GC %
H054	2				*Haemophilus parainfluenzae*	156_HINF		GCF_001054455.1_ASM105445v1	4.6	incomplete	30	8	2-4623	Mannhe_vB_MhS_587AP2_NC_028743	42.04%
H054	3	*Lambda*	*587AP2-like*	*26*	*Haemophilus parainfluenzae*	156_HINF		GCF_001054455.1_ASM105445v1	38.5	intact	130	59	428-38973	Mannhe_vB_MhS_587AP2_NC_028743	41.05%
H055	1				*Haemophilus influenzae*	536_HINF	3	GCF_001055275.1_ASM105527v1	7.3	incomplete	60	8	835-8170	Haemop_SuMu_NC_019455	37.50%
H055	2	*P1*	*HP1-like*	*21*	*Haemophilus influenzae*	536_HINF	3	GCF_001055275.1_ASM105527v1	32.1	intact	140	43	89-32219	Haemop_HP2_NC_003315	40.71%
H055	3	*Transposable*	*BcepMu-like*	*14*	*Haemophilus influenzae*	536_HINF	3	GCF_001055275.1_ASM105527v1	31.4	intact	110	45	84180-115667	Burkho_BcepMu_NC_005882	39.96%
H055	4				*Haemophilus influenzae*	536_HINF	3	GCF_001055275.1_ASM105527v1	10.6	incomplete	30	19	137883-148561	Salmon_118970_sal3_NC_031940	37.78%
H055	5				*Haemophilus influenzae*	536_HINF	3	GCF_001055275.1_ASM105527v1	25.1	questionable	70	37	806-25987	Aggreg_S1249_NC_013597	42.04%
H055	6	*Transposable*	*SuMu-like*	*01*	*Haemophilus influenzae*	536_HINF	3	GCF_001055275.1_ASM105527v1	41.5	intact	150	53	1065-42582	Mannhe_vB_MhM_3927AP2_NC_02876	41.43%
H055	7				*Haemophilus influenzae*	536_HINF	3	GCF_001055275.1_ASM105527v1	32	incomplete	40	31	69-32100	Acinet_LZ35_NC_031117	38.20%
H055	8				*Haemophilus influenzae*	536_HINF	3	GCF_001055275.1_ASM105527v1	10.7	incomplete	40	16	69-10798	Aggreg_S1249_NC_013597	39.85%
H056	1				*Haemophilus influenzae*	615_HINF	1	GCF_001055485.1_ASM105548v1	5.9	incomplete	40	8	1508-7429	Mannhe_vB_MhM_3927AP2_NC_02876	39.38%
H056	2				*Haemophilus influenzae*	615_HINF	1	GCF_001055485.1_ASM105548v1	14.5	questionable	80	19	1129-15727	Burkho_phi6442_NC_009235	38.74%
H056	3				*Haemophilus influenzae*	615_HINF	1	GCF_001055485.1_ASM105548v1	29.1	questionable	90	42	29412-58532	Burkho_phiE255_NC_009237	40.14%
H057	1				*Haemophilus influenzae*	159_HINF	?	GCF_001055565.1_ASM105556v1	10.7	incomplete	30	17	45504-56205	Acinet_Bphi_B1251_NC_019541	34.04%
H058	1				*Haemophilus influenzae*	177_HINF	3	GCF_001055615.1_ASM105561v1	26.5	intact	121	36	329-26867	Haemop_HP2_NC_003315	40.80%
H058	2	*Transposable*	*BcepMu-like*	*14*	*Haemophilus influenzae*	177_HINF	3	GCF_001055615.1_ASM105561v1	31.9	intact	110	45	365-32359	Burkho_phiE255_NC_009237	39.97%
H058	3	*Transposable*	*SuMu-like*	*01*	*Haemophilus influenzae*	177_HINF	3	GCF_001055615.1_ASM105561v1	44.6	intact	150	49	47039-91737	Mannhe_vB_MhM_3927AP2_NC_02876	41.49%
H058	4				*Haemophilus influenzae*	177_HINF	3	GCF_001055615.1_ASM105561v1	24.5	intact	150	32	103-24684	Acinet_vB_AbaS_TRS1_NC_031098	41.45%
H058	5	*Transposable*	*BcepMu-like*	*17*	*Haemophilus influenzae*	177_HINF	3	GCF_001055615.1_ASM105561v1	36.6	intact	100	53	1-36618	Burkho_BcepMu_NC_005882	44.29%
H059	1				*Haemophilus influenzae*	40_HINF	1	GCF_001056575.1_ASM105657v1	5.9	incomplete	50	8	1524-7444	Mannhe_vB_MhM_3927AP2_NC_02876	39.20%
H059	2				*Haemophilus influenzae*	40_HINF	1	GCF_001056575.1_ASM105657v1	17.1	questionable	70	29	79644-96798	Haemop_Aaphi23_NC_004827	38.59%
H059	3				*Haemophilus influenzae*	40_HINF	1	GCF_001056575.1_ASM105657v1	20	intact	150	29	27518-47523	Mannhe_phiMHaA1_NC_008201	40.33%
H060	1	*P1*	*MHaA1-like*	*20*	*Haemophilus parainfluenzae*	841_HINF		GCF_001058575.1_ASM105857v1	31.8	intact	150	49	125637-157518	Mannhe_vB_MhM_587AP1_NC_028898	41.79%
H060	2	*Transposable*	*SuMu-like*	*02*	*Haemophilus parainfluenzae*	841_HINF		GCF_001058575.1_ASM105857v1	41.4	intact	150	50	89268-130765	Haemop_SuMu_NC_019455	41.40%
H061	1				*Haemophilus influenzae*	781_HINF		GCF_001059125.1_ASM105912v1	17.7	questionable	90	25	1-17725	Entero_c_1_NC_019706	41.33%
H061	2				*Haemophilus influenzae*	781_HINF		GCF_001059125.1_ASM105912v1	24.2	questionable	80	18	12101-36395	Shigel_SfIV_NC_022749	37.87%
H061	3				*Haemophilus influenzae*	781_HINF		GCF_001059125.1_ASM105912v1	22	incomplete	50	45	12420-34450	Mannhe_vB_MhS_1152AP2_NC_028956	38.85%
H062	1				*Haemophilus influenzae*	614_HPAR	1	GCF_001071435.1_ASM107143v1	24.2	questionable	90	41	1-24272	Mannhe_vB_MhS_587AP2_NC_028743	38.93%
H062	2				*Haemophilus influenzae*	614_HPAR	1	GCF_001071435.1_ASM107143v1	5.9	incomplete	40	7	26158-32078	Mannhe_vB_MhM_3927AP2_NC_02876	39.55%
H063	1				*Haemophilus influenzae*	1123_HINF	1	GCF_001076115.1_ASM107611v1	17.4	intact	150	24	10950-28399	Mannhe_phiMHaA1_NC_008201	40.61%
H063	2				*Haemophilus influenzae*	1123_HINF	1	GCF_001076115.1_ASM107611v1	5.9	incomplete	40	8	1417-7338	Mannhe_vB_MhM_3927AP2_NC_02876	39.40%
H063	3				*Haemophilus influenzae*	1123_HINF	1	GCF_001076115.1_ASM107611v1	9.9	incomplete	30	16	1-9917	Aggreg_S1249_NC_013597	39.34%

TABLE S2

Strain ID	Region	Type	Supercluster	Cluster	Species	Strain	Clade	Refseq/Genbank	Region Length	Completeness	Score	# Total Proteins	Region Position	Most Common Phage	GC %
H064	1				*Haemophilus influenzae*	492_HINF	2	GCF_001076835.1_ASM107683v1	24.3	incomplete	60	23	5114-29436	Brevib_Jenst_NC_028805	36.54%
H064	2				*Haemophilus influenzae*	492_HINF	2	GCF_001076835.1_ASM107683v1	17.4	incomplete	40	19	46298-63709	Bacill_BCD7_NC_019515	37.14%
H064	3				*Haemophilus influenzae*	492_HINF	2	GCF_001076835.1_ASM107683v1	27.1	questionable	90	44	74377-101556	Haemop_Aaphi23_NC_004827	38.52%
H064	4	*P1*	*MHaA1-like*	*19*	*Haemophilus influenzae*	492_HINF	2	GCF_001076835.1_ASM107683v1	28.5	intact	150	40	2846-31434	Mannhe_vB_MhM_587AP1_NC_028898	40.94%
H065	1				*Haemophilus influenzae*	HI1988	3	GCF_001184435.1_ASM118443v1	11.4	incomplete	20	13	50096-61543	Salmon_SJ46_NC_031129	43.09%
H065	2				*Haemophilus influenzae*	HI1988	3	GCF_001184435.1_ASM118443v1	12.9	incomplete	40	22	207299-220240	Mannhe_vB_MhS_1152AP2_NC_028956	36.73%
H065	3				*Haemophilus influenzae*	HI1988	3	GCF_001184435.1_ASM118443v1	26.6	incomplete	30	10	14255-40916	Haemop_SuMu_NC_019455	37.74%
H066	1				*Haemophilus influenzae*	HI2004	3	GCF_001184445.1_ASM118444v1	16.2	incomplete	40	22	32804-49016	Mannhe_vB_MhS_1152AP2_NC_028956	37.17%
H066	2				*Haemophilus influenzae*	HI2004	3	GCF_001184445.1_ASM118444v1	25.3	incomplete	30	9	39043-64354	Haemop_SuMu_NC_019455	37.79%
H067	1				*Haemophilus influenzae*	HI2007	1	GCF_001184475.1_ASM118447v1	25.2	questionable	90	41	105627-130884	Haemop_Aaphi23_NC_004827	38.40%
H067	2	*Transposable*	*BcepMu-like*	*14*	*Haemophilus influenzae*	HI2007	1	GCF_001184475.1_ASM118447v1	39.2	intact	110	56	1002-40230	Burkho_BcepMu_NC_005882	40.19%
H067	3				*Haemophilus influenzae*	HI2007	1	GCF_001184475.1_ASM118447v1	24.6	intact	100	39	14636-39319	Aggreg_S1249_NC_013597	39.82%
H067	4				*Haemophilus influenzae*	HI2007	1	GCF_001184475.1_ASM118447v1	5.9	incomplete	40	8	74483-80404	Mannhe_vB_MhM_3927AP2_NC_028766	39.41%
H067	5				*Haemophilus influenzae*	HI2007	1	GCF_001184475.1_ASM118447v1	19.3	intact	150	30	1-19308	Mannhe_phiMHaA1_NC_008201	40.35%
H068	1	*Lambda*	*Gifsy2-like*	*31*	*Haemophilus influenzae*	HI2114	1	GCF_001184485.1_ASM118448v1	29	intact	150	42	1849-30918	Entero_c_1_NC_019706	41.63%
H068	2				*Haemophilus influenzae*	HI2114	1	GCF_001184485.1_ASM118448v1	18.8	intact	150	25	102557-121430	Mannhe_phiMHaA1_NC_008201	40.49%
H068	3				*Haemophilus influenzae*	HI2114	1	GCF_001184485.1_ASM118448v1	10.9	incomplete	50	19	4440-15408	Haemop_Aaphi23_NC_004827	39.02%
H069	1				*Haemophilus influenzae*	HI2116	3	GCF_001184515.1_ASM118451v1	12.9	incomplete	40	22	17268-30218	Mannhe_vB_MhS_1152AP2_NC_028956	36.75%
H069	2				*Haemophilus influenzae*	HI2116	3	GCF_001184515.1_ASM118451v1	11.4	incomplete	20	13	49564-61011	Salmon_SJ46_NC_031129	43.09%
H069	3				*Haemophilus influenzae*	HI2116	3	GCF_001184515.1_ASM118451v1	10.1	incomplete	30	10	1207-11377	Haemop_SuMu_NC_019455	38.21%
H070	1				*Haemophilus influenzae*	HI1974	1	GCF_001184535.1_ASM118453v1	5.9	incomplete	40	7	1374-7295	Mannhe_vB_MhM_3927AP2_NC_028766	39.23%
H070	2				*Haemophilus influenzae*	HI1974	1	GCF_001184535.1_ASM118453v1	20	intact	150	28	27364-47369	Mannhe_phiMHaA1_NC_008201	40.34%
H070	3				*Haemophilus influenzae*	HI1974	1	GCF_001184535.1_ASM118453v1	17.1	questionable	70	29	4489-21685	Haemop_Aaphi23_NC_004827	38.65%
H071	1				*Haemophilus influenzae*	HI1722	1	GCF_001184545.1_ASM118454v1	5.9	incomplete	40	8	27429-33351	Mannhe_vB_MhM_3927AP2_NC_028766	39.39%
H071	2				*Haemophilus influenzae*	HI1722	1	GCF_001184545.1_ASM118454v1	12.7	incomplete	30	23	137-12878	Gordon_Twister6_NC_031052	40.36%
H071	3				*Haemophilus influenzae*	HI1722	1	GCF_001184545.1_ASM118454v1	19.4	intact	150	28	103308-122708	Mannhe_phiMHaA1_NC_008201	40.32%
H071	4	*Transposable*	*SuMu-like*	*01*	*Haemophilus influenzae*	HI1722	1	GCF_001184545.1_ASM118454v1	43.8	intact	150	48	43554-87444	Haemop_SuMu_NC_019455	41.47%
H071	5				*Haemophilus influenzae*	HI1722	1	GCF_001184545.1_ASM118454v1	27.2	intact	150	33	1452-28657	Aggreg_S1249_NC_013597	40.34%
H071	6				*Haemophilus influenzae*	HI1722	1	GCF_001184545.1_ASM118454v1	10.9	incomplete	50	17	2-10949	Haemop_Aaphi23_NC_004827	39.05%
H072	1				*Haemophilus influenzae*	HI1980	1	GCF_001184555.1_ASM118455v1	5.9	incomplete	40	8	26115-32036	Mannhe_vB_MhM_3927AP2_NC_028766	39.36%
H072	2				*Haemophilus influenzae*	HI1980	1	GCF_001184555.1_ASM118455v1	21.3	intact	150	32	267262-288604	Mannhe_phiMHaA1_NC_008201	39.92%
H073	1				*Haemophilus influenzae*	HI1426	4	GCF_001184595.1_ASM118459v1	12.4	incomplete	30	11	40602-53039	Synech_S_CAM9_NC_031922	38.18%

TABLE S2

Strain ID	Region	Type	Supercluster	Cluster	Species	Strain	Clade	Refseq/Genbank	Region Length	Completeness	Score	# Total Proteins	Region Position	Most Common Phage	GC %
H073	2				*Haemophilus influenzae*	HI1426	4	GCF_001184595.1_ASM118459v1	38.2	questionable	80	58	638696-676912	Burkho_BcepMu_NC_005882	43.70%
H073	3				*Haemophilus influenzae*	HI1426	4	GCF_001184595.1_ASM118459v1	12.3	questionable	70	19	44694-57050	Mannhe_vB_MhM_587AP1_NC_028898	40.58%
H073	4				*Haemophilus influenzae*	HI1426	4	GCF_001184595.1_ASM118459v1	36	questionable	90	47	256428-292510	Ralsto_RSP15_NC_030948	38.88%
H074	1				*Haemophilus influenzae*	HI1417	4	GCF_001184615.1_ASM118461v1	25.9	intact	150	36	285597-311513	Haemop_SuMu_NC_019455	42.37%
H074	2	*Transposable*	*BcepMu-like*	*14*	*Haemophilus influenzae*	HI1417	4	GCF_001184615.1_ASM118461v1	33.6	intact	110	47	14668-48287	Burkho_phiE255_NC_009237	39.96%
H075	1				*Haemophilus influenzae*	HI1394	1	GCF_001184635.1_ASM118463v1	5.9	incomplete	40	8	26208-32130	Mannhe_vB_MhM_3927AP2_NC_028766	39.37%
H075	2				*Haemophilus influenzae*	HI1394	1	GCF_001184635.1_ASM118463v1	21.3	intact	150	32	266260-287594	Mannhe_phiMHaA1_NC_008201	39.91%
H076	1				*Haemophilus influenzae*	HI1388	5	GCF_001184645.1_ASM118464v1	24.6	questionable	70	38	72937-97580	Haemop_Aaphi23_NC_004827	38.36%
H076	2	*P1*	*HP1-like*	*21*	*Haemophilus influenzae*	HI1388	5	GCF_001184645.1_ASM118464v1	34.5	intact	145	46	100514-135088	Haemop_HP2_NC_003315	40.00%
H076	3				*Haemophilus influenzae*	HI1388	5	GCF_001184645.1_ASM118464v1	5.9	incomplete	40	7	2289-8282	Mannhe_vB_MhM_3927AP2_NC_028766	39.64%
H077	1	*P1*	*MHaA1-like*	*19*	*Haemophilus influenzae*	HI1374	3	GCF_001184655.1_ASM118465v1	27.6	intact	150	40	76976-104607	Mannhe_vB_MhM_587AP1_NC_028898	40.49%
H077	2	*Transposable*	*BcepMu-like*	*14*	*Haemophilus influenzae*	HI1374	3	GCF_001184655.1_ASM118465v1	27.8	intact	140	44	29418-57299	Burkho_BcepMu_NC_005882	40.57%
H077	3				*Haemophilus influenzae*	HI1374	3	GCF_001184655.1_ASM118465v1	11.6	incomplete	30	19	74397-86026	Haemop_Aaphi23_NC_004827	39.03%
H078	1	*Transposable*	*BcepMu-like*	*14*	*Haemophilus influenzae*	HI1373	1	GCF_001184695.1_ASM118469v1	30.7	intact	100	44	1-30735	Burkho_phiE255_NC_009237	40.03%
H078	2				*Haemophilus influenzae*	HI1373	1	GCF_001184695.1_ASM118469v1	5.9	incomplete	40	9	23208-29128	Mannhe_vB_MhM_3927AP2_NC_028766	39.37%
H078	3				*Haemophilus influenzae*	HI1373	1	GCF_001184695.1_ASM118469v1	18.7	intact	150	29	1-18722	Mannhe_phiMHaA1_NC_008201	40.48%
H078	4	*Lambda*	*DIBBI-like*	*23*	*Haemophilus influenzae*	HI1373	1	GCF_001184695.1_ASM118469v1	39.3	intact	130	57	18190-57521	Entero_UAB_Phi20_NC_031019	40.48%
H078	5				*Haemophilus influenzae*	HI1373	1	GCF_001184695.1_ASM118469v1	17.1	questionable	70	29	44-17187	Haemop_Aaphi23_NC_004827	38.53%
H079	1				*Haemophilus influenzae*	HI1408	4	GCF_001184705.1_ASM118470v1	34.1	questionable	80	50	1-34128	Burkho_BcepMu_NC_005882	44.53%
H079	2				*Haemophilus influenzae*	HI1408	4	GCF_001184705.1_ASM118470v1	16.5	questionable	80	28	4-16591	Mannhe_vB_MhM_587AP1_NC_028898	39.15%
H079	3	*Lambda*	*Gifsy2-like*	*31*	*Haemophilus influenzae*	HI1408	4	GCF_001184705.1_ASM118470v1	28.7	intact	130	47	92777-121524	Mannhe_vB_MhS_535AP2_NC_028853	40.24%
H080	1				*Haemophilus influenzae*	C10	3	GCF_001276545.1_ASM127654v1	3.7	incomplete	30	6	2978-6701	Haemop_SuMu_NC_019455	39.80%
H080	2				*Haemophilus influenzae*	C10	3	GCF_001276545.1_ASM127654v1	24.8	questionable	80	34	1-24801	Erwini_phiEt88_NC_015295	42.07%
H080	3				*Haemophilus influenzae*	C10	3	GCF_001276545.1_ASM127654v1	21.9	incomplete	30	18	19827-41771	Salmon_118970_sal3_NC_031940	37.93%
H080	4	*Transposable*	*BcepMu-like*	*14*	*Haemophilus influenzae*	C10	3	GCF_001276545.1_ASM127654v1	30	intact	100	43	1-30024	Burkho_phiE255_NC_009237	40.11%
H080	5				*Haemophilus influenzae*	C10	3	GCF_001276545.1_ASM127654v1	15.5	questionable	90	19	62018-77604	Shigel_SfIV_NC_022749	38.03%
H081	1	*Transposable*	*SuMu-like*	*01*	*Haemophilus influenzae*	HI1413	3	GCF_001298185.1_ASM129818v1	44.7	intact	150	49	46797-91501	Mannhe_vB_MhM_3927AP2_NC_028766	41.49%
H081	2	*Transposable*	*BcepMu-like*	*17*	*Haemophilus influenzae*	HI1413	3	GCF_001298185.1_ASM129818v1	42.1	intact	110	60	87-42200	Burkho_BcepMu_NC_005882	43.27%
H081	3	*Transposable*	*BcepMu-like*	*14*	*Haemophilus influenzae*	HI1413	3	GCF_001298185.1_ASM129818v1	33.5	intact	110	47	65969-99535	Burkho_phiE255_NC_009237	39.96%
H081	4	*P1*	*HP1-like*	*21*	*Haemophilus influenzae*	HI1413	3	GCF_001298185.1_ASM129818v1	41.8	intact	150	44	106213-148027	Haemop_HP2_NC_003315	40.37%
H082	1				*Haemophilus influenzae*	HI2428	4	GCF_001298195.1_ASM129819v1	28.5	intact	150	36	311230-339764	Mannhe_vB_MhM_3927AP2_NC_028766	42.44%
H082	2				*Haemophilus influenzae*	HI2428	4	GCF_001298195.1_ASM129819v1	11.3	intact	120	17	27110-38419	Mannhe_vB_MhM_587AP1_NC_028898	39.02%

TABLE S2

Strain ID	Region	Type	Supercluster	Cluster	Species	Strain	Clade	Refseq/Genbank	Region Length	Completeness	Score	# Total Proteins	Region Position	Most Common Phage	GC %
H083	1				*Haemophilus influenzae*	HI2192	3	GCF_001298205.1_ASM129820v1	27.9	incomplete	30	9	8970-36892	Haemop_SuMu_NC_019455	36.96%
H083	2				*Haemophilus influenzae*	HI2192	3	GCF_001298205.1_ASM129820v1	16.2	incomplete	40	22	100831-117039	Mannhe_vB_MhS_1152AP2_NC_028956	37.26%
H084	1				*Haemophilus influenzae*	2842STDY5882078	1	GCF_900017145.1_14412_4_50	16.4	questionable	70	25	47-16470	Mannhe_vB_MhS_587AP2_NC_028743	39.45%
H084	2				*Haemophilus influenzae*	2842STDY5882078	1	GCF_900017145.1_14412_4_50	21.3	intact	150	34	124885-146218	Mannhe_phiMHaA1_NC_008201	40.02%
H084	3				*Haemophilus influenzae*	2842STDY5882078	1	GCF_900017145.1_14412_4_50	5.9	incomplete	40	8	122895-128816	Mannhe_vB_MhM_3927AP2_NC_02876	39.40%
H084	4	*Lambda*	*Gifsy2-like*	*31*	*Haemophilus influenzae*	2842STDY5882078	1	GCF_900017145.1_14412_4_50	35.6	intact	150	45	74639-110272	Aggreg_S1249_NC_013597	40.31%
H085	1				*Haemophilus influenzae*	2842STDY5882087	1	GCF_900017155.1_14412_4_59	14	questionable	90	16	177344-191359	Burkho_KS9_NC_013055	38.81%
H085	2				*Haemophilus influenzae*	2842STDY5882087	1	GCF_900017155.1_14412_4_59	20	intact	100	27	194003-214049	Aggreg_S1249_NC_013597	40.03%
H085	3				*Haemophilus influenzae*	2842STDY5882087	1	GCF_900017155.1_14412_4_59	25.8	incomplete	40	8	330525-356338	Mannhe_vB_MhM_3927AP2_NC_02876	38.27%
H085	4				*Haemophilus influenzae*	2842STDY5882087	1	GCF_900017155.1_14412_4_59	10.9	incomplete	60	18	3-10986	Haemop_Aaphi23_NC_004827	38.22%
H085	5				*Haemophilus influenzae*	2842STDY5882087	1	GCF_900017155.1_14412_4_59	19.3	intact	150	26	120868-140263	Mannhe_vB_MhM_587AP1_NC_028898	39.90%
H085	6				*Haemophilus influenzae*	2842STDY5882087	1	GCF_900017155.1_14412_4_59	20.3	incomplete	20	25	186836-207147	Clostr_phiCT453B_NC_029004	39.91%
H085	7	*Transposable*	*SuMu-like*	*01*	*Haemophilus influenzae*	2842STDY5882087	1	GCF_900017155.1_14412_4_59	42.2	intact	150	52	47222-89467	Haemop_SuMu_NC_019455	41.62%
H086	1				*Haemophilus influenzae*	2842STDY5882032	2	GCF_900020195.1_14412_4_4	15.3	questionable	70	20	127845-143185	Shigel_SfIV_NC_022749	40.08%
H086	2				*Haemophilus influenzae*	2842STDY5882032	2	GCF_900020195.1_14412_4_4	11.7	incomplete	30	17	151809-163603	Bacill_BCD7_NC_019515	37.50%
H086	3				*Haemophilus influenzae*	2842STDY5882032	2	GCF_900020195.1_14412_4_4	10	incomplete	50	15	172752-182845	Mannhe_vB_MhS_587AP2_NC_028743	38.78%
H086	4				*Haemophilus influenzae*	2842STDY5882032	2	GCF_900020195.1_14412_4_4	24.7	intact	100	39	3-24787	Aggreg_S1249_NC_013597	40.86%
H086	5	*Lambda*	*Gifsy2-like*	*31*	*Haemophilus influenzae*	2842STDY5882032	2	GCF_900020195.1_14412_4_4	31.5	intact	150	40	60735-92240	Mannhe_vB_MhS_535AP2_NC_028853	41.27%
H086	6				*Haemophilus influenzae*	2842STDY5882032	2	GCF_900020195.1_14412_4_4	10.6	intact	110	12	622-11292	Mannhe_vB_MhM_3927AP2_NC_02876	42.32%
H087	1				*Haemophilus influenzae*	2842STDY5882021	1	GCF_900022475.1_14412_3_88	21.3	intact	150	31	102432-123805	Mannhe_phiMHaA1_NC_008201	39.49%
H087	2				*Haemophilus influenzae*	2842STDY5882021	1	GCF_900022475.1_14412_3_88	24.9	questionable	80	38	229100-254067	Haemop_Aaphi23_NC_004827	38.41%
H087	3				*Haemophilus influenzae*	2842STDY5882021	1	GCF_900022475.1_14412_3_88	5.9	incomplete	40	8	373242-379164	Mannhe_vB_MhM_3927AP2_NC_02876	39.42%
H087	4	*Lambda*	*DIBBI-like*	*23*	*Haemophilus influenzae*	2842STDY5882021	1	GCF_900022475.1_14412_3_88	61	intact	150	73	142529-203567	Pseudo_phi2_NC_030931	39.63%
H088	1				*Haemophilus influenzae*	2842STDY5882040	4	GCF_900022485.1_14412_4_12	11.8	intact	100	16	351058-362916	Mannhe_vB_MhM_587AP1_NC_028898	39.42%
H088	2				*Haemophilus influenzae*	2842STDY5882040	4	GCF_900022485.1_14412_4_12	22.4	intact	140	30	147-22599	Haemop_SuMu_NC_019455	41.39%
H088	3				*Haemophilus influenzae*	2842STDY5882040	4	GCF_900022485.1_14412_4_12	19.4	incomplete	30	12	270901-290352	Gordon_CaptainKirk2_NC_031072	38.34%
H088	4				*Haemophilus influenzae*	2842STDY5882040	4	GCF_900022485.1_14412_4_12	16.9	incomplete	40	31	93-17089	Mannhe_vB_MhM_3927AP2_NC_02876	42.05%
H088	5				*Haemophilus influenzae*	2842STDY5882040	4	GCF_900022485.1_14412_4_12	18.2	questionable	90	22	137407-155705	Haemop_SuMu_NC_019455	44.59%
H089	1				*Haemophilus influenzae*	2842STDY5882085	2	GCF_900022495.1_14412_4_57	15.3	questionable	70	20	138410-153739	Shigel_SfIV_NC_022749	40.08%
H089	2				*Haemophilus influenzae*	2842STDY5882085	2	GCF_900022495.1_14412_4_57	11.7	incomplete	30	17	169909-181703	Bacill_BCD7_NC_019515	37.49%
H089	3				*Haemophilus influenzae*	2842STDY5882085	2	GCF_900022495.1_14412_4_57	10	incomplete	50	15	172715-182808	Mannhe_vB_MhS_587AP2_NC_028743	38.76%
H089	4				*Haemophilus influenzae*	2842STDY5882085	2	GCF_900022495.1_14412_4_57	24.7	intact	100	39	82976-107763	Pseudo_JBD44_NC_030929	40.86%

TABLE S2

Strain ID	Region	Type	Supercluster	Cluster	Species	Strain	Clade	Refseq/Genbank	Region Length	Completeness	Score	# Total Proteins	Region Position	Most Common Phage	GC %
H089	5	*Lambda*	*Gifsy2-like*	*31*	*Haemophilus influenzae*	2842STDY5882085	2	GCF_900022495.1_14412_4_57	30.7	intact	150	40	50647-81414	Mannhe_vB_MhS_535AP2_NC_028853	41.21%
H089	6				*Haemophilus influenzae*	2842STDY5882085	2	GCF_900022495.1_14412_4_57	10.6	intact	110	12	622-11292	Mannhe_vB_MhM_3927AP2_NC_028766	42.31%
H090	1				*Haemophilus influenzae*	2842STDY5882019	4	GCF_900022505.1_14412_3_86	12.5	incomplete	30	11	154821-167357	Synech_S_CAM9_NC_031922	38.51%
H090	2				*Haemophilus influenzae*	2842STDY5882019	4	GCF_900022505.1_14412_3_86	31.4	intact	150	29	413861-445280	Mannhe_vB_MhM_3927AP2_NC_028766	39.78%
H090	3				*Haemophilus influenzae*	2842STDY5882019	4	GCF_900022505.1_14412_3_86	23.8	intact	130	34	339233-363070	Mannhe_vB_MhM_587AP1_NC_028898	38.48%
H090	4				*Haemophilus influenzae*	2842STDY5882019	4	GCF_900022505.1_14412_3_86	16.9	incomplete	40	31	95-17067	Mannhe_vB_MhM_3927AP2_NC_028766	42.07%
H090	5				*Haemophilus influenzae*	2842STDY5882019	4	GCF_900022505.1_14412_3_86	17.9	questionable	90	21	133692-151646	Haemop_SuMu_NC_019455	43.89%
H091	1				*Haemophilus influenzae*	2842STDY5882056	2	GCF_900022515.1_14412_4_28	15.4	questionable	70	20	163962-179385	Shigel_SfIV_NC_022749	40.00%
H091	2				*Haemophilus influenzae*	2842STDY5882056	2	GCF_900022515.1_14412_4_28	11.7	incomplete	30	17	169138-180932	Bacill_BCD7_NC_019515	37.50%
H091	3				*Haemophilus influenzae*	2842STDY5882056	2	GCF_900022515.1_14412_4_28	24.7	intact	100	39	84228-109012	Pseudo_JBD44_NC_030929	40.85%
H091	4	*Lambda*	*Gifsy2-like*	*31*	*Haemophilus influenzae*	2842STDY5882056	2	GCF_900022515.1_14412_4_28	39.7	intact	150	55	401-40144	Mannhe_vB_MhS_535AP2_NC_028853	40.75%
H091	5				*Haemophilus influenzae*	2842STDY5882056	2	GCF_900022515.1_14412_4_28	10.6	intact	110	12	439-11109	Mannhe_vB_MhM_3927AP2_NC_028766	42.32%
H092	1				*Haemophilus influenzae*	2842STDY5882058	2	GCF_900022525.1_14412_4_30	11.7	incomplete	30	17	169992-181786	Bacill_BCD7_NC_019515	37.50%
H092	2				*Haemophilus influenzae*	2842STDY5882058	2	GCF_900022525.1_14412_4_30	15.4	questionable	70	20	138429-153852	Shigel_SfIV_NC_022749	40.00%
H092	3				*Haemophilus influenzae*	2842STDY5882058	2	GCF_900022525.1_14412_4_30	19.2	incomplete	50	19	166316-185587	Mannhe_vB_MhS_587AP2_NC_028743	38.37%
H092	4	*Lambda*	*Aaphi23-like*	*24*	*Haemophilus influenzae*	2842STDY5882058	2	GCF_900022525.1_14412_4_30	38.3	intact	150	54	439-38822	Aggreg_S1249_NC_013597	41.14%
H093	1	*Lambda*	*DIBBI-like*	*23*	*Haemophilus influenzae*	2842STDY5882036	1	GCF_900026975.1_14412_4_8	50.7	intact	130	71	218709-269473	Aggreg_S1249_NC_013597	40.39%
H093	2				*Haemophilus influenzae*	2842STDY5882036	1	GCF_900026975.1_14412_4_8	20.7	intact	150	28	104976-125705	Mannhe_phiMHaA1_NC_008201	40.18%
H093	3				*Haemophilus influenzae*	2842STDY5882036	1	GCF_900026975.1_14412_4_8	17.1	questionable	70	27	235269-252457	Haemop_Aaphi23_NC_004827	37.80%
H093	4				*Haemophilus influenzae*	2842STDY5882036	1	GCF_900026975.1_14412_4_8	5.9	incomplete	40	9	120950-126870	Mannhe_vB_MhM_3927AP2_NC_028766	39.39%
H094	1				*Haemophilus influenzae*	2842STDY5882049	2	GCF_900026985.1_14412_4_21	11.7	incomplete	30	16	126013-137807	Bacill_BCD7_NC_019515	37.51%
H094	2				*Haemophilus influenzae*	2842STDY5882049	2	GCF_900026985.1_14412_4_21	15.4	questionable	70	20	138388-153811	Shigel_SfIV_NC_022749	40.00%
H094	3	*Lambda*	*Aaphi23-like*	*24*	*Haemophilus influenzae*	2842STDY5882049	2	GCF_900026985.1_14412_4_21	25.6	intact	103	41	1-25613	Aggreg_S1249_NC_013597	40.82%
H094	4				*Haemophilus influenzae*	2842STDY5882049	2	GCF_900026985.1_14412_4_21	30.7	intact	150	39	3-30800	Mannhe_vB_MhS_535AP2_NC_028853	41.22%
H094	5				*Haemophilus influenzae*	2842STDY5882049	2	GCF_900026985.1_14412_4_21	11.2	intact	120	14	439-11735	Mannhe_vB_MhM_3927AP2_NC_028766	41.90%
H095	1				*Haemophilus influenzae*	2842STDY5882055	2	GCF_900026995.1_14412_4_27	11.7	incomplete	30	17	169956-181750	Bacill_BCD7_NC_019515	37.50%
H095	2				*Haemophilus influenzae*	2842STDY5882055	2	GCF_900026995.1_14412_4_27	24.7	intact	100	39	391859-416643	Pseudo_JBD44_NC_030929	40.85%
H095	3				*Haemophilus influenzae*	2842STDY5882055	2	GCF_900026995.1_14412_4_27	15.4	questionable	70	20	163125-178548	Shigel_SfIV_NC_022749	40.00%
H095	4	*Lambda*	*Gifsy2-like*	*31*	*Haemophilus influenzae*	2842STDY5882055	2	GCF_900026995.1_14412_4_27	40.1	intact	150	58	60599-100737	Mannhe_vB_MhS_587AP2_NC_028743	40.75%
H095	5				*Haemophilus influenzae*	2842STDY5882055	2	GCF_900026995.1_14412_4_27	10.4	incomplete	30	16	27636-38037	Aggreg_S1249_NC_013597	37.08%
H095	6				*Haemophilus influenzae*	2842STDY5882055	2	GCF_900026995.1_14412_4_27	10.6	intact	110	12	439-11109	Mannhe_vB_MhM_3927AP2_NC_028766	42.32%
H096	1				*Haemophilus influenzae*	2842STDY5882046	1	GCF_900027005.1_14412_4_18	29.1	questionable	90	43	534963-564157	Burkho_phiE255_NC_009237	40.10%

TABLE S2

Strain ID	Region	Type	Supercluster	Cluster	Species	Strain	Clade	Refseq/Genbank	Region Length	Completeness	Score	# Total Proteins	Region Position	Most Common Phage	GC %
H096	2				*Haemophilus influenzae*	2842STDY5882046	1	GCF_900027005.1_14412_4_18	19.2	incomplete	20	22	424-19696	Mannhe_vB_MhS_587AP2_NC_028743	38.68%
H096	3				*Haemophilus influenzae*	2842STDY5882046	1	GCF_900027005.1_14412_4_18	15.2	questionable	90	20	177776-193027	Entero_c_1_NC_019706	42.06%
H096	4				*Haemophilus influenzae*	2842STDY5882046	1	GCF_900027005.1_14412_4_18	5.9	incomplete	40	8	27834-33755	Mannhe_vB_MhM_3927AP2_NC_028766	39.41%
H096	5				*Haemophilus influenzae*	2842STDY5882046	1	GCF_900027005.1_14412_4_18	12.9	incomplete	60	21	3-12972	Acinet_LZ35_NC_031117	38.34%
H096	6				*Haemophilus influenzae*	2842STDY5882046	1	GCF_900027005.1_14412_4_18	17.1	intact	150	27	102741-119911	Mannhe_vB_MhM_587AP1_NC_028898	39.92%
H097	1				*Haemophilus influenzae*	2842STDY5882079	1	GCF_900027015.1_14412_4_51	16.4	questionable	70	25	47-16470	Mannhe_vB_MhS_587AP2_NC_028743	39.44%
H097	2				*Haemophilus influenzae*	2842STDY5882079	1	GCF_900027015.1_14412_4_51	21.3	intact	150	34	124885-146218	Mannhe_phiMHaA1_NC_008201	40.02%
H097	3				*Haemophilus influenzae*	2842STDY5882079	1	GCF_900027015.1_14412_4_51	21.8	incomplete	40	18	259-22062	Salmon_SJ46_NC_031129	39.06%
H097	4	*Lambda*	*Gifsy2-like*	*31*	*Haemophilus influenzae*	2842STDY5882079	1	GCF_900027015.1_14412_4_51	35.6	intact	150	45	47-35680	Aggreg_S1249_NC_013597	40.31%
H097	5				*Haemophilus influenzae*	2842STDY5882079	1	GCF_900027015.1_14412_4_51	5.9	incomplete	40	8	122900-128821	Mannhe_vB_MhM_3927AP2_NC_028766	39.40%
H098	1				*Haemophilus influenzae*	2842STDY5882034	1	GCF_900029195.1_14412_4_6	21.3	intact	150	33	103779-125117	Mannhe_phiMHaA1_NC_008201	40.03%
H098	2				*Haemophilus influenzae*	2842STDY5882034	1	GCF_900029195.1_14412_4_6	24.3	questionable	80	39	230813-255118	Haemop_Aaphi23_NC_004827	38.23%
H098	3				*Haemophilus influenzae*	2842STDY5882034	1	GCF_900029195.1_14412_4_6	5.9	incomplete	40	8	371612-377533	Mannhe_vB_MhM_3927AP2_NC_028766	39.40%
H098	4	*Lambda*	*Gifsy2-like*	*31*	*Haemophilus influenzae*	2842STDY5882034	1	GCF_900029195.1_14412_4_6	30.5	intact	150	44	91252-121757	Pseudo_phi2_NC_030931	40.75%
H098	5	*P1*	*HP1-like*	*21*	*Haemophilus influenzae*	2842STDY5882034	1	GCF_900029195.1_14412_4_6	40.3	intact	133	41	89020-129371	Haemop_HP2_NC_003315	39.75%
H099	1	*Lambda*	*Gifsy2-like*	*31*	*Haemophilus influenzae*	2842STDY5882088	1	GCF_900029205.1_14412_4_60	41.8	intact	120	48	192993-234855	Pseudo_MD8_NC_031091	39.33%
H099	2				*Haemophilus influenzae*	2842STDY5882088	1	GCF_900029205.1_14412_4_60	19.8	intact	150	25	103061-122956	Mannhe_phiMHaA1_NC_008201	40.40%
H099	3				*Haemophilus influenzae*	2842STDY5882088	1	GCF_900029205.1_14412_4_60	10.9	questionable	70	20	232338-243321	Haemop_Aaphi23_NC_004827	39.01%
H099	4				*Haemophilus influenzae*	2842STDY5882088	1	GCF_900029205.1_14412_4_60	5.9	incomplete	40	8	21756-27677	Mannhe_vB_MhM_3927AP2_NC_028766	39.36%
H099	5	*Transposable*	*SuMu-like*	*01*	*Haemophilus influenzae*	2842STDY5882088	1	GCF_900029205.1_14412_4_60	42.2	intact	150	52	47287-89532	Haemop_SuMu_NC_019455	41.62%
H100	1	*Transposable*	*SuMu-like*	*01*	*Haemophilus influenzae*	2842STDY5882080	3	GCF_900029335.1_14412_4_52	33.7	intact	150	47	509365-543153	Haemop_SuMu_NC_019455	42.34%
H100	2				*Haemophilus influenzae*	2842STDY5882080	3	GCF_900029335.1_14412_4_52	15.8	questionable	90	34	206079-221899	Aggreg_S1249_NC_013597	38.12%
H100	3	*P1*	*MHaA1-like*	*19*	*Haemophilus influenzae*	2842STDY5882080	3	GCF_900029335.1_14412_4_52	32.9	intact	150	49	326352-359261	Mannhe_vB_MhM_587AP1_NC_028898	40.85%
H100	4	*P1*	*HP1-like*	*21*	*Haemophilus influenzae*	2842STDY5882080	3	GCF_900029335.1_14412_4_52	37.2	intact	113	41	94352-131614	Haemop_HP1_NC_001697	39.55%
H101	1				*Haemophilus influenzae*	2842STDY5882050	2	GCF_900029875.1_14412_4_22	11.7	incomplete	30	16	126012-137806	Bacill_BCD7_NC_019515	37.51%
H101	2				*Haemophilus influenzae*	2842STDY5882050	2	GCF_900029875.1_14412_4_22	15.3	questionable	70	20	127838-143167	Shigel_SfIV_NC_022749	40.08%
H101	3				*Haemophilus influenzae*	2842STDY5882050	2	GCF_900029875.1_14412_4_22	24.7	intact	100	39	3-24787	Aggreg_S1249_NC_013597	40.86%
H101	4	*Lambda*	*Gifsy2-like*	*31*	*Haemophilus influenzae*	2842STDY5882050	2	GCF_900029875.1_14412_4_22	31.6	intact	150	42	60605-92230	Mannhe_vB_MhS_535AP2_NC_028853	41.18%
H101	5				*Haemophilus influenzae*	2842STDY5882050	2	GCF_900029875.1_14412_4_22	10.6	intact	110	12	622-11292	Mannhe_vB_MhM_3927AP2_NC_028766	42.31%
H102	1	*Lambda*	*Gifsy2-like*	*31*	*Haemophilus influenzae*	2842STDY5882035	1	GCF_900031505.1_14412_4_7	38.9	intact	150	44	220189-259110	Pseudo_MD8_NC_031091	40.16%
H102	2				*Haemophilus influenzae*	2842STDY5882035	1	GCF_900031505.1_14412_4_7	21.3	intact	150	33	102173-123511	Mannhe_phiMHaA1_NC_008201	40.03%
H102	3				*Haemophilus influenzae*	2842STDY5882035	1	GCF_900031505.1_14412_4_7	24.2	questionable	80	38	229217-253505	Haemop_Aaphi23_NC_004827	37.79%

TABLE S2

Strain ID	Region	Type	Supercluster	Cluster	Species	Strain	Clade	Refseq/Genbank	Region Length	Completeness	Score	# Total Proteins	Region Position	Most Common Phage	GC %
H102	4				*Haemophilus influenzae*	2842STDY5882035	1	GCF_900031505.1_14412_4_7	5.9	incomplete	40	8	370747-376668	Mannhe_vB_MhM_3927AP2_NC_028766	39.40%
H102	5	*P1*	*HP1-like*	*21*	*Haemophilus influenzae*	2842STDY5882035	1	GCF_900031505.1_14412_4_7	31.1	intact	133	41	40375-71544	Haemop_HP2_NC_003315	40.40%
H103	1	*Lambda*	*DIBBI-like*	*23*	*Haemophilus influenzae*	2842STDY5882037	1	GCF_900032115.1_14412_4_9	50.8	intact	130	70	220194-271005	Aggreg_S1249_NC_013597	40.14%
H103	2				*Haemophilus influenzae*	2842STDY5882037	1	GCF_900032115.1_14412_4_9	20.6	intact	150	27	104983-125653	Mannhe_vB_MhM_587AP1_NC_028898	39.42%
H103	3				*Haemophilus influenzae*	2842STDY5882037	1	GCF_900032115.1_14412_4_9	17.1	questionable	70	28	235217-252360	Haemop_Aaphi23_NC_004827	38.54%
H103	4				*Haemophilus influenzae*	2842STDY5882037	1	GCF_900032115.1_14412_4_9	5.9	incomplete	40	9	23276-29196	Mannhe_vB_MhM_3927AP2_NC_028766	39.39%
H104	1				*Haemophilus influenzae*	2842STDY5882018	4	GCF_900034905.1_14412_3_85	16.9	incomplete	40	31	507209-524181	Mannhe_vB_MhM_3927AP2_NC_028766	42.07%
H104	2				*Haemophilus influenzae*	2842STDY5882018	4	GCF_900034905.1_14412_3_85	22.4	intact	140	27	187-22651	Haemop_SuMu_NC_019455	40.78%
H104	3				*Haemophilus influenzae*	2842STDY5882018	4	GCF_900034905.1_14412_3_85	19.4	incomplete	30	11	270966-290417	Gordon_CaptainKirk2_NC_031072	38.34%
H104	4				*Haemophilus influenzae*	2842STDY5882018	4	GCF_900034905.1_14412_3_85	17.9	questionable	90	22	133328-151269	Haemop_SuMu_NC_019455	44.67%
H105	1	*Transposable*	*BcepMu-like*	*14*	*Haemophilus influenzae*	2842STDY5882045	1	GCF_900034915.1_14412_4_17	30.6	intact	100	44	1-30615	Burkho_phiE255_NC_009237	40.07%
H105	2				*Haemophilus influenzae*	2842STDY5882045	1	GCF_900034915.1_14412_4_17	37.9	questionable	90	47	103399-141346	Aggreg_S1249_NC_013597	38.00%
H105	3				*Haemophilus influenzae*	2842STDY5882045	1	GCF_900034915.1_14412_4_17	15.2	questionable	90	20	177776-193027	Entero_c_1_NC_019706	42.06%
H105	4				*Haemophilus influenzae*	2842STDY5882045	1	GCF_900034915.1_14412_4_17	5.9	incomplete	40	8	28589-34510	Mannhe_vB_MhM_3927AP2_NC_028766	39.41%
H105	5				*Haemophilus influenzae*	2842STDY5882045	1	GCF_900034915.1_14412_4_17	17.1	intact	150	27	102749-119919	Mannhe_vB_MhM_587AP1_NC_028898	39.92%
H106	1	*Transposable*	*BcepMu-like*	*17*	*Haemophilus influenzae*	2842STDY5882051	3	GCF_900034925.1_14412_4_23	43.9	intact	100	62	137015-180990	Burkho_BcepMu_NC_005882	43.67%
H106	2	*Lambda*	*DIBBI-like*	*23*	*Haemophilus influenzae*	2842STDY5882051	3	GCF_900034925.1_14412_4_23	62.5	intact	150	73	3-62582	Acinet_LZ35_NC_031117	39.84%
H106	3				*Haemophilus influenzae*	2842STDY5882051	3	GCF_900034925.1_14412_4_23	15.5	incomplete	60	30	38248-53822	Aggreg_S1249_NC_013597	39.00%
H106	4	*Transposable*	*BcepMu-like*	*14*	*Haemophilus influenzae*	2842STDY5882051	3	GCF_900034925.1_14412_4_23	31.9	intact	110	45	91717-123709	Burkho_phiE255_NC_009237	39.94%
H106	5	*Transposable*	*Mu-like*	*06*	*Haemophilus influenzae*	2842STDY5882051	3	GCF_900034925.1_14412_4_23	33.2	intact	150	50	26533-59768	Escher_D108_NC_013594	43.73%
H106	6	*Transposable*	*SuMu-like*	*01*	*Haemophilus influenzae*	2842STDY5882051	3	GCF_900034925.1_14412_4_23	34.9	intact	150	50	387-35352	Mannhe_vB_MhM_3927AP2_NC_028766	42.22%
H107	1				*Haemophilus influenzae*	2842STDY5882060	1	GCF_900034935.1_14412_4_32	5.9	incomplete	40	8	29165-35087	Mannhe_vB_MhM_3927AP2_NC_028766	39.30%
H107	2				*Haemophilus influenzae*	2842STDY5882060	1	GCF_900034935.1_14412_4_32	21.3	intact	150	32	270272-291606	Mannhe_phiMHaA1_NC_008201	39.92%
H108	1				*Haemophilus influenzae*	2842STDY5882086	2	GCF_900034945.1_14412_4_58	15.3	questionable	70	20	127839-143168	Shigel_SfIV_NC_022749	40.08%
H108	2				*Haemophilus influenzae*	2842STDY5882086	2	GCF_900034945.1_14412_4_58	11.7	incomplete	30	16	126020-137814	Bacill_BCD7_NC_019515	37.49%
H108	3	*Lambda*	*Gifsy2-like*	*31*	*Haemophilus influenzae*	2842STDY5882086	2	GCF_900034945.1_14412_4_58	64.3	intact	150	70	48125-112469	Mannhe_vB_MhS_587AP2_NC_028743	39.42%
H108	4				*Haemophilus influenzae*	2842STDY5882086	2	GCF_900034945.1_14412_4_58	24.7	intact	100	39	87048-111832	Pseudo_JBD44_NC_030929	40.85%
H108	5				*Haemophilus influenzae*	2842STDY5882086	2	GCF_900034945.1_14412_4_58	10.6	intact	110	12	439-11109	Mannhe_vB_MhM_3927AP2_NC_028766	42.31%
H109	1				*Haemophilus influenzae*	2842STDY5882041	4	GCF_900035255.1_14412_4_13	16.9	incomplete	40	32	93-17089	Mannhe_vB_MhM_3927AP2_NC_028766	42.05%
H109	2				*Haemophilus influenzae*	2842STDY5882041	4	GCF_900035255.1_14412_4_13	12.4	incomplete	30	11	154926-167350	Synech_S_CAM9_NC_031922	38.41%
H109	3				*Haemophilus influenzae*	2842STDY5882041	4	GCF_900035255.1_14412_4_13	32.4	intact	150	30	413841-446264	Mannhe_vB_MhM_3927AP2_NC_028766	39.81%
H109	4				*Haemophilus influenzae*	2842STDY5882041	4	GCF_900035255.1_14412_4_13	17.9	intact	110	22	2-17944	Haemop_SuMu_NC_019455	44.66%

TABLE S2

Strain ID	Region	Type	Supercluster	Cluster	Species	Strain	Clade	Refseq/Genbank	Region Length	Completeness	Score	# Total Proteins	Region Position	Most Common Phage	GC %
H110	1				*Haemophilus influenzae*	2842STDY5882048	2	GCF_900035265.1_14412_4_20	15.4	questionable	70	20	208660-224083	Shigel_SfIV_NC_022749	40.00%
H110	2				*Haemophilus influenzae*	2842STDY5882048	2	GCF_900035265.1_14412_4_20	11.7	incomplete	30	16	125971-137765	Bacill_BCD7_NC_019515	37.50%
H110	3				*Haemophilus influenzae*	2842STDY5882048	2	GCF_900035265.1_14412_4_20	19.2	incomplete	50	19	519-19790	Mannhe_vB_MhS_587AP2_NC_028743	38.38%
H110	4	*Lambda*	*Aaphi23-like*	*24*	*Haemophilus influenzae*	2842STDY5882048	2	GCF_900035265.1_14412_4_20	27.9	intact	103	44	84244-112190	Aggreg_S1249_NC_013597	40.81%
H110	5				*Haemophilus influenzae*	2842STDY5882048	2	GCF_900035265.1_14412_4_20	10.6	intact	110	12	439-11109	Mannhe_vB_MhM_3927AP2_NC_02876	42.32%
H111	1	*Lambda*	*DIBBI-like*	*23*	*Haemophilus influenzae*	2842STDY5882052	3	GCF_900035275.1_14412_4_24	43.4	intact	140	65	467-43866	Erwini_phiEt88_NC_015295	40.45%
H111	2				*Haemophilus influenzae*	2842STDY5882052	3	GCF_900035275.1_14412_4_24	36	questionable	90	53	28-36122	Burkho_BcepMu_NC_005882	44.43%
H111	3	*Transposable*	*BcepMu-like*	*14*	*Haemophilus influenzae*	2842STDY5882052	3	GCF_900035275.1_14412_4_24	33.1	intact	110	46	15962-49151	Burkho_phiE255_NC_009237	39.95%
H111	4				*Haemophilus influenzae*	2842STDY5882052	3	GCF_900035275.1_14412_4_24	18.1	incomplete	60	31	64653-82846	Aggreg_S1249_NC_013597	38.90%
H111	5	*Transposable*	*Mu-like*	*06*	*Haemophilus influenzae*	2842STDY5882052	3	GCF_900035275.1_14412_4_24	33.2	intact	150	50	42-33282	Escher_D108_NC_013594	43.75%
H111	6	*Transposable*	*SuMu-like*	*01*	*Haemophilus influenzae*	2842STDY5882052	3	GCF_900035275.1_14412_4_24	34.9	intact	150	54	387-35358	Mannhe_vB_MhM_3927AP2_NC_02876	41.90%
H112	1				*Haemophilus influenzae*	2842STDY5882062	1	GCF_900035285.1_14412_4_34	5.9	incomplete	40	8	24911-30832	Mannhe_vB_MhM_3927AP2_NC_02876	39.41%
H112	2	*Lambda*	*Aaphi23-like*	*24*	*Haemophilus influenzae*	2842STDY5882062	1	GCF_900035285.1_14412_4_34	35.9	intact	100	41	134015-169976	Haemop_Aaphi23_NC_004827	38.02%
H112	3				*Haemophilus influenzae*	2842STDY5882062	1	GCF_900035285.1_14412_4_34	23.8	intact	100	37	295637-319478	Aggreg_S1249_NC_013597	39.89%
H112	4	*Transposable*	*BcepMu-like*	*14*	*Haemophilus influenzae*	2842STDY5882062	1	GCF_900035285.1_14412_4_34	39.2	intact	110	55	1080-40308	Burkho_BcepMu_NC_005882	40.19%
H112	5				*Haemophilus influenzae*	2842STDY5882062	1	GCF_900035285.1_14412_4_34	19.3	intact	150	31	1-19336	Mannhe_phiMHaA1_NC_008201	40.35%
H113	1	*Transposable*	*SuMu-like*	*01*	*Haemophilus influenzae*	2842STDY5882081	3	GCF_900035295.1_14412_4_53	31.6	intact	150	46	509358-541031	Haemop_SuMu_NC_019455	42.51%
H113	2	*P1*	*MHaA1-like*	*19*	*Haemophilus influenzae*	2842STDY5882081	3	GCF_900035295.1_14412_4_53	32.7	intact	150	47	177708-210448	Mannhe_vB_MhM_587AP1_NC_028898	40.88%
H113	3				*Haemophilus influenzae*	2842STDY5882081	3	GCF_900035295.1_14412_4_53	18.4	questionable	90	35	312265-330737	Acinet_vB_AbaS_TRS1_NC_031098	38.06%
H113	4				*Haemophilus influenzae*	2842STDY5882081	3	GCF_900035295.1_14412_4_53	5.9	incomplete	40	8	447717-453638	Mannhe_vB_MhM_3927AP2_NC_02876	39.29%
H113	5	*P1*	*HP1-like*	*21*	*Haemophilus influenzae*	2842STDY5882081	3	GCF_900035295.1_14412_4_53	27.7	intact	113	40	1466-29239	Haemop_HP1_NC_001697	39.96%
H114	1				*Haemophilus influenzae*	2842STDY5882059	1	GCF_900035525.1_14412_4_31	5.9	incomplete	40	8	29172-35094	Mannhe_vB_MhM_3927AP2_NC_02876	39.30%
H114	2				*Haemophilus influenzae*	2842STDY5882059	1	GCF_900035525.1_14412_4_31	21.3	intact	150	32	270298-291632	Mannhe_phiMHaA1_NC_008201	39.92%
H115	1				*Haemophilus influenzae*	2842STDY5882076	4	GCF_900035535.1_14412_4_48	28.5	intact	150	38	431349-459871	Mannhe_vB_MhM_3927AP2_NC_02876	42.44%
H115	2				*Haemophilus influenzae*	2842STDY5882076	4	GCF_900035535.1_14412_4_48	8.8	intact	120	16	47674-56532	Mannhe_vB_MhM_587AP1_NC_028898	39.61%
H116	1				*Haemophilus influenzae*	2842STDY5882057	2	GCF_900036645.1_14412_4_29	15.4	questionable	70	20	208333-223756	Shigel_SfIV_NC_022749	40.00%
H116	2				*Haemophilus influenzae*	2842STDY5882057	2	GCF_900036645.1_14412_4_29	11.7	incomplete	30	16	125945-137739	Bacill_BCD7_NC_019515	37.50%
H116	3				*Haemophilus influenzae*	2842STDY5882057	2	GCF_900036645.1_14412_4_29	19.2	incomplete	50	19	166313-185584	Mannhe_vB_MhS_587AP2_NC_028743	38.37%
H116	4	*Lambda*	*Aaphi23-like*	*24*	*Haemophilus influenzae*	2842STDY5882057	2	GCF_900036645.1_14412_4_29	27.5	intact	103	44	486-27997	Aggreg_S1249_NC_013597	40.82%
H116	5				*Haemophilus influenzae*	2842STDY5882057	2	GCF_900036645.1_14412_4_29	11.2	intact	120	14	439-11735	Mannhe_vB_MhM_3927AP2_NC_02876	41.90%
H117	1				*Haemophilus influenzae*	2842STDY5882065	1	GCF_900037345.1_14412_4_37	17.1	questionable	70	29	111313-128509	Haemop_Aaphi23_NC_004827	38.65%
H117	2				*Haemophilus influenzae*	2842STDY5882065	1	GCF_900037345.1_14412_4_37	15.5	intact	140	23	245313-260851	Mannhe_vB_MhM_587AP1_NC_028898	39.84%

TABLE S2

Strain ID	Region	Type	Supercluster	Cluster	Species	Strain	Clade	Refseq/Genbank	Region Length	Completeness	Score	# Total Proteins	Region Position	Most Common Phage	GC %
H117	3				*Haemophilus influenzae*	2842STDY5882065	1	GCF_900037345.1_14412_4_37	42	intact	150	38	11491-53514	Mannhe_vB_MhM_587AP1_NC_028898	38.74%
H118	1	*Transposable*	*BcepMu-like*	*14*	*Haemophilus influenzae*	2842STDY5882061	1	GCF_900038065.1_14412_4_33	40.3	intact	110	56	1-40351	Burkho_BcepMu_NC_005882	40.16%
H118	2				*Haemophilus influenzae*	2842STDY5882061	1	GCF_900038065.1_14412_4_33	5.9	incomplete	40	8	24904-30825	Mannhe_vB_MhM_3927AP2_NC_02876	39.41%
H118	3	*Lambda*	*Aaphi23-like*	*24*	*Haemophilus influenzae*	2842STDY5882061	1	GCF_900038065.1_14412_4_33	35.9	intact	100	41	134012-169973	Haemop_Aaphi23_NC_004827	38.02%
H118	4	*P1*	*MHaA1-like*	*19*	*Haemophilus influenzae*	2842STDY5882061	1	GCF_900038065.1_14412_4_33	44.8	intact	150	64	14703-59522	Mannhe_phiMHaA1_NC_008201	39.97%
H119	1				*Haemophilus influenzae*	2842STDY5882064	1	GCF_900039575.1_14412_4_36	17.1	questionable	70	29	111307-128503	Haemop_Aaphi23_NC_004827	38.65%
H119	2				*Haemophilus influenzae*	2842STDY5882064	1	GCF_900039575.1_14412_4_36	26.2	intact	150	36	1-26285	Mannhe_vB_MhM_587AP1_NC_028898	40.56%
H119	3				*Haemophilus influenzae*	2842STDY5882064	1	GCF_900039575.1_14412_4_36	13	intact	150	20	209-13245	Mannhe_vB_MhM_587AP1_NC_028898	40.39%
H120	1				*Haemophilus influenzae*	2842STDY5882047	2	GCF_900041425.1_14412_4_19	15.4	questionable	70	20	218476-233899	Shigel_SfIV_NC_022749	40.00%
H120	2				*Haemophilus influenzae*	2842STDY5882047	2	GCF_900041425.1_14412_4_19	11.7	incomplete	30	17	151824-163618	Bacill_BCD7_NC_019515	37.50%
H120	3				*Haemophilus influenzae*	2842STDY5882047	2	GCF_900041425.1_14412_4_19	19.2	incomplete	50	19	166299-185570	Mannhe_vB_MhS_587AP2_NC_028743	38.37%
H120	4	*Lambda*	*Aaphi23-like*	*24*	*Haemophilus influenzae*	2842STDY5882047	2	GCF_900041425.1_14412_4_19	27.5	intact	103	44	486-27997	Aggreg_S1249_NC_013597	40.82%
H120	5				*Haemophilus influenzae*	2842STDY5882047	2	GCF_900041425.1_14412_4_19	11.2	intact	120	14	439-11735	Mannhe_vB_MhM_3927AP2_NC_02876	41.90%
H121	1				*Haemophilus influenzae*	2842STDY5882075	4	GCF_900044685.1_14412_4_47	28.5	intact	150	36	284542-313064	Mannhe_vB_MhM_3927AP2_NC_02876	42.44%
H121	2				*Haemophilus influenzae*	2842STDY5882075	4	GCF_900044685.1_14412_4_47	8.8	intact	120	16	47682-56540	Mannhe_vB_MhM_587AP1_NC_028898	39.61%
H122	1				*Haemophilus influenzae*	2842STDY5882033	2	GCF_900044695.1_14412_4_5	11.7	incomplete	30	16	127272-139066	Bacill_BCD7_NC_019515	37.50%
H122	2				*Haemophilus influenzae*	2842STDY5882033	2	GCF_900044695.1_14412_4_5	15.3	questionable	70	20	127851-143191	Shigel_SfIV_NC_022749	40.08%
H122	3				*Haemophilus influenzae*	2842STDY5882033	2	GCF_900044695.1_14412_4_5	10	incomplete	50	15	1-10097	Mannhe_vB_MhS_587AP2_NC_028743	38.77%
H122	4				*Haemophilus influenzae*	2842STDY5882033	2	GCF_900044695.1_14412_4_5	24.7	intact	100	39	83009-107793	Pseudo_JBD44_NC_030929	40.86%
H122	5	*Lambda*	*Gifsy2-like*	*31*	*Haemophilus influenzae*	2842STDY5882033	2	GCF_900044695.1_14412_4_5	40.4	intact	150	56	50693-91156	Mannhe_vB_MhS_587AP2_NC_028743	40.79%
H122	6				*Haemophilus influenzae*	2842STDY5882033	2	GCF_900044695.1_14412_4_5	10.3	incomplete	30	16	486-10869	Gordon_Lucky10_NC_031267	37.09%
H122	7				*Haemophilus influenzae*	2842STDY5882033	2	GCF_900044695.1_14412_4_5	10.6	intact	110	12	622-11292	Mannhe_vB_MhM_3927AP2_NC_02876	42.32%
H123	1				*Haemophilus influenzae*	2842STDY5882022	1	GCF_900044735.1_14412_3_89	21.3	intact	150	34	102474-123819	Mannhe_vB_MhM_587AP1_NC_028898	39.54%
H123	2				*Haemophilus influenzae*	2842STDY5882022	1	GCF_900044735.1_14412_3_89	25	questionable	80	38	229114-254116	Haemop_Aaphi23_NC_004827	37.96%
H123	3				*Haemophilus influenzae*	2842STDY5882022	1	GCF_900044735.1_14412_3_89	5.9	incomplete	40	8	373265-379187	Mannhe_vB_MhM_3927AP2_NC_02876	39.42%
H123	4	*Lambda*	*DIBBI-like*	*23*	*Haemophilus influenzae*	2842STDY5882022	1	GCF_900044735.1_14412_3_89	61	intact	150	74	63457-124497	Aggreg_S1249_NC_013597	39.27%
H124	1				*Haemophilus influenzae*	CCUG 26214	3	GCF_001679235.1_ASM167923v1	27.8	incomplete	30	8	572187-600039	Haemop_SuMu_NC_019455	37.57%
H124	2				*Haemophilus influenzae*	CCUG 26214	3	GCF_001679235.1_ASM167923v1	15.8	questionable	90	34	705044-720846	Aggreg_S1249_NC_013597	38.08%
H125	1	*Lambda*	*P22-like*	*36*	*Haemophilus influenzae*	CCUG 54503	3	GCF_001679365.1_ASM167936v1	68.2	intact	150	87	202275-270502	Mannhe_vB_MhS_587AP2_NC_028743	39.14%
H125	2				*Haemophilus influenzae*	CCUG 54503	3	GCF_001679365.1_ASM167936v1	25.4	incomplete	30	10	386494-411920	Haemop_SuMu_NC_019455	37.36%
H126	1				*Haemophilus influenzae*	CCUG 60490	3	GCF_001679415.1_ASM167941v1	28.6	incomplete	30	8	16908-45511	Haemop_SuMu_NC_019455	37.53%
H126	2				*Haemophilus influenzae*	CCUG 60490	3	GCF_001679415.1_ASM167941v1	12.9	incomplete	40	22	25501-38451	Mannhe_vB_MhS_1152AP2_NC_028956	36.70%

TABLE S2

Strain ID	Region	Type	Supercluster	Cluster	Species	Strain	Clade	Refseq/Genbank	Region Length	Completeness	Score	# Total Proteins	Region Position	Most Common Phage	GC %
H127	1				*Haemophilus influenzae*	NML-Hia-1	2	GCF_001856725.1_ASM185672v1	16.9	incomplete	50	28	58941-75843	Aggreg_S1249_NC_013597	39.08%
H127	2				*Haemophilus influenzae*	NML-Hia-1	2	GCF_001856725.1_ASM185672v1	7.5	questionable	90	12	196413-204001	Mannhe_vB_MhM_3927AP2_NC_02876(	39.20%
H128	1				*Haemophilus influenzae*	R535	3	GCF_001908955.1_ASM190895v1	9.7	incomplete	30	10	27263-37010	Haemop_SuMu_NC_019455	38.62%
H128	2				*Haemophilus influenzae*	R535	3	GCF_001908955.1_ASM190895v1	12.9	incomplete	40	17	17845-30796	Mannhe_vB_MhS_1152AP2_NC_028956	36.74%
H129	1				*Haemophilus influenzae*	1200	3	GCF_001909005.1_ASM190900v1	7.8	incomplete	30	9	3556-11416	Haemop_SuMu_NC_019455	39.18%
H129	2	*Lambda*	*P22-like*	*36*	*Haemophilus influenzae*	1200	3	GCF_001909005.1_ASM190900v1	64.6	intact	150	81	5813-70475	Mannhe_vB_MhS_587AP2_NC_028743	39.11%
H130	1	*Lambda*	*DIBBI-like*	*23*	*Haemophilus influenzae*	C188	2	GCF_001909015.1_ASM190901v1	46.6	intact	110	63	7551-54213	Salmon_BPS15Q2_NC_031939	41.26%
H130	2				*Haemophilus influenzae*	C188	2	GCF_001909015.1_ASM190901v1	6.3	incomplete	40	8	3637-9992	Mannhe_vB_MhM_3927AP2_NC_02876(	39.76%
H130	3	*Lambda*	*Aaphi23-like*	*24*	*Haemophilus influenzae*	C188	2	GCF_001909015.1_ASM190901v1	37.9	intact	110	40	14561-52482	Haemop_Aaphi23_NC_004827	37.93%
H131	1	*P1*	*MHaA1-like*	*19*	*Haemophilus influenzae*	65234 N Hi-1	1	GCF_001949825.1_ASM194982v1	25.2	intact	150	41	657381-682670	Mannhe_vB_MhM_587AP1_NC_028898	40.48%
H131	2	*Lambda*	*Gifsy2-like*	*31*	*Haemophilus influenzae*	65234 N Hi-1	1	GCF_001949825.1_ASM194982v1	59.9	intact	150	60	141958-201878	Pseudo_phi2_NC_030931	39.26%
H131	3	*Transposable*	*BcepMu-like*	*14*	*Haemophilus influenzae*	65234 N Hi-1	1	GCF_001949825.1_ASM194982v1	41.2	intact	100	56	63197-104454	Burkho_phiE255_NC_009237	39.54%
H131	4				*Haemophilus influenzae*	65234 N Hi-1	1	GCF_001949825.1_ASM194982v1	13.9	incomplete	60	23	290-14252	Acinet_LZ35_NC_031117	39.35%
H132	1				*Haemophilus influenzae*	65373 B Hi-3	5	GCF_001949855.1_ASM194985v1	35.2	questionable	80	37	272709-307940	Haemop_Aaphi23_NC_004827	38.35%
H132	2				*Haemophilus influenzae*	65373 B Hi-3	5	GCF_001949855.1_ASM194985v1	5.9	incomplete	40	8	413659-419653	Mannhe_vB_MhM_3927AP2_NC_02876(	39.70%
H132	3	*Lambda*	*Gifsy2-like*	*31*	*Haemophilus influenzae*	65373 B Hi-3	5	GCF_001949855.1_ASM194985v1	59.7	intact	150	62	1-59739	Pseudo_phi2_NC_030931	39.60%
H132	4				*Haemophilus influenzae*	65373 B Hi-3	5	GCF_001949855.1_ASM194985v1	19.8	intact	150	25	169847-189730	Mannhe_phiMHaA1_NC_008201	40.92%
H132	5	*P1*	*HP1-like*	*21*	*Haemophilus influenzae*	65373 B Hi-3	5	GCF_001949855.1_ASM194985v1	28.4	intact	113	45	1516-29976	Haemop_HP1_NC_001697	40.02%
H132	6				*Haemophilus influenzae*	65373 B Hi-3	5	GCF_001949855.1_ASM194985v1	12.9	questionable	90	24	809-13750	Mannhe_vB_MhM_587AP1_NC_028898	41.00%
H133	1				*Haemophilus influenzae*	65234 B Hi-2	1	GCF_001949875.1_ASM194987v1	12.8	incomplete	60	21	2-12833	Aggreg_S1249_NC_013597	39.36%
H133	2	*P1*	*MHaA1-like*	*19*	*Haemophilus influenzae*	65234 B Hi-2	1	GCF_001949875.1_ASM194987v1	25.2	intact	150	42	131045-156334	Mannhe_vB_MhM_587AP1_NC_028898	40.48%
H133	3	*Transposable*	*BcepMu-like*	*14*	*Haemophilus influenzae*	65234 B Hi-2	1	GCF_001949875.1_ASM194987v1	41.9	intact	110	57	56389-98357	Burkho_phiE255_NC_009237	39.48%
H133	4				*Haemophilus influenzae*	65234 B Hi-2	1	GCF_001949875.1_ASM194987v1	5.9	incomplete	40	8	159014-164935	Mannhe_vB_MhM_3927AP2_NC_02876(	39.36%
H133	5				*Haemophilus influenzae*	65234 B Hi-2	1	GCF_001949875.1_ASM194987v1	24.5	incomplete	20	23	3683-28273	Mannhe_vB_MhS_587AP2_NC_028743	39.64%
H133	6				*Haemophilus influenzae*	65234 B Hi-2	1	GCF_001949875.1_ASM194987v1	16.6	intact	100	20	62-16698	Pseudo_MD8_NC_031091	41.08%
H134	1				*Haemophilus influenzae*	hi467	4	GCF_001975845.1_ASM197584v1	23.3	intact	150	31	3-23333	Haemop_SuMu_NC_019455	41.34%
H135	1	*Lambda*	*Aaphi23-like*	*24*	*Haemophilus influenzae*	ATCC 10211	2	GCF_001997355.1_ASM199735v1	27.5	intact	106	45	3-27601	Aggreg_S1249_NC_013597	40.82%
H135	2				*Haemophilus influenzae*	ATCC 10211	2	GCF_001997355.1_ASM199735v1	14.9	intact	120	20	677-15653	Mannhe_vB_MhM_3927AP2_NC_02876(	40.76%
H135	3				*Haemophilus influenzae*	ATCC 10211	2	GCF_001997355.1_ASM199735v1	15.3	questionable	70	20	153710-169039	Shigel_SfIV_NC_022749	40.08%
H135	4				*Haemophilus influenzae*	ATCC 10211	2	GCF_001997355.1_ASM199735v1	11.7	incomplete	30	16	151582-163377	Bacill_BCD7_NC_019515	37.50%
H135	5				*Haemophilus influenzae*	ATCC 10211	2	GCF_001997355.1_ASM199735v1	20.4	incomplete	40	12	37155-57650	Mannhe_vB_MhS_587AP2_NC_028743	38.13%
H136	1	*Transposable*	*Mu-like*	*09*	*Haemophilus ducreyi*	35000HP		GCF_000007945.1_ASM794v1	35.7	intact	150	56	71207-106940	Escher_D108_NC_013594	47.54%

TABLE S2

Strain ID	Region	Type	Supercluster	Cluster	Species	Strain	Clade	Refseq/Genbank	Region Length	Completeness	Score	# Total Proteins	Region Position	Most Common Phage	GC %
H136	2	*Transposable*	*SuMu-like*	*05*	*Haemophilus ducreyi*	35000HP		GCF_000007945.1_ASM794v1	38.6	intact	150	51	373282-411901	Mannhe_vB_MhM_3927AP2_NC_02876	41.89%
H136	3	*Transposable*	*B3-like*	*12*	*Haemophilus ducreyi*	35000HP		GCF_000007945.1_ASM794v1	36.9	intact	150	49	1262080-1299062	Pseudo_vB_PaeS_PM105_NC_028667	41.49%
H137	1	*P1*	*MHaA1-like*	*20*	*Haemophilus parainfluenzae*	T3T1		GCF_000210895.1_ASM21089v1	30.8	intact	150	46	403579-434388	Mannhe_vB_MhM_587AP1_NC_028898	41.71%
H138	1				*Haemophilus parainfluenzae*	ATCC 33392		GCF_000191405.1_ASM19140v1	8.1	incomplete	10	8	1725388-1733547	Gordon_Bantam_NC_031074	37.29%
H139	1	*Lambda*	*DIBBI-like*	*23*	*Haemophilus aegyptius*	ATCC 11116		GCF_000195005.1_ASM19500v1	33.3	intact	110	49	838-34155	Aggreg_S1249_NC_013597	39.00%
H139	2	*P1*	*MHaA1-like*	*19*	*Haemophilus aegyptius*	ATCC 11116		GCF_000195005.1_ASM19500v1	35.3	intact	150	56	372733-408072	Mannhe_vB_MhM_587AP1_NC_028898	40.68%
H139	3	*Transposable*	*SuMu-like*	*01*	*Haemophilus aegyptius*	ATCC 11116		GCF_000195005.1_ASM19500v1	45.3	intact	150	57	481839-527151	Haemop_SuMu_NC_019455	37.06%
H139	4				*Haemophilus aegyptius*	ATCC 11116		GCF_000195005.1_ASM19500v1	7.6	incomplete	30	11	3-7645	Haemop_HP1_NC_001697	33.52%
H139	5	*Lambda*	*Aaphi23-like*	*24*	*Haemophilus aegyptius*	ATCC 11116		GCF_000195005.1_ASM19500v1	30.6	intact	100	51	731-31339	Gordon_SoilAssassin_NC_031251	39.59%
H139	6				*Haemophilus aegyptius*	ATCC 11116		GCF_000195005.1_ASM19500v1	16.8	incomplete	30	17	152296-169130	Bacill_BCD7_NC_019515	38.49%
H140	1				*Haemophilus sputorum*	CCUG 13788		GCF_000238795.1_ASM23879v2	14.7	intact	100	18	108637-123375	Salmon_ST64B_NC_004313	38.24%
H140	2				*Haemophilus sputorum*	CCUG 13788		GCF_000238795.1_ASM23879v2	36.7	questionable	90	57	147912-184650	Burkho_BcepMu_NC_005882	43.91%
H141	1				*Haemophilus haemolyticus*	M19501		GCF_000222025.1_ASM22202v2	8	incomplete	10	9	88289-96298	Vibrio_KVP40_NC_005083	37.25%
H141	2				*Haemophilus haemolyticus*	M19501		GCF_000222025.1_ASM22202v2	14.6	incomplete	10	12	245031-259726	Bacill_G_NC_023719	39.59%
H142	1	*P1*	*MHaA1-like*	*19*	*Haemophilus haemolyticus*	M21127		GCF_000222045.1_ASM22204v2	50.1	intact	150	59	51135-101244	Mannhe_vB_MhM_587AP1_NC_028898	40.42%
H142	2				*Haemophilus haemolyticus*	M21127		GCF_000222045.1_ASM22204v2	30.9	questionable	90	37	86844-117759	Entero_mEp235_NC_019708	39.11%
H142	3	*Lambda*	*Gifsy2-like*	*31*	*Haemophilus haemolyticus*	M21127		GCF_000222045.1_ASM22204v2	29.5	intact	150	42	120868-150454	Mannhe_vB_MhS_587AP2_NC_028743	40.54%
H143	1	*Lambda*	*1152AP2-like*	*29*	*Haemophilus haemolyticus*	M21639		GCF_000222085.1_ASM22208v2	28	intact	110	44	80-28112	Mannhe_vB_MhS_1152AP2_NC_028956	41.09%
H143	2				*Haemophilus haemolyticus*	M21639		GCF_000222085.1_ASM22208v2	28.8	questionable	90	42	1-28800	Salmon_118970_sal3_NC_031940	41.41%
H143	3				*Haemophilus haemolyticus*	M21639		GCF_000222085.1_ASM22208v2	18.4	incomplete	60	31	712-19117	Salmon_IME207_NC_031924	38.82%
H143	4				*Haemophilus haemolyticus*	M21639		GCF_000222085.1_ASM22208v2	26.7	intact	117	39	1613-28333	Haemop_HP1_NC_001697	40.18%
H143	5				*Haemophilus haemolyticus*	M21639		GCF_000222085.1_ASM22208v2	20.6	intact	120	25	1-20638	Mannhe_vB_MhM_3927AP2_NC_02876	42.98%
H143	6				*Haemophilus haemolyticus*	M21639		GCF_000222085.1_ASM22208v2	17.3	questionable	90	32	3-17385	Haemop_HP1_NC_001697	40.75%
H143	7				*Haemophilus haemolyticus*	M21639		GCF_000222085.1_ASM22208v2	13.3	questionable	70	21	4881-18275	Mannhe_vB_MhS_587AP2_NC_028743	39.81%
H143	8				*Haemophilus haemolyticus*	M21639		GCF_000222085.1_ASM22208v2	30.3	incomplete	60	42	220-30600	Haemop_Aaphi23_NC_004827	37.92%
H143	9	*Transposable*	*Mu-like*	*06*	*Haemophilus haemolyticus*	M21639		GCF_000222085.1_ASM22208v2	40	intact	150	59	30032-70114	Escher_D108_NC_013594	43.33%
H143	10				*Haemophilus haemolyticus*	M21639		GCF_000222085.1_ASM22208v2	16.4	intact	150	23	1-16428	Mannhe_phiMHaA1_NC_008201	41.08%
H143	11				*Haemophilus haemolyticus*	M21639		GCF_000222085.1_ASM22208v2	18.6	questionable	80	25	23281-41915	Shigel_SfIV_NC_022749	38.78%
H143	12	*Transposable*	*BcepMu-like*		*Haemophilus haemolyticus*	M21639		GCF_000222085.1_ASM22208v2	40.6	intact	150	51	8025-48705	Burkho_phiE255_NC_009237	42.29%
H143	13				*Haemophilus haemolyticus*	M21639		GCF_000222085.1_ASM22208v2	16.4	questionable	70	24	1830-18250	Mannhe_vB_MhS_587AP2_NC_028743	39.69%
H143	14				*Haemophilus haemolyticus*	M21639		GCF_000222085.1_ASM22208v2	10.2	incomplete	20	15	115646-125937	Burkho_phiE255_NC_009237	38.64%
H144	1				*Haemophilus haemolyticus*	M19107		GCF_000222005.1_ASM22200v2	6.2	incomplete	20	9	24320-30577	Bacill_SP_10_NC_019487	38.86%

TABLE S2

Strain ID	Region	Type	Supercluster	Cluster	Species	Strain	Clade	Refseq/Genbank	Region Length	Completeness	Score	# Total Proteins	Region Position	Most Common Phage	GC %
H144	2				*Haemophilus haemolyticus*	M19107		GCF_000222005.1_ASM22200v2	6.7	questionable	70	9	956-7749	Shigel_SfIV_NC_022749	41.76%
H144	3				*Haemophilus haemolyticus*	M19107		GCF_000222005.1_ASM22200v2	8.6	incomplete	10	8	2070-10713	Bacill_G_NC_023719	39.43%
H145	1				*Haemophilus haemolyticus*	M21621		GCF_000222065.1_ASM22206v1	12.8	incomplete	30	9	59149-71951	Brevib_Jenst_NC_028805	36.48%
H145	2				*Haemophilus haemolyticus*	M21621		GCF_000222065.1_ASM22206v1	8.6	incomplete	10	10	57786-66428	Bacill_G_NC_023719	39.44%
H145	3				*Haemophilus haemolyticus*	M21621		GCF_000222065.1_ASM22206v1	8.3	incomplete	20	8	82717-91066	Bacill_G_NC_023719	38.99%
H145	4				*Haemophilus haemolyticus*	M21621		GCF_000222065.1_ASM22206v1	7.1	incomplete	10	12	64562-71729	Gordon_Bantam_NC_031074	36.19%
H145	5				*Haemophilus haemolyticus*	M21621		GCF_000222065.1_ASM22206v1	8.2	incomplete	20	8	292638-300861	Bacill_G_NC_023719	38.96%
H145	6				*Haemophilus haemolyticus*	M21621		GCF_000222065.1_ASM22206v1	8.6	incomplete	10	9	317153-325795	Bacill_G_NC_023719	39.44%
H146	1				*Haemophilus* sp. HMT-851	F0397		GCF_000242295.1_ASM24229v1	19	incomplete	60	24	148024-167095	Shigel_SfIV_NC_022749	37.51%
H146	2				*Haemophilus* sp. HMT-851	F0397		GCF_000242295.1_ASM24229v1	21	incomplete	60	26	26648-47736	Bacill_0305phi8_36_NC_009760	36.04%
H147	1	*Transposable*	*SuMu-like*	*02*	*Haemophilus parainfluenzae*	HK262		GCF_000259485.1_HparainfluenzaeHK262v1.0	35.6	intact	150	49	134-35779	Haemop_SuMu_NC_019455	42.16%
H147	2				*Haemophilus parainfluenzae*	HK262		GCF_000259485.1_HparainfluenzaeHK262v1.0	27.6	questionable	70	22	6101-33777	Salmon_ST64B_NC_004313	37.84%
H148	1				*Haemophilus paraphrohaemolyticus*	HK411		GCF_000260675.1_HparaphrohaemolyticusHK411v	17.2	intact	130	19	1-17210	Entero_c_1_NC_019706	41.92%
H148	2	*Transposable*	*BcepMu-like*	*18*	*Haemophilus paraphrohaemolyticus*	HK411		GCF_000260675.1_HparaphrohaemolyticusHK411v	39.6	intact	100	63	205-39890	Burkho_BcepMu_NC_005882	44.13%
H148	3				*Haemophilus paraphrohaemolyticus*	HK411		GCF_000260675.1_HparaphrohaemolyticusHK411v	7.6	incomplete	30	13	1-7614	Escher_D108_NC_013594	43.43%
H148	4				*Haemophilus paraphrohaemolyticus*	HK411		GCF_000260675.1_HparaphrohaemolyticusHK411v	17.6	questionable	70	23	1-17625	Mannhe_vB_MhS_1152AP2_NC_028956	43.11%
H149	1				*Haemophilus parainfluenzae*	HK2019		GCF_000261285.1_HparainfluenzaeHK2019v1.0	27.6	questionable	70	22	224155-251831	Salmon_ST64B_NC_004313	37.84%
H149	2	*P1*	*MHaA1-like*	*20*	*Haemophilus parainfluenzae*	HK2019		GCF_000261285.1_HparainfluenzaeHK2019v1.0	32.7	intact	150	51	44-32755	Mannhe_vB_MhM_587AP1_NC_028898	41.14%
H150	1	*Transposable*	*SuMu-like*	*01*	*Haemophilus haemolyticus*	HK386		GCF_000262285.1_ASM26228v1	39.6	intact	150	52	44461-84119	Haemop_SuMu_NC_019455	41.89%
H151	1	*Lambda*	*1152AP2-like*	*28*	*Haemophilus parahaemolyticus*	HK385		GCF_000262265.1_ASM26226v1	27.5	intact	110	49	73-27630	Mannhe_vB_MhS_1152AP2_NC_028956	41.84%
H151	2				*Haemophilus parahaemolyticus*	HK385		GCF_000262265.1_ASM26226v1	9.4	incomplete	20	15	31319-40810	Acinet_vB_AbaS_TRS1_NC_031098	40.94%
H151	3	*P1*	*H1-like*		*Haemophilus parahaemolyticus*	HK385		GCF_000262265.1_ASM26226v1	36.2	intact	120	43	35135-71384	Haemop_HP2_NC_003315	42.22%
H152	1				*Haemophilus sputorum*	HK 2154		GCF_000287615.1_HsputHK2154v1.0	36.7	questionable	90	57	415736-452474	Burkho_BcepMu_NC_005882	43.91%
H152	2				*Haemophilus sputorum*	HK 2154		GCF_000287615.1_HsputHK2154v1.0	14.7	intact	100	21	606410-621148	Salmon_ST64B_NC_004313	38.25%
H153	1				*Haemophilus parainfluenzae*	ATCC 33392		GCF_000746485.1_ASM74648v1	8.1	incomplete	10	8	12158-20317	Gordon_Bowser_NC_030930	37.29%
H154	1				*Haemophilus parahaemolyticus*	G321		GCF_000826045.1_Haemophilus_parahaemolyticus_	22.4	questionable	80	29	388037-410438	Mannhe_vB_MhS_1152AP2_NC_028956	42.76%
H155	1				*Haemophilus haemolyticus*	11P18		GCF_001008205.1_ASM100820v1	18.1	questionable	70	23	17928-36100	Shigel_SfIV_NC_022749	37.83%
H156	1	*Lambda*	*1152AP2-like*	*30*	*Haemophilus haemolyticus*	1P26		GCF_001008215.1_ASM100821v1	26.9	intact	120	43	1-26948	Mannhe_vB_MhS_1152AP2_NC_028956	40.02%
H156	2				*Haemophilus haemolyticus*	1P26		GCF_001008215.1_ASM100821v1	30.4	incomplete	60	39	6748-37189	Mannhe_vB_MhS_587AP2_NC_028743	39.03%
H157	1				*Haemophilus haemolyticus*	3P5		GCF_001008225.1_ASM100822v1	24.7	questionable	90	21	49371-74113	Shigel_SfIV_NC_022749	38.78%
H158	1				*Haemophilus haemolyticus*	27P25		GCF_001008275.1_ASM100827v1	5.8	incomplete	10	9	6840-12643	Gordon_Bantam_NC_031074	37.06%
H158	2				*Haemophilus haemolyticus*	27P25		GCF_001008275.1_ASM100827v1	12	incomplete	10	14	23623-35668	Bacill_G_NC_023719	38.49%

TABLE S2

Strain ID	Region	Type	Supercluster	Cluster	Species	Strain	Clade	Refseq/Genbank	Region Length	Completeness	Score	# Total Proteins	Region Position	Most Common Phage	GC %
H158	3				*Haemophilus haemolyticus*	27P25		GCF_001008275.1_ASM100827v1	8.3	incomplete	20	8	48560-56918	Bacill_G_NC_023719	39.20%
H159	1	*Transposable*	*Mu-like*	*09*	*Haemophilus ducreyi*	CLU1	1	GCA_001043155.1_ASM104315v1	35.7	intact	150	58	70688-106443	Escher_D108_NC_013594	47.56%
H159	2	*Transposable*	*SuMu-like*	*05*	*Haemophilus ducreyi*	CLU1	1	GCA_001043155.1_ASM104315v1	43.1	intact	150	61	363565-406713	Mannhe_vB_MhM_3927AP2_NC_02876	41.30%
H160	1	*Transposable*	*Mu-like*	*09*	*Haemophilus ducreyi*	CLU2	1	GCA_001043195.1_ASM104319v1	35.7	intact	150	58	71940-107695	Escher_D108_NC_013594	47.56%
H160	2	*Transposable*	*SuMu-like*	*05*	*Haemophilus ducreyi*	CLU2	1	GCA_001043195.1_ASM104319v1	38.2	intact	150	53	368046-406285	Mannhe_vB_MhM_3927AP2_NC_02876	41.90%
H161	1	*Transposable*	*Mu-like*	*09*	*Haemophilus ducreyi*	CLU3	1	GCA_001043235.1_ASM104323v1	35.7	intact	150	58	71428-107183	Escher_D108_NC_013594	47.56%
H161	2	*Transposable*	*SuMu-like*	*05*	*Haemophilus ducreyi*	CLU3	1	GCA_001043235.1_ASM104323v1	38.8	intact	150	54	367463-406336	Mannhe_vB_MhM_3927AP2_NC_02876	41.87%
H162	1	*Transposable*	*Mu-like*	*09*	*Haemophilus ducreyi*	CLU4	1	GCA_001043275.1_ASM104327v1	35.7	intact	150	58	70869-106624	Escher_D108_NC_013594	47.56%
H162	2	*Transposable*	*SuMu-like*	*05*	*Haemophilus ducreyi*	CLU4	1	GCA_001043275.1_ASM104327v1	38.7	intact	150	54	365415-404150	Mannhe_vB_MhM_3927AP2_NC_02876	41.87%
H163	1	*Transposable*	*Mu-like*	*09*	*Haemophilus ducreyi*	GU1	1	GCA_001043315.1_ASM104331v1	32.5	intact	150	54	72443-104978	Escher_D108_NC_013594	47.45%
H163	2	*Transposable*	*SuMu-like*	*05*	*Haemophilus ducreyi*	GU1	1	GCA_001043315.1_ASM104331v1	31.4	intact	120	43	360568-391967	Mannhe_vB_MhM_3927AP2_NC_02876	41.71%
H163	3				*Haemophilus ducreyi*	GU1	1	GCA_001043315.1_ASM104331v1	24.7	intact	100	39	1233752-1258519	Pseudo_vB_PaeS_PM105_NC_028667	39.94%
H164	1	*Transposable*	*Mu-like*	*09*	*Haemophilus ducreyi*	GU2	1	GCA_001043335.1_ASM104333v1	32.8	intact	150	54	70425-103261	Escher_D108_NC_013594	47.48%
H164	2	*Transposable*	*SuMu-like*	*05*	*Haemophilus ducreyi*	GU2	1	GCA_001043335.1_ASM104333v1	31.4	intact	140	46	358698-390110	Mannhe_vB_MhM_3927AP2_NC_02876	41.70%
H164	3				*Haemophilus ducreyi*	GU2	1	GCA_001043335.1_ASM104333v1	24.5	intact	100	39	1251695-1276284	Pseudo_vB_PaeS_PM105_NC_028667	39.88%
H165	1	*Transposable*	*Mu-like*	*09*	*Haemophilus ducreyi*	GU3	1	GCA_001043355.1_ASM104335v1	35.5	intact	150	57	68873-104468	Escher_D108_NC_013594	47.56%
H165	2	*Transposable*	*SuMu-like*	*05*	*Haemophilus ducreyi*	GU3	1	GCA_001043355.1_ASM104335v1	31.3	intact	120	44	360158-391544	Mannhe_vB_MhM_3927AP2_NC_02876	41.70%
H165	3				*Haemophilus ducreyi*	GU3	1	GCA_001043355.1_ASM104335v1	24.4	intact	100	39	1231114-1255540	Pseudo_vB_PaeS_PM105_NC_028667	40.08%
H166	1	*Transposable*	*Mu-like*	*09*	*Haemophilus ducreyi*	GU4	1	GCA_001043375.1_ASM104337v1	32.5	intact	150	53	71028-103592	Escher_D108_NC_013594	47.45%
H166	2	*Transposable*	*SuMu-like*	*05*	*Haemophilus ducreyi*	GU4	1	GCA_001043375.1_ASM104337v1	32	intact	130	45	357329-389335	Mannhe_vB_MhM_3927AP2_NC_02876	41.58%
H166	3	*Transposable*	*B3-like*	*12*	*Haemophilus ducreyi*	GU4	1	GCA_001043375.1_ASM104337v1	35.6	intact	150	50	1199262-1234922	Pseudo_vB_PaeS_PM105_NC_028667	41.17%
H167	1	*Transposable*	*Mu-like*	*09*	*Haemophilus ducreyi*	GU5	1	GCA_001043395.1_ASM104339v1	32.5	intact	150	53	70557-103121	Escher_D108_NC_013594	47.45%
H167	2	*Transposable*	*SuMu-like*	*05*	*Haemophilus ducreyi*	GU5	1	GCA_001043395.1_ASM104339v1	31.6	intact	140	47	357926-389535	Mannhe_vB_MhM_3927AP2_NC_02876	41.57%
H167	3				*Haemophilus ducreyi*	GU5	1	GCA_001043395.1_ASM104339v1	24.6	intact	100	39	1180657-1205295	Pseudo_vB_PaeS_PM105_NC_028667	39.87%
H168	1	*Transposable*	*SuMu-like*	*05*	*Haemophilus ducreyi*	CLU5	1	GCA_001043415.1_ASM104341v1	33.5	intact	150	54	154538-188058	Mannhe_vB_MhM_3927AP2_NC_02876	39.83%
H168	2	*Transposable*	*Mu-like*	*09*	*Haemophilus ducreyi*	CLU5	1	GCA_001043415.1_ASM104341v1	36.1	intact	150	59	432224-468394	Escher_D108_NC_013594	47.55%
H168	3				*Haemophilus ducreyi*	CLU5	1	GCA_001043415.1_ASM104341v1	14.2	incomplete	50	17	1568425-1582644	Pseudo_vB_PaeS_PM105_NC_028667	42.97%
H169	1				*Haemophilus ducreyi*	GU6	2	GCA_001043435.1_ASM104343v1	25.5	intact	150	31	70692-96218	Entero_SfMu_NC_027382	43.77%
H169	2				*Haemophilus ducreyi*	GU6	2	GCA_001043435.1_ASM104343v1	12.9	incomplete	10	21	355013-367932	Haemop_SuMu_NC_019455	37.38%
H169	3	*Transposable*	*B3-like*	*12*	*Haemophilus ducreyi*	GU6	2	GCA_001043435.1_ASM104343v1	23	intact	130	40	1152457-1175538	Pseudo_vB_PaeS_PM105_NC_028667	40.62%
H170	1				*Haemophilus ducreyi*	GU7	2	GCA_001043455.1_ASM104345v1	26.2	intact	150	30	71033-97272	Entero_SfMu_NC_027382	44.01%
H170	2				*Haemophilus ducreyi*	GU7	2	GCA_001043455.1_ASM104345v1	13.1	incomplete	10	22	351729-364835	Haemop_SuMu_NC_019455	37.42%

TABLE S2

Strain ID	Region	Type	Supercluster	Cluster	Species	Strain	Clade	Refseq/Genbank	Region Length	Completeness	Score	# Total Proteins	Region Position	Most Common Phage	GC %
H170	3	*Transposable*	*B3-like*	*12*	*Haemophilus ducreyi*	GU7	2	GCA_001043455.1_ASM104345v1	24.2	intact	130	41	1136337-1160597	Pseudo_vB_PaeS_PM105_NC_028667	40.68%
H171	1				*Haemophilus ducreyi*	GU8	2	GCA_001043475.1_ASM104347v1	26.1	intact	150	30	70594-96716	Entero_SfMu_NC_027382	43.99%
H171	2				*Haemophilus ducreyi*	GU8	2	GCA_001043475.1_ASM104347v1	12.9	incomplete	10	21	354864-367794	Haemop_SuMu_NC_019455	37.40%
H171	3	*Transposable*	*B3-like*	*12*	*Haemophilus ducreyi*	GU8	2	GCA_001043475.1_ASM104347v1	24.2	intact	130	43	1142299-1166524	Pseudo_vB_PaeS_PM105_NC_028667	40.66%
H171	4				*Haemophilus ducreyi*	GU8	2	GCA_001043475.1_ASM104347v1	15.4	incomplete	30	25	1567901-1583330	Haemop_SuMu_NC_019455	41.48%
H172	1				*Haemophilus ducreyi*	GU9	2	GCA_001043495.1_ASM104349v1	25.5	intact	150	32	70006-95575	Entero_SfMu_NC_027382	43.82%
H172	2				*Haemophilus ducreyi*	GU9	2	GCA_001043495.1_ASM104349v1	14.4	incomplete	10	25	355231-369687	Haemop_SuMu_NC_019455	37.87%
H172	3	*Transposable*	*B3-like*	*12*	*Haemophilus ducreyi*	GU9	2	GCA_001043495.1_ASM104349v1	23	intact	130	40	1136650-1159731	Pseudo_vB_PaeS_PM105_NC_028667	40.61%
H173	1	*Lambda*	*587AP2-like*	*26*	*Haemophilus parainfluenzae*	155_HPAR		GCF_001054475.1_ASM105447v1	38.5	intact	150	61	56576-95142	Mannhe_vB_MhS_587AP2_NC_028743	41.05%
H173	2				*Haemophilus parainfluenzae*	155_HPAR		GCF_001054475.1_ASM105447v1	16.2	incomplete	30	8	1-16221	Mannhe_vB_MhS_587AP2_NC_028743	40.45%
H173	3				*Haemophilus parainfluenzae*	155_HPAR		GCF_001054475.1_ASM105447v1	24.2	questionable	80	18	29318-53612	Shigel_SfIV_NC_022749	37.88%
H174	1				*Haemophilus parainfluenzae*	432_HPAR		GCF_001055095.1_ASM105509v1	6.8	incomplete	10	10	45232-52113	Bacill_SP_15_NC_031245	37.84%
H175	1	*Transposable*	*BcepMu-like*	*15*	*Haemophilus parainfluenzae*	174_HPAR		GCF_001055595.1_ASM105559v1	62.5	intact	150	91	44390-106920	Burkho_BcepMu_NC_005882	43.37%
H176	-				*Haemophilus parainfluenzae*	488_HPAR		GCF_001057005.1_ASM105700v1	-	no prophage	-	-	-	-	-
H177	1	*Lambda*	*HK97-like*	*35*	*Haemophilus parainfluenzae*	901_HPAR		GCF_001059815.1_ASM105981v1	40.6	intact	150	56	216-40866	Mannhe_vB_MhS_587AP2_NC_028743	38.77%
H178	1	*P1*	*HP1-like*	*22*	*Haemophilus parainfluenzae*	1209_HPAR		GCF_001053035.1_ASM105303v1	30.3	intact	125	40	217-30540	Haemop_HP1_NC_001697	40.52%
H179	1	*Transposable*	*BcepMu-like*	*15*	*Haemophilus parainfluenzae*	137_HINF		GCF_001053535.1_ASM105353v1	62.5	intact	150	91	44387-106914	Burkho_BcepMu_NC_005882	43.38%
H180	1				*Haemophilus parainfluenzae*	146_HPAR		GCF_001053575.1_ASM105357v1	18.6	intact	150	26	464-19080	Mannhe_vB_MhM_587AP1_NC_028898	40.86%
H180	2	*Lambda*	*HK97-like*	*35*	*Haemophilus parainfluenzae*	146_HPAR		GCF_001053575.1_ASM105357v1	38.5	intact	150	53	467-38976	Mannhe_vB_MhS_587AP2_NC_028743	38.52%
H180	3				*Haemophilus parainfluenzae*	146_HPAR		GCF_001053575.1_ASM105357v1	25.5	questionable	80	29	30512-56101	Mannhe_vB_MhM_587AP1_NC_028898	40.26%
H181	1				*Haemophilus parainfluenzae*	1128_HPAR		GCF_001053915.1_ASM105391v1	24.7	questionable	90	20	30723-55475	Shigel_SfII_NC_021857	37.30%
H182	1				*Haemophilus parainfluenzae*	209_HPAR		GCF_001055885.1_ASM105588v1	6	incomplete	10	7	20754-26772	Gordon_Bowser_NC_030930	35.99%
H183	1				*Haemophilus parainfluenzae*	777_HPAR		GCF_001058435.1_ASM105843v1	21.9	incomplete	50	45	1-21947	Mannhe_vB_MhS_1152AP2_NC_028956	38.92%
H183	2				*Haemophilus parainfluenzae*	777_HPAR		GCF_001058435.1_ASM105843v1	25.1	intact	120	32	334-25503	Mannhe_vB_MhS_535AP2_NC_028853	41.67%
H183	3				*Haemophilus parainfluenzae*	777_HPAR		GCF_001058435.1_ASM105843v1	24.2	questionable	80	18	12095-36389	Shigel_SfIV_NC_022749	37.87%
H184	1	*P1*	*MHaA1-like*	*19*	*Haemophilus haemolyticus*	C1		GCF_001276515.1_ASM127651v1	32.8	intact	150	56	102-32918	Mannhe_phiMHaA1_NC_008201	40.36%
H184	2				*Haemophilus haemolyticus*	C1		GCF_001276515.1_ASM127651v1	12.8	incomplete	40	10	34463-47351	Entero_P4_NC_001609	37.82%
H184	3	*Lambda*	*Aaphi23-like*	*24*	*Haemophilus haemolyticus*	C1		GCF_001276515.1_ASM127651v1	48	intact	140	78	31773-79791	Aggreg_S1249_NC_013597	40.38%
H185	1				*Haemophilus parainfluenzae*	215035-2-ISO5		GCF_001279145.1_ASM127914v1	7.7	incomplete	20	11	162970-170741	Gordon_Bowser_NC_030930	36.34%
H185	2				*Haemophilus parainfluenzae*	215035-2-ISO5		GCF_001279145.1_ASM127914v1	6.5	incomplete	10	10	173935-180462	Bacill_G_NC_023719	37.58%
H186	1	*Transposable*	*SuMu-like*	*05*	*Haemophilus ducreyi*	AUSPNG1	1	GCF_001465235.1_ASM146523v1	39	intact	150	54	314188-353286	Mannhe_vB_MhM_3927AP2_NC_02876[illegible]	41.71%
H186	2	*Transposable*	*Mu-like*	*09*	*Haemophilus ducreyi*	AUSPNG1	1	GCF_001465235.1_ASM146523v1	35.7	intact	150	57	117463-153184	Escher_D108_NC_013594	47.53%

TABLE S2

Strain ID	Region	Type	Supercluster	Cluster	Species	Strain	Clade	Refseq/Genbank	Region Length	Completeness	Score	# Total Proteins	Region Position	Most Common Phage	GC %
H186	3				*Haemophilus ducreyi*	AUSPNG1	1	GCF_001465235.1_ASM146523v1	22.8	intact	110	36	11863-34742	Pseudo_vB_PaeS_PM105_NC_028667	40.15%
H187	1	*Transposable*	*SuMu-like*	*05*	*Haemophilus ducreyi*	VAN1	1	GCF_001647655.1_ASM164765v1	38.6	intact	150	51	57402-96022	Mannhe_vB_MhM_3927AP2_NC_02876	41.88%
H187	2	*Transposable*	*Mu-like*	*09*	*Haemophilus ducreyi*	VAN1	1	GCF_001647655.1_ASM164765v1	35.6	intact	150	55	1422531-1458223	Escher_D108_NC_013594	47.54%
H188	1	*Transposable*	*Mu-like*	*08*	*Haemophilus ducreyi*	VAN2	2	GCF_001647695.1_ASM164769v1	34.1	intact	150	50	428386-462502	Escher_D108_NC_013594	42.74%
H188	2	*Transposable*	*B3-like*	*12*	*Haemophilus ducreyi*	VAN2	2	GCF_001647695.1_ASM164769v1	35.7	intact	150	54	996670-1032448	Pseudo_vB_PaeS_PM105_NC_028667	42.12%
H189	1	*Transposable*	*Mu-like*	*09*	*Haemophilus ducreyi*	VAN3	1	GCF_001647735.1_ASM164773v1	35.6	intact	150	55	259644-295336	Escher_D108_NC_013594	47.54%
H189	2	*Transposable*	*SuMu-like*	*05*	*Haemophilus ducreyi*	VAN3	1	GCF_001647735.1_ASM164773v1	38.6	intact	150	51	561606-600226	Mannhe_vB_MhM_3927AP2_NC_02876	41.88%
H190	1	*Transposable*	*SuMu-like*	*05*	*Haemophilus ducreyi*	VAN4	1	GCF_001647765.1_ASM164776v1	38.6	intact	150	51	1298902-1337522	Mannhe_vB_MhM_3927AP2_NC_02876	41.88%
H190	2	*Transposable*	*Mu-like*	*09*	*Haemophilus ducreyi*	VAN4	1	GCF_001647765.1_ASM164776v1	35.6	intact	150	56	1604174-1639866	Escher_D108_NC_013594	47.54%
H191	1	*Transposable*	*SuMu-like*	*05*	*Haemophilus ducreyi*	VAN5	1	GCF_001647795.1_ASM164779v1	38.6	intact	150	51	594890-633510	Mannhe_vB_MhM_3927AP2_NC_02876	41.88%
H191	2	*Transposable*	*Mu-like*	*09*	*Haemophilus ducreyi*	VAN5	1	GCF_001647795.1_ASM164779v1	35.6	intact	150	56	900156-935848	Escher_D108_NC_013594	47.54%
H192	1				*Haemophilus ducreyi*	GHA1	2	GCF_001647815.1_ASM164781v1	8.8	incomplete	20	17	330-9153	Burkho_phiE255_NC_009237	41.07%
H192	2	*Transposable*	*Mu-like*	*08*	*Haemophilus ducreyi*	GHA1	2	GCF_001647815.1_ASM164781v1	34.1	intact	150	50	997434-1031550	Escher_D108_NC_013594	42.74%
H192	3				*Haemophilus ducreyi*	GHA1	2	GCF_001647815.1_ASM164781v1	25.2	intact	140	31	1595533-1620819	Pseudo_vB_PaeS_PM105_NC_028667	42.84%
H193	1	*Transposable*	*B3-like*	*12*	*Haemophilus ducreyi*	GHA2	2	GCF_001647835.1_ASM164783v1	35.7	intact	150	52	726950-762726	Pseudo_vB_PaeS_PM105_NC_028667	42.11%
H193	2	*Transposable*	*Mu-like*	*08*	*Haemophilus ducreyi*	GHA2	2	GCF_001647835.1_ASM164783v1	34.1	intact	150	48	1326674-1360788	Escher_D108_NC_013594	42.74%
H194	1	*Transposable*	*Mu-like*	*09*	*Haemophilus ducreyi*	GHA3	1	GCF_001647855.1_ASM164785v1	35.8	intact	140	53	30543-66347	Escher_D108_NC_013594	47.54%
H194	2	*Transposable*	*SuMu-like*	*05*	*Haemophilus ducreyi*	GHA3	1	GCF_001647855.1_ASM164785v1	35.9	intact	150	50	397144-433048	Mannhe_vB_MhM_3927AP2_NC_02876	42.27%
H195	1	*Transposable*	*Mu-like*	*09*	*Haemophilus ducreyi*	GHA5	1	GCF_001647875.1_ASM164787v1	35.8	intact	140	53	30186-65990	Escher_D108_NC_013594	47.54%
H195	2	*Transposable*	*SuMu-like*	*05*	*Haemophilus ducreyi*	GHA5	1	GCF_001647875.1_ASM164787v1	35.9	intact	150	50	1401983-1437887	Mannhe_vB_MhM_3927AP2_NC_02876	42.27%
H196	1	*Transposable*	*Mu-like*	*09*	*Haemophilus ducreyi*	GHA8	1	GCF_001647895.1_ASM164789v1	35.6	intact	150	56	209682-245343	Escher_D108_NC_013594	47.51%
H196	2	*Transposable*	*SuMu-like*	*05*	*Haemophilus ducreyi*	GHA8	1	GCF_001647895.1_ASM164789v1	38.6	intact	150	52	576746-615397	Mannhe_vB_MhM_3927AP2_NC_02876	41.90%
H196	3	*Transposable*	*B3-like*	*12*	*Haemophilus ducreyi*	GHA8	1	GCF_001647895.1_ASM164789v1	37	intact	150	51	935338-972356	Pseudo_vB_PaeS_PM105_NC_028667	41.53%
H197	1	*Transposable*	*Mu-like*	*09*	*Haemophilus ducreyi*	GHA9	1	GCF_001647915.1_ASM164791v1	35.6	intact	150	56	604008-639669	Escher_D108_NC_013594	47.51%
H197	2	*Transposable*	*SuMu-like*	*05*	*Haemophilus ducreyi*	GHA9	1	GCF_001647915.1_ASM164791v1	39.1	intact	150	52	971072-1010201	Mannhe_vB_MhM_3927AP2_NC_02876	41.72%
H197	3	*Transposable*	*B3-like*	*12*	*Haemophilus ducreyi*	GHA9	1	GCF_001647915.1_ASM164791v1	36.5	intact	150	51	1330139-1366679	Pseudo_vB_PaeS_PM105_NC_028667	41.72%
H198	1				*Haemophilus aegyptius*	CCUG 628		GCF_001679305.1_ASM167930v1	15.4	intact	150	19	27787-43232	Mannhe_vB_MhM_587AP1_NC_028898	40.98%
H198	2				*Haemophilus aegyptius*	CCUG 628		GCF_001679305.1_ASM167930v1	20.9	incomplete	60	34	11449-32362	Mannhe_vB_MhM_3927AP2_NC_02876	42.05%
H198	3				*Haemophilus aegyptius*	CCUG 628		GCF_001679305.1_ASM167930v1	12.2	questionable	80	22	1-12246	Mannhe_vB_MhM_587AP1_NC_028898	40.26%
H198	4				*Haemophilus aegyptius*	CCUG 628		GCF_001679305.1_ASM167930v1	7.3	incomplete	60	9	59257-66613	Mannhe_vB_MhM_3927AP2_NC_02876	36.10%
H198	5	*Lambda*	*Aaphi23-like*	*24*	*Haemophilus aegyptius*	CCUG 628		GCF_001679305.1_ASM167930v1	50.5	intact	150	72	822-51348	Acinet_LZ35_NC_031117	39.69%
H199	1	*P1*	*MHaA1-like*	*19*	*Haemophilus aegyptius*	CCUG 26840		GCF_001679335.1_ASM167933v1	35.3	intact	150	57	332-35672	Mannhe_vB_MhM_587AP1_NC_028898	40.68%

TABLE S2

Strain ID	Region	Type	Supercluster	Cluster	Species	Strain	Clade	Refseq/Genbank	Region Length	Completeness	Score	# Total Proteins	Region Position	Most Common Phage	GC %
H199	2				*Haemophilus aegyptius*	CCUG 26840		GCF_001679335.1_ASM167933v1	6.2	incomplete	60	12	1-6252	Haemop_SuMu_NC_019455	40.67%
H199	3				*Haemophilus aegyptius*	CCUG 26840		GCF_001679335.1_ASM167933v1	18.8	questionable	70	22	71-18963	Haemop_SuMu_NC_019455	41.06%
H199	4				*Haemophilus aegyptius*	CCUG 26840		GCF_001679335.1_ASM167933v1	13.7	incomplete	30	23	34-13737	Pseudo_phi2_NC_030931	40.40%
H199	5	*Lambda*	*Aaphi23-like*	*24*	*Haemophilus aegyptius*	CCUG 26840		GCF_001679335.1_ASM167933v1	34.4	intact	100	53	750-35199	Aggreg_S1249_NC_013597	39.99%
H199	6				*Haemophilus aegyptius*	CCUG 26840		GCF_001679335.1_ASM167933v1	14.2	questionable	70	20	239-14450	Haemop_SuMu_NC_019455	43.34%
H199	7				*Haemophilus aegyptius*	CCUG 26840		GCF_001679335.1_ASM167933v1	21.5	questionable	70	31	857-22368	Erwini_phiEt88_NC_015295	42.06%
H199	8				*Haemophilus aegyptius*	CCUG 26840		GCF_001679335.1_ASM167933v1	16.8	incomplete	30	18	119654-136488	Bacill_BCD7_NC_019515	38.49%
H199	9				*Haemophilus aegyptius*	CCUG 26840		GCF_001679335.1_ASM167933v1	17.2	incomplete	30	12	234403-251690	Pseudo_phi2_NC_030931	36.53%
H200	1				*Haemophilus parainfluenzae*	CCUG 66565		GCF_001679495.1_ASM167949v1	15.1	intact	110	22	67870-82981	Shigel_SfIV_NC_022749	37.69%
H200	2	*hybrid*	*1152AP2-like*	*27*	*Haemophilus parainfluenzae*	CCUG 66565		GCF_001679495.1_ASM167949v1	98.1	intact	150	130	138126-236260	Mannhe_vB_MhS_1152AP2_NC_028956	40.92%
H201	1	*Lambda*	*Aaphi23-like*	*24*	*Haemophilus parainfluenzae*	CCUG 60358		GCF_001679485.1_ASM167948v1	48.2	intact	120	77	32-48247	Acinet_LZ35_NC_031117	40.25%
H201	2	*P1*	*HP1-like*	*22*	*Haemophilus parainfluenzae*	CCUG 60358		GCF_001679485.1_ASM167948v1	40.2	intact	141	58	218785-259063	Haemop_HP2_NC_003315	39.75%
H201	3				*Haemophilus parainfluenzae*	CCUG 60358		GCF_001679485.1_ASM167948v1	29.1	questionable	70	23	171943-201082	Entero_P4_NC_001609	37.92%
H202	1	*P1*	*MHaA1-like*	*19*	*Haemophilus haemolyticus*	CCUG 24149		GCF_001679135.1_ASM167913v1	32.7	intact	150	52	381-33131	Mannhe_vB_MhM_587AP1_NC_028898	40.73%
H203	1	*Transposable*	*SuMu-like*	*01*	*Haemophilus haemolyticus*	CCUG 12834		GCF_001679045.1_ASM167904v1	56.8	intact	150	56	260826-317685	Haemop_SuMu_NC_019455	41.08%
H204	1				*Haemophilus haemolyticus*	CCUG 39154		GCF_001679445.1_ASM167944v1	18.3	questionable	80	25	460951-479278	Entero_SANTOR1_NC_031051	38.58%
H205	1	*Transposable*	*SuMu-like*	*02*	*Haemophilus parainfluenzae*	CCUG 62654		GCF_001679325.1_ASM167932v1	34.2	intact	150	49	3-34292	Haemop_SuMu_NC_019455	41.98%
H205	2				*Haemophilus parainfluenzae*	CCUG 62654		GCF_001679325.1_ASM167932v1	27.6	questionable	70	22	234414-262090	Salmon_ST64B_NC_004313	37.84%
H206	1				*Haemophilus parainfluenzae*	CCUG 58848		GCF_001679405.1_ASM167940v1	14.3	incomplete	60	16	280056-294451	Shigel_SfIV_NC_022749	39.39%
H207	1	*P1*	*MHaA1-like*	*20*	*Haemophilus parainfluenzae*	CCUG 62655		GCF_001679455.1_ASM167945v1	32.7	intact	150	51	321-33032	Mannhe_vB_MhM_587AP1_NC_028898	41.14%
H207	2				*Haemophilus parainfluenzae*	CCUG 62655		GCF_001679455.1_ASM167945v1	11.2	incomplete	60	18	288273-299530	Salmon_ST64B_NC_004313	38.09%
H208	1				*Haemophilus parainfluenzae*	ATCC 9796		GCF_001680775.1_ASM168077v1	25.7	questionable	80	20	222156-247936	Entero_P4_NC_001609	38.33%
H208	2	*Transposable*	*SuMu-like*	*04*	*Haemophilus parainfluenzae*	ATCC 9796		GCF_001680775.1_ASM168077v1	39.2	intact	150	54	290454-329699	Mannhe_vB_MhM_3927AP2_NC_028766	43.97%
H208	3	*P1*	*HP1-like*	*22*	*Haemophilus parainfluenzae*	ATCC 9796		GCF_001680775.1_ASM168077v1	30.9	intact	129	43	90279-121218	Haemop_HP1_NC_001697	40.58%
H209	1				*Haemophilus parainfluenzae*	HMSC061E01		GCF_001810345.1_ASM181034v1	25.6	questionable	70	19	197613-223302	Entero_P4_NC_001609	37.86%
H209	2				*Haemophilus parainfluenzae*	HMSC061E01		GCF_001810345.1_ASM181034v1	28.5	intact	150	39	328288-356812	Mannhe_vB_MhS_1152AP2_NC_028956	40.30%
H210	1	*P1*	*MHaA1-like*	*20*	*Haemophilus parainfluenzae*	HMSC066D02		GCF_001810545.1_ASM181054v1	33.1	intact	150	54	190216-223357	Mannhe_vB_MhM_587AP1_NC_028898	41.72%
H211	1	*P1*	*MHaA1-like*	*20*	*Haemophilus parainfluenzae*	HMSC066D03		GCF_001811025.1_ASM181102v1	36.8	intact	150	60	186785-223585	Mannhe_vB_MhM_587AP1_NC_028898	41.70%
H212	1				*Haemophilus influenzae*	HMSC066A11		GCF_001811665.1_ASM181166v1	14.9	incomplete	30	19	28164-43070	Lactob_PLE2_NC_031036	38.25%
H212	2				*Haemophilus influenzae*	HMSC066A11		GCF_001811665.1_ASM181166v1	11.4	incomplete	20	13	131632-143079	Salmon_SJ46_NC_031129	43.08%
H212	3				*Haemophilus influenzae*	HMSC066A11		GCF_001811665.1_ASM181166v1	23.2	questionable	70	21	2-23208	Mannhe_vB_MhS_587AP2_NC_028743	39.72%
H213	1	*Transposable*	*SuMu-like*	*01*	*Haemophilus parainfluenzae*	HMSC073C03		GCF_001814055.1_ASM181405v1	49.1	intact	150	54	5059-54165	Mannhe_vB_MhM_3927AP2_NC_028766	41.71%

TABLE S2

Strain ID	Region	Type	Supercluster	Cluster	Species	Strain	Clade	Refseq/Genbank	Region Length	Completeness	Score	# Total Proteins	Region Position	Most Common Phage	GC %
H213	2	*Lambda*	*HK97-like*	*34*	*Haemophilus parainfluenzae*	HMSC073C03		GCF_001814055.1_ASM181405v1	41.6	intact	150	65	56066-97714	Mannhe_vB_MhS_1152AP2_NC_028956	40.20%
H213	3				*Haemophilus parainfluenzae*	HMSC073C03		GCF_001814055.1_ASM181405v1	25.3	incomplete	60	20	122903-148209	Shigel_SfIV_NC_022749	37.14%
H214	1				*Haemophilus parainfluenzae*	HMSC068C11		GCF_001815355.1_ASM181535v1	25.9	questionable	80	23	125982-151919	Entero_SANTOR1_NC_031051	38.46%
H215	1				*Haemophilus influenzae*	HMSC071C11		GCF_001837655.1_ASM183765v1	16.9	intact	150	24	102533-119507	Mannhe_vB_MhM_587AP1_NC_028898	39.71%
H215	2				*Haemophilus influenzae*	HMSC071C11		GCF_001837655.1_ASM183765v1	5.9	incomplete	40	8	1274-7192	Mannhe_vB_MhM_3927AP2_NC_02876	39.28%
H216	1				*Haemophilus parainfluenzae*	HMSC61B11		GCF_001838615.1_ASM183861v1	28	questionable	70	22	129680-157695	Salmon_ST64B_NC_004313	37.83%
H217	1	*Lambda*	*HK97-like*	*35*	*Haemophilus parainfluenzae*	HMSC71H05		GCF_001838635.1_ASM183863v1	42.6	intact	150	55	316161-358835	Mannhe_vB_MhS_535AP2_NC_028853	38.85%
H218	1	*Transposable*	*B3-like*	*12*	*Haemophilus ducreyi*	ATCC 33940	2	0109315.1_IMG-taxon_2597490374_annotated_	33.1	intact	150	49	166468-199631	Pseudo_vB_PaeS_PM105_NC_028667	42.34%
H218	2	*Transposable*	*Mu-like*	*08*	*Haemophilus ducreyi*	ATCC 33940	2	0109315.1_IMG-taxon_2597490374_annotated_	31	intact	150	45	93205-124235	Escher_D108_NC_013594	43.01%
H219	1	*Transposable*	*SuMu-like*	*02*	*Haemophilus parainfluenzae*	AE-2096513		GCA_001865335.1_ASM186533v1	56.7	intact	150	84	1-56751	Haemop_SuMu_NC_019455	42.79%
H219	2				*Haemophilus parainfluenzae*	AE-2096513		GCA_001865335.1_ASM186533v1	7.2	incomplete	40	11	32803-40075	Mannhe_vB_MhS_587AP2_NC_028743	39.67%
H219	3	*Transposable*	*Mu-like*	*07*	*Haemophilus parainfluenzae*	AE-2096513		GCA_001865335.1_ASM186533v1	37.6	intact	150	50	2-37668	Escher_D108_NC_013594	44.95%
H219	4				*Haemophilus parainfluenzae*	AE-2096513		GCA_001865335.1_ASM186533v1	15.5	intact	150	20	1322-16835	Mannhe_phiMHaA1_NC_008201	41.07%
H219	5				*Haemophilus parainfluenzae*	AE-2096513		GCA_001865335.1_ASM186533v1	29.2	questionable	80	26	3-29278	Mannhe_vB_MhM_587AP1_NC_028898	39.63%
H219	6				*Haemophilus parainfluenzae*	AE-2096513		GCA_001865335.1_ASM186533v1	14.5	incomplete	50	14	15732-30242	Ralsto_RSY1_NC_025115	39.13%
H220	-				*Haemophilus parainfluenzae*	0123Y_40_43		GCA_001914055.1_ASM191405v1	-	no prophage	-	-	-	-	-
H221	1				*Haemophilus parainfluenzae*	65114 B Hi-3		GCF_001949885.1_ASM194988v1	23.3	incomplete	30	22	193751-217077	Salmon_SJ46_NC_031129	38.79%
H221	2	*Lambda*	*Gifsy2-like*	*31*	*Haemophilus parainfluenzae*	65114 B Hi-3		GCF_001949885.1_ASM194988v1	48.5	intact	150	71	53374-101923	Mannhe_vB_MhS_1152AP2_NC_028956	40.40%
H222	1				*Haemophilus parainfluenzae*	60884 B Hi-2		GCF_001949895.1_ASM194989v1	6.5	incomplete	10	11	159746-166265	Bacill_G_NC_023719	37.65%
H223	1				*Haemophilus ducreyi*	CCUG 4438	2	GCF_002015155.1_ASM201515v1	19.9	questionable	90	35	92996-112943	Escher_D108_NC_013594	41.89%
H223	2				*Haemophilus ducreyi*	CCUG 4438	2	GCF_002015155.1_ASM201515v1	21.9	intact	120	39	312-22296	Pseudo_vB_PaeS_PM105_NC_028667	41.03%
H224	1				*Haemophilus parahaemolyticus*	CCUG 3716		GCF_002015035.1_ASM201503v1	20.8	incomplete	20	15	326264-347148	Acinet_LZ35_NC_031117	40.78%
H224	2	*Lambda*	*1152AP2-like*	*28*	*Haemophilus parahaemolyticus*	CCUG 3716		GCF_002015035.1_ASM201503v1	28.2	intact	120	50	339617-367852	Mannhe_vB_MhS_1152AP2_NC_028956	41.74%
H224	3				*Haemophilus parahaemolyticus*	CCUG 3716		GCF_002015035.1_ASM201503v1	26.5	intact	110	36	514213-540784	Haemop_HP2_NC_003315	44.50%
H225	1				*Haemophilus paraphrohaemolyticus*	CCUG 3718		GCF_002015045.1_ASM201504v1	9.5	incomplete	20	15	370792-380294	Escher_D108_NC_013594	41.40%
H225	2				*Haemophilus paraphrohaemolyticus*	CCUG 3718		GCF_002015045.1_ASM201504v1	8.6	incomplete	50	13	11919-20570	Erwini_vB_EamM_Phobos_NC_031043	39.69%
H225	3	*Lambda*	*1152AP2-like*	*28*	*Haemophilus paraphrohaemolyticus*	CCUG 3718		GCF_002015045.1_ASM201504v1	35.2	intact	130	62	655-35892	Mannhe_vB_MhS_1152AP2_NC_028956	41.74%
H225	4	*Transposable*	*BcepMu-like*	*18*	*Haemophilus paraphrohaemolyticus*	CCUG 3718		GCF_002015045.1_ASM201504v1	39.9	intact	100	63	2115-42088	Burkho_BcepMu_NC_005882	44.12%
H225	5	*Lambda*	*Gifsy2-like*	*31*	*Haemophilus paraphrohaemolyticus*	CCUG 3718		GCF_002015045.1_ASM201504v1	29.4	intact	150	46	3612-33077	Mannhe_vB_MhS_1152AP2_NC_028956	41.13%

Table S2. Phage-like elements detected in genomes of *Aggregatibacter* and *Haemophilus* species.

Table consists of phage-like elements listed in rows and their characteristics in columns. The following information was provided for phage-like element: host ID; region number, phage group; phage supercluster; phage cluster, host species taxonomy; strain, clade; Refseq genome accession number, region length, completeness; PHASTER score, number of detected ORFs, region position, most related phage predicted based on text mining of blast result [phage that has the highest number of homologs (e < 104) shared with subject phage]; and GC content.

TABLE S3

Phage ID	Supercluster	Cluster	IMG/VR singleton/cluster	Scaffold ID	Ecosystem Subtype	Host	Host detection	Estimated completeness	Quality	Predicted genome size (bp)
X001	Aaphi23	41	vOTU_002504	pt300_5760305	Oral	*Haemophilus parainfluenzae;* unclassified	CRISPR-spacer	91.46	High-quality draft genome	43811
X002	unknown 1	46	vOTU_018967	Ga0099391_100235	Oral	*Haemophilus parainfluenzae*	CRISPR-spacer	90.79	High-quality draft genome	57828
X003	HK97	35	vOTU_000059	Ga0099614_100337	Oral	*Bibersteinia trehalosi; Haemophilus parainfluenzae; Haemophilus sputorum; Mannheimia succiniciproducens; Pasteurella bettyae;* unclassified	CRISPR-spacer(ext)	99	High-quality draft genome	38979
X004	MHaA1	38	vOTU_011148	Ga0099622_100194	Oral	*Aggregatibacter* sp.oral taxon 458	CRISPR-spacer	99	High-quality draft genome	37355
X005	Gifsy-2	43	vOTU_002697	Ga0099657_100025	Oral	*Aggregatibacter actinomycetemcomitans*	CRISPR-spacer	97.35	High-quality draft genome	45633
X006	HK97	44	vOTU_005916	Ga0099581_100223	Oral	*Haemophilus parainfluenzae;* unclassified	CRISPR-spacer	99	High-quality draft genome	46249
X007	Aaphi23	41	vOTU_019100	Ga0100222_100180	Oral	*Haemophilus parainfluenzae;* unclassified	CRISPR-spacer	98.65	High-quality draft genome	43811
X008	HP1	21	vOTU_014390	Ga0100254_100009	Oral	*Haemophilus haemolyticus; Pasteurella multocida*	CRISPR-spacer	99	High-quality draft genome	32292
X009	S13	52	vOTU_000055	Ga0101032_100001	Oral	*Aggregatibacter* sp. oral taxon 458; *Haemophilus haemolyticus*	CRISPR-spacer	100	High-quality draft genome	151593
X010	phiKZ	47	vOTU_002372	Ga0101078_100001	Oral	*Haemophilus haemolyticus*	CRISPR-spacer	100	High-quality draft genome	278993
X011	HK97	35	vOTU_000059	Ga0100365_1000143	Oral	*Haemophilus parainfluenzae;* unclassified	CRISPR-spacer	99	High-quality draft genome	38979
X012	HK97	35	vOTU_000059	Ga0100058_100308	Oral	*Haemophilus parainfluenzae; Pasteurella bettyae;* unclassified	CRISPR-spacer	99	High-quality draft genome	38979
X013	HP1	22	vOTU_000492	Ga0101795_100190	Oral	*Haemophilus haemolyticus; Haemophilus parainfluenzae; Pasteurella multocida*	CRISPR-spacer	99	High-quality draft genome	32292
X014	SuMu	2	vOTU_003580	Ga0101797_1000051	Oral	*Aggregatibacter segnis; Haemophilus parainfluenzae*	CRISPR-spacer	97.46	High-quality draft genome	38770
X015	HK97	35	vOTU_000059	Ga0101799_100102	Oral	*Haemophilus parainfluenzae;* unclassified	CRISPR-spacer	99	High-quality draft genome	38979
X016	MHaA1	20	vOTU_040582	Ga0101800_100211	Oral	*Haemophilus parainfluenzae*	CRISPR-spacer	99	High-quality draft genome	37355
X017	unknown 1	46	vOTU_015010	Ga0099611_100089	Oral	*Haemophilus parainfluenzae;* unclassified	CRISPR-spacer	99	High-quality draft genome	48351
X018	HK97	35	vOTU_000059	Ga0102632_1000137	Oral	*Haemophilus parainfluenzae; Pasteurella bettyae;* unclassified	CRISPR-spacer	99	High-quality draft genome	38979
X019	HP1	39	vOTU_044378	Ga0102640_1000326	Oral	*Aggregatibacter segnis; Mannheimia succiniciproducens*	CRISPR-spacer	99	High-quality draft genome	32292
X020	HK97	35	vOTU_000059	Ga0102685_100288	Oral	*Haemophilus parainfluenzae;* unclassified	CRISPR-spacer	99	High-quality draft genome	38979
X021	HK97	35	vOTU_000059	Ga0104793_100377	Oral	*Bibersteinia trehalosi; Haemophilus parainfluenzae; Haemophilus sputorum; Mannheimia succiniciproducens; Pasteurella bettyae;* unclassified	CRISPR-spacer(ext)	99	High-quality draft genome	38979
X022	MHaA1	20	vOTU_015183	Ga0104928_100008	Oral	*Haemophilus parainfluenzae*	CRISPR-spacer	99	High-quality draft genome	35904
X023	MHaA1	20	vOTU_083580	Ga0104968_100296	Oral	*Haemophilus parainfluenzae*	CRISPR-spacer	93.47	High-quality draft genome	37355
X024	unknown 1	46	vOTU_015010	Ga0104922_100183	Oral	*Haemophilus parainfluenzae*	CRISPR-spacer	99	High-quality draft genome	48351
X025	HK97	35	vOTU_000059	Ga0104925_100103	Oral	*Haemophilus parainfluenzae*	CRISPR-spacer	96.9	High-quality draft genome	38979
X026	SuMu	2	vOTU_011197	Ga0104925_100109	Oral	*Haemophilus parainfluenzae;* unclassified	CRISPR-spacer	95.98	High-quality draft genome	37355
X027	Gifsy-2	31	vOTU_003585	Ga0104979_100026	Oral	*Haemophilus haemolyticus*	CRISPR-spacer	95.46	High-quality draft genome	44045
X028	HP1	40	sg_285414	Ga0105530_1000349	Oral	*Aggregatibacter segnis; Aggregatibacter* sp.oral taxon 458	CRISPR-spacer	99	High-quality draft genome	32292
X029	MHaA1	19	vOTU_002366	Ga0105537_100069	Oral	*Haemophilus haemolyticus; Haemophilus parainfluenzae*	CRISPR-spacer	98.68	High-quality draft genome	33480
X030	HK97	35	vOTU_009967	Ga0105680_100219	Oral	*Haemophilus sputorum; Pasteurella bettyae;* unclassified	CRISPR-spacer	94.51	High-quality draft genome	45440
X031	phiKZ	49	vOTU_009403	Ga0105762_100002	Oral	*Haemophilus haemolyticus*	CRISPR-spacer	90.07	High-quality draft genome	241426
X032	HP1	21	vOTU_014390	Ga0105762_100039	Oral	*Haemophilus haemolyticus*	CRISPR-spacer	99	High-quality draft genome	32292
X033	1152AP2	27	vOTU_020358	Ga0105779_100021	Oral	*Aggregatibacter actinomycetemcomitans; Aggregatibacter segnis*	CRISPR-spacer	99	High-quality draft genome	43811
X034	phiKZ	48	vOTU_004936	Ga0105780_100004	Oral	*Haemophilus sputorum*	CRISPR-spacer(ext)	99	High-quality draft genome	241426
X035	phiKZ	49	vOTU_086883	Ga0111236_100001	Oral	*Haemophilus haemolyticus*	CRISPR-spacer	100	High-quality draft genome	220461
X036back	HK97	33	vOTU_003656	Ga0111236_100007	Oral	*Haemophilus massiliensis; Haemophilus parainfluenzae; Mannheimia succiniciproducens*	CRISPR-spacer	99	High-quality draft genome	37736
X036front	BcepMu	17	~~vOTU_003656~~	Ga0111236_100007	Oral	*Haemophilus massiliensis; Haemophilus parainfluenzae; Mannheimia succiniciproducens*	CRISPR-spacer	99	High-quality draft genome	37736
X037	MHaA1	19	vOTU_002366	Ga0111236_100023	Oral	*Haemophilus haemolyticus; Haemophilus parainfluenzae*	CRISPR-spacer	99	High-quality draft genome	33480
X038	phiKZ	47	vOTU_019167	Ga0113985_100001	Oral	*Haemophilus haemolyticus*	CRISPR-spacer	100	High-quality draft genome	278782
X039	phiKZ	47	vOTU_019167	Ga0113995_100001	Oral	*Haemophilus haemolyticus*	CRISPR-spacer	100	High-quality draft genome	278782
X040	MHaA1	19	vOTU_002366	Ga0105958_100013	Oral	*Haemophilus haemolyticus; Haemophilus parainfluenzae*	CRISPR-spacer	100	High-quality draft genome	33480
X041	HK97	35	vOTU_002859	Ga0105969_100150	Oral	*Haemophilus parainfluenzae; Haemophilus sputorum; Mannheimia succiniciproducens*	CRISPR-spacer	92.87	High-quality draft genome	46174
X042	S13	52	vOTU_000055	Ga0114366_1000005	Oral	*Aggregatibacter* sp. oral taxon 458	CRISPR-spacer	94.13	High-quality draft genome	151638
X043	HP1	22	vOTU_000492	Ga0114366_1000201	Oral	*Haemophilus haemolyticus*	CRISPR-spacer	94.77	High-quality draft genome	32292
X044	unknown 1	46	vOTU_046685	Ga0105957_100227	Oral	*Haemophilus parainfluenzae*	CRISPR-spacer	99	High-quality draft genome	48351
X045	HK97	35	vOTU_000059	Ga0105957_100367	Oral	*Haemophilus parainfluenzae*	CRISPR-spacer	99.09	High-quality draft genome	38979
X046	587AP2	26	vOTU_008009	Ga0114309_100180	Oral	*Aggregatibacter actinomycetemcomitans; Haemophilus parainfluenzae*	CRISPR-spacer	98.91	High-quality draft genome	49151
X047	HK97	35	vOTU_000059	Ga0114309_100207	Oral	*Haemophilus parainfluenzae;* unclassified	CRISPR-spacer	99	High-quality draft genome	38979
X048	HP1	22	vOTU_046698	Ga0114309_100358	Oral	*Haemophilus haemolyticus*	CRISPR-spacer	96.5	High-quality draft genome	32292
X049	Aaphi23	41	vOTU_087519	Ga0114261_100110	Oral	*Aggregatibacter actinomycetemcomitans; Pasteurella bettyae;* unclassified	CRISPR-spacer	99	High-quality draft genome	43811
X050	unknown 1	46	vOTU_018967	Ga0114854_100189	Oral	*Haemophilus parainfluenzae*	CRISPR-spacer	90.91	High-quality draft genome	57828
X051	out	out	vOTU_020837	Ga0114846_100003	Oral	*Haemophilus haemolyticus*	CRISPR-spacer	99	High-quality draft genome	62639
X052	MHaA1	20	sg_286480	Ga0114850_100421	Oral	*Haemophilus haemolyticus;* unclassified	CRISPR-spacer	99	High-quality draft genome	35904
X053	HP1	22	vOTU_005379	Ga0111464_100142	Oral	*Haemophilus haemolyticus; Haemophilus parainfluenzae; Pasteurella multocida*	CRISPR-spacer(ext)	99	High-quality draft genome	32292
X054	MHaA1	38	vOTU_046753	Ga0111464_100318	Oral	*Aggregatibacter* sp. oral taxon 458	CRISPR-spacer	92.15	High-quality draft genome	37355
X055	S13	52	vOTU_000055	Ga0114843_100016	Oral	*Aggregatibacter* sp. oral taxon 458	CRISPR-spacer	100	High-quality draft genome	150879
X056	Aaphi23	41	vOTU_002504	Ga0114262_100206	Oral	*Haemophilus parainfluenzae*	CRISPR-spacer	96.36	High-quality draft genome	43811
X057	PA73	45	vOTU_088449	Ga0114263_1000108	Oral	*Aggregatibacter actinomycetemcomitans; Haemophilus parainfluenzae*	CRISPR-spacer	100	High-quality draft genome	48368
X058	HK97	35	vOTU_000059	Ga0114267_100093	Oral	*Bibersteinia trehalosi; Haemophilus parainfluenzae*	CRISPR-spacer	99	High-quality draft genome	38979
X059	Gifsy-2	43	vOTU_088481	Ga0110914_100242	Oral	*Haemophilus parainfluenzae;* unclassified	CRISPR-spacer	99	High-quality draft genome	43811
X060	HK97	35	vOTU_009967	Ga0115409_100105	Oral	*Haemophilus sputorum; Pasteurella bettyae*	CRISPR-spacer	99	High-quality draft genome	45440
X061	SuMu	4	vOTU_020273	Ga0115221_1000069	Oral	*Aggregatibacter* sp. oral taxon 458; *Haemophilus parainfluenzae*	CRISPR-spacer	99	High-quality draft genome	37355
X062	PA73	45	vOTU_047184	Ga0115224_1000288	Oral	*Aggregatibacter actinomycetemcomitans; Haemophilus parainfluenzae;* unclassified	CRISPR-spacer	99	High-quality draft genome	48368
X063	HP1	22	vOTU_047192	Ga0115225_100119	Oral	*Haemophilus haemolyticus; Haemophilus parainfluenzae*	CRISPR-spacer	99	High-quality draft genome	32292
X064	phiKZ	48	vOTU_011118	Ga0115418_100017	Oral	*Haemophilus sputorum*	CRISPR-spacer	100	High-quality draft genome	254212
X065	HP1	22	vOTU_000492	Ga0105906_1000189	Oral	*Haemophilus haemolyticus; Haemophilus parainfluenzae; Pasteurella multocida*	CRISPR-spacer(ext)	99	High-quality draft genome	32292
X066	MHaA1	20	vOTU_028844	Ga0111013_100131	Oral	*Haemophilus parainfluenzae;* unclassified	CRISPR-spacer	92.62	High-quality draft genome	37355
X067	SuMu	37	sg_288269	Ga0115176_1000470	Oral	*Aggregatibacter actinomycetemcomitans*	CRISPR-spacer	95.81	High-quality draft genome	39049
X068	HP1	22	vOTU_000492	Ga0111023_100286	Oral	*Haemophilus haemolyticus; Haemophilus parainfluenzae; Pasteurella multocida*	CRISPR-spacer(ext)	99	High-quality draft genome	32292
X069	Gifsy-2	43	vOTU_047283	Ga0115191_100285	Oral	*Haemophilus parainfluenzae;* unclassified	CRISPR-spacer	96.86	High-quality draft genome	43811
X070	MHaA1	19	vOTU_046807	Ga0111061_1000136	Oral	*Haemophilus haemolyticus; Haemophilus parainfluenzae*	CRISPR-spacer	99	High-quality draft genome	37355
X071	HP1	21	vOTU_015434	Ga0111234_100051	Oral	*Haemophilus haemolyticus*	CRISPR-spacer	99	High-quality draft genome	32292
X072	HP1	22	vOTU_005379	Ga0111366_100212	Oral	*Haemophilus parainfluenzae*	CRISPR-spacer	94.21	High-quality draft genome	32292
X073	phiKZ	50	vOTU_007652	Ga0111423_100002	Oral	*Haemophilus parahaemolyticus*	CRISPR-spacer	100	High-quality draft genome	211077
X074	unknown 2	51	vOTU_009501	Ga0111423_100005	Oral	*Aggregatibacter* sp. oral taxon 458; *Haemophilus haemolyticus*	CRISPR-spacer	92.52	High-quality draft genome	189719
X075	HP1	22	vOTU_000492	Ga0111423_100080	Oral	*Haemophilus haemolyticus*	CRISPR-spacer	90.88	High-quality draft genome	32292
X076	S13	52	vOTU_000055	Ga0113871_100006	Oral	*Aggregatibacter* sp. oral taxon 458; *Haemophilus haemolyticus*	CRISPR-spacer	100	High-quality draft genome	151614
X077	Gifsy-2	31	vOTU_003585	Ga0115608_100110	Oral	*Haemophilus haemolyticus*	CRISPR-spacer(ext)	96.28	High-quality draft genome	44045
X078	S13	52	vOTU_000055	Ga0115616_100001	Oral	*Aggregatibacter* sp. oral taxon 458; *Haemophilus haemolyticus*	CRISPR-spacer	100	High-quality draft genome	151638
X079	Aaphi23	41	vOTU_002504	Ga0114089_100276	Oral	*Haemophilus parainfluenzae;* unclassified	CRISPR-spacer	99	High-quality draft genome	43811
X080	HP1	22	vOTU_000492	Ga0134350_1000121	Oral	*Haemophilus parainfluenzae*	CRISPR-spacer	100	High-quality draft genome	30671
X081	Gifsy-2	42	sg_279256	Ga0169799_10052	Large intestine	*Haemophilus haemolyticus*	CRISPR-spacer	99	High-quality draft genome	46213
X082	HK97	35	vOTU_000059	Ga0169793_100334	Large intestine	*Haemophilus parainfluenzae*	CRISPR-spacer	99	High-quality draft genome	38979
X083	1152AP2	27	vOTU_020358	SRS022532_LANL_scaffold_5284	Oral	*Aggregatibacter actinomycetemcomitans; Aggregatibacter segnis; Haemophilus haemolyticus*	CRISPR-spacer	99	High-quality draft genome	43811
X084	unknown 1	46	vOTU_002371	SRS022536_LANL_scaffold_98123	Oral	*Haemophilus haemolyticus; Haemophilus parainfluenzae*	CRISPR-spacer/prophage	93.45	High-quality draft genome	61805
X085	HK97	35	vOTU_000059	SRS013705_Baylor_scaffold_102851	Oral	*Haemophilus parainfluenzae*	CRISPR-spacer	99	High-quality draft genome	38979
X086	Aaphi23	41	vOTU_028887	SRS064774_LANL_scaffold_17042	Oral	*Haemophilus parainfluenzae;* unclassified	CRISPR-spacer	99.06	High-quality draft genome	43811
X087	HP1	22	vOTU_000492	SRS017304_Baylor_scaffold_3664	Oral	*Haemophilus haemolyticus; Haemophilus parainfluenzae; Pasteurella multocida*	CRISPR-spacer	95.37	High-quality draft genome	32292
X088	HP1	22	vOTU_000492	SRS011098_Baylor_scaffold_28115	Oral	*Haemophilus haemolyticus; Haemophilus parainfluenzae*	CRISPR-spacer	97.33	High-quality draft genome	32292
X089	HP1	39	vOTU_044378	C4144148	Oral	*Aggregatibacter segnis; Mannheimia succiniciproducens*	CRISPR-spacer	97.98	High-quality draft genome	32292
X090	HP1	22	vOTU_000492	SRS012285_Baylor_scaffold_64538	Oral	*Haemophilus haemolyticus; Haemophilus parainfluenzae; Pasteurella multocida*	CRISPR-spacer	97.3	High-quality draft genome	32292
X091	Gifsy-2	43	vOTU_033146	SRS014690_WUGC_scaffold_24896	Oral	*Haemophilus parainfluenzae;* unclassified	CRISPR-spacer	91.33	High-quality draft genome	44045
X092	unknown 1	46	vOTU_006131	SRS013881_WUGC_scaffold_945	Oral	*Haemophilus parainfluenzae;* unclassified	CRISPR-spacer	99	High-quality draft genome	57828
X093	HP1	39	vOTU_044378	SRS014894_WUGC_scaffold_63344	Oral	*Aggregatibacter segnis*	CRISPR-spacer	94.63	High-quality draft genome	32292
X094	MHaA1	20	sg_291962	SRS015378_WUGC_scaffold_9976	Oral	*Haemophilus parainfluenzae*	CRISPR-spacer	90.71	High-quality draft genome	37355
X095	HK97	35	vOTU_000059	SRS022077_Baylor_scaffold_21509	Oral	*Bibersteinia trehalosi; Haemophilus parainfluenzae; Haemophilus sputorum; Mannheimia succiniciproducens; Pasteurella bettyae;* unclassified	CRISPR-spacer(ext)	100	High-quality draft genome	38979
X096	Aaphi23	41	vOTU_083578	SRS057539_LANL_scaffold_23351	Oral	*Haemophilus parainfluenzae;* unclassified	CRISPR-spacer	92.27	High-quality draft genome	45511
X097	HP1	40	sg_292341	SRS063999_LANL_scaffold_85949	Oral	*Aggregatibacter segnis; Aggregatibacter* sp.oral taxon 458	CRISPR-spacer	99	High-quality draft genome	32292
X098	unknown 1	46	vOTU_002371	SRS015921_WUGC_scaffold_307	Oral	*Haemophilus haemolyticus; Haemophilus parainfluenzae*	CRISPR-spacer/prophage	99	High-quality draft genome	7160
X099	HP1	22	vOTU_000492	SRS020856_Baylor_scaffold_11049	Oral	*Haemophilus parainfluenzae*	CRISPR-spacer	96.16	High-quality draft genome	32292
X100	MHaA1	19	vOTU_002366	SRS015646_WUGC_scaffold_1752	Oral	*Haemophilus haemolyticus; Haemophilus parainfluenzae*	CRISPR-spacer	97.92	High-quality draft genome	33480
X101	HK97	33	vOTU_003656	SRS015646_WUGC_scaffold_4249	Oral	*Haemophilus massiliensis; Haemophilus parainfluenzae; Mannheimia succiniciproducens*	CRISPR-spacer	99	High-quality draft genome	37736
X102	HK97	35	vOTU_000059	SRS012279_Baylor_scaffold_64360	Oral	*Bibersteinia trehalosi; Haemophilus parainfluenzae; Haemophilus sputorum; Mannheimia succiniciproducens; Pasteurella bettyae;* unclassified	CRISPR-spacer(ext)	100	High-quality draft genome	36994
X103	phiKZ	47	vOTU_019167	SRS016039_WUGC_scaffold_7258	Oral	*Haemophilus haemolyticus*	CRISPR-spacer	100	High-quality draft genome	278729
X104	Aaphi23	41	vOTU_004206	SRS049318_LANL_scaffold_12279	Oral	*Aggregatibacter actinomycetemcomitans*	CRISPR-spacer	90.56	High-quality draft genome	45440
X105	HP1	22	vOTU_000492	SRS024144_LANL_scaffold_27383	Oral	*Haemophilus haemolyticus; Haemophilus parainfluenzae*	CRISPR-spacer	94.15	High-quality draft genome	32292
X106	Gifsy-2	43	vOTU_033146	SRS019028_WUGC_scaffold_3808	Oral	*Haemophilus parainfluenzae;* unclassified	CRISPR-spacer	92.18	High-quality draft genome	44045
X107	HP1	22	vOTU_000492	SRS019980_Baylor_scaffold_61046	Oral	*Haemophilus parainfluenzae*	CRISPR-spacer	95.04	High-quality draft genome	32292
X108	HP1	40	sg_295118	SRS023938_Baylor_scaffold_39349	Oral	*Aggregatibacter segnis; Aggregatibacter* sp.oral taxon 458	CRISPR-spacer	99	High-quality draft genome	32292

Table S3. Classification of viral assemblies from metagenomes

Table consists of viral assemblies listed in rows and their characteristics in columns. The following information was provided: phage ID; supercluster classification, cluster classification (all three from this study), IMG/VR singleton/cluster, Scaffold ID, Ecosystem Subtype, Host, Host detection, Estimated completeness, Quality, and Predicted genome size (all retrieved from IMG/VR) (9-12).

TABLE S4

Species	Cluster 10	Cluster 13	Cluster 25	HK No.	SPS No.	Other names	Serotype	Isolated from	Country	Source
Aggregatibacter actinomycetemcomitans	no	yes	no		9	ATCC 29524, CCUG 1210, CDC B5386, DSM 11122, NCTC 10982	b	Abscess	USA	R. Mutters (Marburg, Germany)
Aggregatibacter actinomycetemcomitans	no	no	no		32	MCCM 02325	b	Unknown		R. Mutters (Marburg, Germany)
Aggregatibacter actinomycetemcomitans	no	no	yes		33	Type strain, ATCC 33384, CCUG 13227, DSM 8324, NCTC 9710, HG683, 277/49-50(8), 277/49-50, 3rd batch	c	Abscess	Denmark	R. Mutters (Marburg, Germany)
Aggregatibacter actinomycetemcomitans	no	no	no		69	ATCC 43718, DSM 11123, FDC Y4	b	Dental plaque	USA	R. Mutters (Marburg, Germany)
Aggregatibacter actinomycetemcomitans	no	yes	no	921	70	CCUG 56172, JP2, 2nd batch	b	Dental plaque	USA	R. Mutters (Marburg, Germany)
Aggregatibacter actinomycetemcomitans	no	no	yes		71	21 C, MCCM 00135	a	Unknown	Germany	R. Mutters (Marburg, Germany)
Aggregatibacter actinomycetemcomitans	yes	no	no		72	ATCC 43717; MCCM 02178; HIM 1039-9; Reynolds SUNYaB 75	a	Dental plaque	USA	R. Mutters (Marburg, Germany)
Aggregatibacter actinomycetemcomitans	yes	no	no		73	M.Baum 24, MCCM 03098	a	Adult periodontitis	Germany	R. Mutters (Marburg, Germany)
Aggregatibacter actinomycetemcomitans	yes	no	no		74	M.Baum Ki 1, MCCM 03163	a	Dental plaque	Germany	R. Mutters (Marburg, Germany)
Aggregatibacter actinomycetemcomitans	no	no	no		75	CCUG 38565, OMZ 542, IDH 781	d	Unknown	Finland	R. Mutters (Marburg, Germany)
Aggregatibacter actinomycetemcomitans	no	no	no		76	MCCM 02474, Stuttgart 18 / 3859	a	Unknown	Germany	R. Mutters (Marburg, Germany)
Aggregatibacter actinomycetemcomitans	no	no	no	1651	77	CCUG 56173	b	Localized juvenile periodontitis	Denmark	CCUG (Gothenburg, Sweden)
Aggregatibacter actinomycetemcomitans	no	no	no		81	CU1000N	f	Localized juvenile periodontitis	USA	H.C. Schreiner (Newark, USA)
Aggregatibacter actinomycetemcomitans	no	no	yes	1013	157	914	a	Adult periodontitis	Finland	S. Assikainen (Helsinki, Finland)
Aggregatibacter actinomycetemcomitans	no	no	yes	1014	158	1722	a	Adult periodontitis	Finland	S. Assikainen (Helsinki, Finland)
Aggregatibacter actinomycetemcomitans	no	no	yes	1015	159	2189	a	Healthy, dental plaque	Finland	S. Assikainen (Helsinki, Finland)
Aggregatibacter actinomycetemcomitans	no	no	yes	1016	160	2182	a	Adult periodontitis	Finland	S. Assikainen (Helsinki, Finland)
Aggregatibacter actinomycetemcomitans	no	no	yes	1017	161	511	a	Adult periodontitis	Finland	S. Assikainen (Helsinki, Finland)
Aggregatibacter actinomycetemcomitans	no	no	no	1018	162	490	a	Adult periodontitis	Finland	S. Assikainen (Helsinki, Finland)
Aggregatibacter actinomycetemcomitans	no	no	yes	1019	163	558	a	Localized juvenile periodontitis	Finland	S. Assikainen (Helsinki, Finland)
Aggregatibacter actinomycetemcomitans	no	no	yes	1020	164	849	a	Localized juvenile periodontitis	Finland	S. Assikainen (Helsinki, Finland)
Aggregatibacter actinomycetemcomitans	no	no	yes	1023	166	3812	a	Adult periodontitis	Finland	S. Assikainen (Helsinki, Finland)
Aggregatibacter actinomycetemcomitans	no	no	yes	1024	167	3135	a	Gingivitis	Finland	S. Assikainen (Helsinki, Finland)
Aggregatibacter actinomycetemcomitans	no	no	no	1026	169	2881	a	Adult periodontitis	Finland	S. Assikainen (Helsinki, Finland)
Aggregatibacter actinomycetemcomitans	no	no	no	1027	170	4157	a	Adult periodontitis	Finland	S. Assikainen (Helsinki, Finland)
Aggregatibacter actinomycetemcomitans	no	no	yes	1028	171	2690	a	Adult periodontitis	Finland	S. Assikainen (Helsinki, Finland)
Aggregatibacter actinomycetemcomitans	no	no	yes	1029	172	2489	a	Gingivitis	Finland	S. Assikainen (Helsinki, Finland)
Aggregatibacter actinomycetemcomitans	no	no	yes	1030	173	2983	a	Localized juvenile periodontitis	Finland	S. Assikainen (Helsinki, Finland)
Aggregatibacter actinomycetemcomitans	no	no	no	1031	174	3533	a	Adult periodontitis	Finland	S. Assikainen (Helsinki, Finland)
Aggregatibacter actinomycetemcomitans	no	no	yes	1032	175	3102	a	Healthy, dental plaque	Finland	S. Assikainen (Helsinki, Finland)
Aggregatibacter actinomycetemcomitans	no	no	yes	1033	176	479	a	Localized juvenile periodontitis	Finland	S. Assikainen (Helsinki, Finland)
Aggregatibacter actinomycetemcomitans	no	no	yes	1035	178	286	a	Adult periodontitis	Finland	S. Assikainen (Helsinki, Finland)
Aggregatibacter actinomycetemcomitans	no	no	no	1036	179	1398, SCC1398	b	Localized juvenile periodontitis	Finland	S. Assikainen (Helsinki, Finland)
Aggregatibacter actinomycetemcomitans	no	no	no	1040	183	3674	b	Adult periodontitis	Finland	S. Assikainen (Helsinki, Finland)
Aggregatibacter actinomycetemcomitans	no	no	yes	1041	184	2200	b	Adult periodontitis	Finland	S. Assikainen (Helsinki, Finland)
Aggregatibacter actinomycetemcomitans	no	no	no	1042	185	2257	b	Adult periodontitis	Finland	S. Assikainen (Helsinki, Finland)
Aggregatibacter actinomycetemcomitans	no	no	yes	1044	187	2502	b	Healthy, dental plaque	Finland	S. Assikainen (Helsinki, Finland)
Aggregatibacter actinomycetemcomitans	no	no	yes	1045	188	3035	b	Adult periodontitis	Finland	S. Assikainen (Helsinki, Finland)
Aggregatibacter actinomycetemcomitans	no	no	no	1046	189	2146	b	Adult periodontitis	Finland	S. Assikainen (Helsinki, Finland)
Aggregatibacter actinomycetemcomitans	yes	no	yes	1047	190	2571	b	Adult periodontitis	Finland	S. Assikainen (Helsinki, Finland)
Aggregatibacter actinomycetemcomitans	no	no	no	1048	191	670	b	Localized juvenile periodontitis	Finland	S. Assikainen (Helsinki, Finland)
Aggregatibacter actinomycetemcomitans	no	no	no	1049	192	705	b	Localized juvenile periodontitis	Finland	S. Assikainen (Helsinki, Finland)
Aggregatibacter actinomycetemcomitans	yes	no	no	1050	193	651	b	Localized juvenile periodontitis	Finland	S. Assikainen (Helsinki, Finland)
Aggregatibacter actinomycetemcomitans	no	no	no	1053	195	880	b	Adult periodontitis	Finland	S. Assikainen (Helsinki, Finland)
Aggregatibacter actinomycetemcomitans	no	no	no	1054	196	3923	b	Adult periodontitis	Finland	S. Assikainen (Helsinki, Finland)
Aggregatibacter actinomycetemcomitans	no	no	no	1055	197	724	b	Localized juvenile periodontitis	Finland	S. Assikainen (Helsinki, Finland)
Aggregatibacter actinomycetemcomitans	no	no	no	1056	198	2	c	Adult periodontitis	Finland	S. Assikainen (Helsinki, Finland)
Aggregatibacter actinomycetemcomitans	no	no	no	1057	199	2530	c	Healthy, dental plaque	Finland	S. Assikainen (Helsinki, Finland)
Aggregatibacter actinomycetemcomitans	no	no	no	1058	200	2443	c	Healthy, dental plaque	Finland	S. Assikainen (Helsinki, Finland)
Aggregatibacter actinomycetemcomitans	no	no	no	1059	201	2439	c	Healthy, dental plaque	Finland	S. Assikainen (Helsinki, Finland)
Aggregatibacter actinomycetemcomitans	no	no	no	1060	202	2631	c	Healthy, dental plaque	Finland	S. Assikainen (Helsinki, Finland)
Aggregatibacter actinomycetemcomitans	no	no	no	1061	203	2756	c	Adult periodontitis	Finland	S. Assikainen (Helsinki, Finland)
Aggregatibacter actinomycetemcomitans	no	no	yes	1062	204	4127	c	Adult periodontitis	Finland	S. Assikainen (Helsinki, Finland)
Aggregatibacter actinomycetemcomitans	no	no	no	1063	205	2302, SCC2302	c	Gingivitis	Finland	S. Assikainen (Helsinki, Finland)

TABLE S4

Species	Cluster 10	Cluster 13	Cluster 25	HK No.	SPS No.	Other names	Serotype	Isolated from	Country	Source
Aggregatibacter actinomycetemcomitans	no	no	no	1064	206	2748	c	Adult periodontitis	Finland	S. Assikainen (Helsinki, Finland)
Aggregatibacter actinomycetemcomitans	no	no	no	1066	208	5	c	Adult periodontitis	Finland	S. Assikainen (Helsinki, Finland)
Aggregatibacter actinomycetemcomitans	no	no	no	1067	209	2633	c	Adult periodontitis	Finland	S. Assikainen (Helsinki, Finland)
Aggregatibacter actinomycetemcomitans	no	no	no	1071	213	345	c	Adult periodontitis	Finland	S. Assikainen (Helsinki, Finland)
Aggregatibacter actinomycetemcomitans	no	no	no	1072	214	1194	c	Localized juvenile periodontitis	Finland	S. Assikainen (Helsinki, Finland)
Aggregatibacter actinomycetemcomitans	no	no	no	1073	215	931	c	Adult periodontitis	Finland	S. Assikainen (Helsinki, Finland)
Aggregatibacter actinomycetemcomitans	no	no	no	1074	216	301	c	Adult periodontitis	Finland	S. Assikainen (Helsinki, Finland)
Aggregatibacter actinomycetemcomitans	no	no	no	1075	217	2267	c	Localized juvenile periodontitis	Finland	S. Assikainen (Helsinki, Finland)
Aggregatibacter actinomycetemcomitans	no	no	no	1079	221	121	c	Healthy, dental plaque	Finland	S. Assikainen (Helsinki, Finland)
Aggregatibacter actinomycetemcomitans	no	no	no	1081	223	1346	d	Adult periodontitis	Finland	S. Assikainen (Helsinki, Finland)
Aggregatibacter actinomycetemcomitans	no	no	no	1082	224	7	d	Adult periodontitis	Finland	S. Assikainen (Helsinki, Finland)
Aggregatibacter actinomycetemcomitans	no	no	no	1083	225	15	d	Adult periodontitis	Finland	S. Assikainen (Helsinki, Finland)
Aggregatibacter actinomycetemcomitans	no	no	no	1084	226	508, SA508	d	Adult periodontitis	Finland	S. Assikainen (Helsinki, Finland)
Aggregatibacter actinomycetemcomitans	no	no	no	1086	228	130	d	Gingivitis	Finland	S. Assikainen (Helsinki, Finland)
Aggregatibacter actinomycetemcomitans	yes	yes	no	1087	229	13	e	Adult periodontitis	Finland	S. Assikainen (Helsinki, Finland)
Aggregatibacter actinomycetemcomitans	no	yes	no	1088	230	2876, SA2876	e	Adult periodontitis	Finland	S. Assikainen (Helsinki, Finland)
Aggregatibacter actinomycetemcomitans	no	yes	no	1089	231	2149	e	Adult periodontitis	Finland	S. Assikainen (Helsinki, Finland)
Aggregatibacter actinomycetemcomitans	no	no	no	1090	232	16	e'	Adult periodontitis	Finland	S. Assikainen (Helsinki, Finland)
Aggregatibacter actinomycetemcomitans	yes	yes	no	1092	234	9	e	Adult periodontitis	Finland	S. Assikainen (Helsinki, Finland)
Aggregatibacter actinomycetemcomitans	no	no	no	1093	235	3096, SA3096	e'	Adult periodontitis	Finland	S. Assikainen (Helsinki, Finland)
Aggregatibacter actinomycetemcomitans	no	no	yes	891	237	HG1215	a	Dental plaque	Netherlands	T.J.M. van Steenbergen (Amsterdam, Netherlands)
Aggregatibacter actinomycetemcomitans	no	no	yes	892	238	HG1230	a	Dental plaque	Netherlands	T.J.M. van Steenbergen (Amsterdam, Netherlands)
Aggregatibacter actinomycetemcomitans	no	no	yes	893	239	HG1221	a	Dental plaque	Netherlands	T.J.M. van Steenbergen (Amsterdam, Netherlands)
Aggregatibacter actinomycetemcomitans	no	no	yes	894	240	HG1224	a	Dental plaque	Netherlands	T.J.M. van Steenbergen (Amsterdam, Netherlands)
Aggregatibacter actinomycetemcomitans	no	no	yes	895	241	HG1233	a	Dental plaque	Netherlands	T.J.M. van Steenbergen (Amsterdam, Netherlands)
Aggregatibacter actinomycetemcomitans	no	no	yes	896	242	HG1234	a	Dental plaque	Netherlands	T.J.M. van Steenbergen (Amsterdam, Netherlands)
Aggregatibacter actinomycetemcomitans	no	no	yes	898	244	HG1218	a	Dental plaque	Netherlands	T.J.M. van Steenbergen (Amsterdam, Netherlands)
Aggregatibacter actinomycetemcomitans	no	no	no	902	247	HG1229, 538	b	Dental plaque	Netherlands	T.J.M. van Steenbergen (Amsterdam, Netherlands)
Aggregatibacter actinomycetemcomitans	no	no	no	903	248	HG1232	b	Dental plaque	Netherlands	T.J.M. van Steenbergen (Amsterdam, Netherlands)
Aggregatibacter actinomycetemcomitans	no	no	no	904	249	HG1220	b	Dental plaque	Netherlands	T.J.M. van Steenbergen (Amsterdam, Netherlands)
Aggregatibacter actinomycetemcomitans	no	no	no	905	250	HG1184	b	Dental plaque	Netherlands	T.J.M. van Steenbergen (Amsterdam, Netherlands)
Aggregatibacter actinomycetemcomitans	no	no	no	909	251	HG1080	b	Dental plaque	Netherlands	T.J.M. van Steenbergen (Amsterdam, Netherlands)
Aggregatibacter actinomycetemcomitans	no	no	yes	910	252	HG1182	b	Dental plaque	Netherlands	T.J.M. van Steenbergen (Amsterdam, Netherlands)
Aggregatibacter actinomycetemcomitans	no	no	no	911	253	HG1183	b	Dental plaque	Netherlands	T.J.M. van Steenbergen (Amsterdam, Netherlands)
Aggregatibacter actinomycetemcomitans	yes	no	no	913	254	HG1235	b	Dental plaque	Netherlands	T.J.M. van Steenbergen (Amsterdam, Netherlands)
Aggregatibacter actinomycetemcomitans	no	no	yes	914	255	Type strain, ATCC 33384, CCUG 13227, DSM 8324, NCTC 9710, HG683, 277/49-50(8), 277/49-50, 4th batch	c	Abscess	Denmark	T.J.M. van Steenbergen (Amsterdam, Netherlands)
Aggregatibacter actinomycetemcomitans	no	no	no	916	256	HG1226, 528	c	Dental plaque	Netherlands	T.J.M. van Steenbergen (Amsterdam, Netherlands)
Aggregatibacter actinomycetemcomitans	no	no	no	917	257	HG1227	c	Dental plaque	Netherlands	T.J.M. van Steenbergen (Amsterdam, Netherlands)
Aggregatibacter actinomycetemcomitans	no	no	no	918	258	HG1231	c	Dental plaque	Netherlands	T.J.M. van Steenbergen (Amsterdam, Netherlands)
Aggregatibacter actinomycetemcomitans	no	no	yes	919	259	HG1225	c	Dental plaque	Netherlands	T.J.M. van Steenbergen (Amsterdam, Netherlands)
Aggregatibacter actinomycetemcomitans	no	no	no	921	261	CCUG 56172, JP2, 1st batch	b	Dental plaque	USA	T. Lally (Pensylvania, USA)
Haemophilus influenzae	no	no	no	922	262	IHI 23443 (425)		Dental plaque	Finland	S. Assikainen (Helsinki, Finland)
Haemophilus influenzae	no	no	no	923	263	IHI 23814 (826)		Dental plaque	Finland	S. Assikainen (Helsinki, Finland)
Aggregatibacter actinomycetemcomitans	yes	yes	no	929	264	CCUG 51668, IDH 1705, HG3907	e	Adult periodontitis	Finland	S. Assikainen (Helsinki, Finland)
Aggregatibacter actinomycetemcomitans	no	no	no	931	265	PH 253/46	b	Unknown	Denmark	W.W. Frederiksen, P. Holm (Copenhagen, Denmark)
Aggregatibacter actinomycetemcomitans	no	no	no	932	266	PH 294/46	b	Unknown	Denmark	W.W. Frederiksen, P. Holm (Copenhagen, Denmark)
Aggregatibacter actinomycetemcomitans	no	no	no	934	268	PH 387/47, 387/47-48	c	Abscess	Denmark	W.W. Frederiksen, P. Holm (Copenhagen, Denmark)
Aggregatibacter actinomycetemcomitans	yes	yes	no	935	269	PH 406/47	c	Unknown	Denmark	W.W. Frederiksen, P. Holm (Copenhagen, Denmark)
Aggregatibacter actinomycetemcomitans	no	no	no	938	271	PH 116/48	c	Unknown	Denmark	W.W. Frederiksen, P. Holm (Copenhagen, Denmark)
Aggregatibacter actinomycetemcomitans	no	no	no	939	272	CCUG 3711, NCTC 9709, PH 120/48, 120/48-49	nt	Abscess	Denmark	W.W. Frederiksen, P. Holm (Copenhagen, Denmark)
Aggregatibacter actinomycetemcomitans	no	no	no	940	273	PH 122/48	c	Unknown	Denmark	W.W. Frederiksen, P. Holm (Copenhagen, Denmark)
Aggregatibacter actinomycetemcomitans	no	no	no	941	274	PH 284/48	c	Unknown	Denmark	W.W. Frederiksen, P. Holm (Copenhagen, Denmark)
Aggregatibacter actinomycetemcomitans	no	no	no	942	275	PH 314/48, 314/48-49	c	Abscess	Denmark	W.W. Frederiksen, P. Holm (Copenhagen, Denmark)
Aggregatibacter actinomycetemcomitans	no	no	no	943	276	PH 332/48	c	Unknown	Denmark	W.W. Frederiksen, P. Holm (Copenhagen, Denmark)

TABLE S4

Species	Cluster 10	Cluster 13	Cluster 25	HK No.	SPS No.	Other names	Serotype	Isolated from	Country	Source
Aggregatibacter actinomycetemcomitans	no	no	yes	944	277	PH 364/48	a	Unknown	Denmark	W.W. Frederiksen, P. Holm (Copenhagen, Denmark)
Aggregatibacter actinomycetemcomitans	no	no	no	945	278	PH 3/49	a	Unknown	Denmark	W.W. Frederiksen, P. Holm (Copenhagen, Denmark)
Aggregatibacter actinomycetemcomitans	no	no	yes	946	279	PH 12/49	c	Unknown	Denmark	W.W. Frederiksen, P. Holm (Copenhagen, Denmark)
Aggregatibacter actinomycetemcomitans	no	no	no	947	280	PH 52/49, 52/49-50	c	Abscess	Denmark	W.W. Frederiksen, P. Holm (Copenhagen, Denmark)
Aggregatibacter actinomycetemcomitans	no	no	yes	948	281	PH 113/49, 113/49-50	a	Abscess	Denmark	W.W. Frederiksen, P. Holm (Copenhagen, Denmark)
Aggregatibacter actinomycetemcomitans	no	no	no	950	282	PH 248/49, 248/49-50	a	Abscess	Denmark	W.W. Frederiksen, P. Holm (Copenhagen, Denmark)
Aggregatibacter actinomycetemcomitans	no	no	yes	951	283	Type strain, ATCC 33384, CCUG 13227, DSM 8324, NCTC 9710, HG683, 277/49-50(8), 277/49-50, 1st batch	c	Abscess	Denmark	W.W. Frederiksen, P. Holm (Copenhagen, Denmark)
Aggregatibacter actinomycetemcomitans	no	no	no	952	284	PH 363/49, 363/49-50	c	Abscess	Denmark	W.W. Frederiksen, P. Holm (Copenhagen, Denmark)
Aggregatibacter actinomycetemcomitans	no	no	no	953	285	PH 150/50	b	Unknown	Denmark	W.W. Frederiksen, P. Holm (Copenhagen, Denmark)
Aggregatibacter actinomycetemcomitans	no	no	no	955	286	PH 661/51	c	Unknown	Denmark	W.W. Frederiksen, P. Holm (Copenhagen, Denmark)
Aggregatibacter actinomycetemcomitans	no	no	yes	956	287	PH 336/52	a	Unknown	Denmark	W.W. Frederiksen, P. Holm (Copenhagen, Denmark)
Aggregatibacter actinomycetemcomitans	no	no	no	961	288	PH 791/56, CCUG 51669, HG3908, SSI 791/56-57	e'	Abscess	Denmark	W.W. Frederiksen, P. Holm (Copenhagen, Denmark)
Aggregatibacter actinomycetemcomitans	no	no	yes	962	289	PH 23/58, CCUG 51670, SSI 23/58-59	c	Abscess	Denmark	W.W. Frederiksen, P. Holm (Copenhagen, Denmark)
Aggregatibacter actinomycetemcomitans	yes	no	no	964	291	PHA 300/61	e'	Unknown	Denmark	W.W. Frederiksen, P. Holm (Copenhagen, Denmark)
Aggregatibacter actinomycetemcomitans	no	no	no	965	292	PHA 592/64	c	Unknown	Denmark	W.W. Frederiksen, P. Holm (Copenhagen, Denmark)
Aggregatibacter actinomycetemcomitans	no	no	no	968	293	EK 9	a	Adult periodontitis	Denmark	Own isolate (Aarhus, Denmark)
Aggregatibacter actinomycetemcomitans	no	no	no	969	294	KL 12	b	Adult periodontitis	Denmark	Own isolate (Aarhus, Denmark)
Aggregatibacter actinomycetemcomitans	no	no	no	970	295	LP 3	b	Adult periodontitis	Denmark	Own isolate (Aarhus, Denmark)
Aggregatibacter actinomycetemcomitans	no	no	no	971	296	Him 828-2	b	Unknown	Germany	W. Mannheim (Marburg, Germany)
Aggregatibacter actinomycetemcomitans	no	no	no	972	297	OMZ 295, Tsai JP2	b	Localized juvenile periodontitis	USA	B. Guggenheim (Zürich, Switzerland)
Aggregatibacter actinomycetemcomitans	no	no	no	973	298	GS 4	nt	Unknown		
Aggregatibacter actinomycetemcomitans	no	no	no	974	299	JL 291-6	a	Unknown		
Aggregatibacter actinomycetemcomitans	no	no	no	976	300	JL 1	nt	Adult periodontitis	Denmark	Own isolate (Aarhus, Denmark)
Aggregatibacter actinomycetemcomitans	no	no	no	978	301	JL 22	c	Adult periodontitis	Denmark	Own isolate (Aarhus, Denmark)
Aggregatibacter actinomycetemcomitans	no	no	no	979	302	JL 24	nt	Adult periodontitis	Denmark	Own isolate (Aarhus, Denmark)
Aggregatibacter actinomycetemcomitans	no	no	no	980	303	KA 4	b	Adult periodontitis	Denmark	Own isolate (Aarhus, Denmark)
Aggregatibacter actinomycetemcomitans	no	no	no	981	304	KE 7, CCUG 51671	c	Adult periodontitis	Denmark	Own isolate (Aarhus, Denmark)
Aggregatibacter actinomycetemcomitans	no	no	no	982	305	KK 2, CCUG 51672	b	Adult periodontitis	Denmark	Own isolate (Aarhus, Denmark)
Aggregatibacter actinomycetemcomitans	no	no	yes	983	306	KS 5, CCUG 51673	a	Adult periodontitis	Denmark	Own isolate (Aarhus, Denmark)
Aggregatibacter actinomycetemcomitans	no	no	yes	984	307	KS 6	a	Adult periodontitis	Denmark	Own isolate (Aarhus, Denmark)
Aggregatibacter actinomycetemcomitans	no	no	yes	986	309	PLN 6	a	Adult periodontitis	Denmark	Own isolate (Aarhus, Denmark)
Aggregatibacter actinomycetemcomitans	no	no	yes	987	310	PLN 7	a	Adult periodontitis	Denmark	Own isolate (Aarhus, Denmark)
Aggregatibacter actinomycetemcomitans	no	no	no	996	311	OMZ 249b	b	Unknown	Switzerland	B. Guggenheim (Zürich, Switzerland)
Aggregatibacter actinomycetemcomitans	no	no	no	997	312	OMZ 298, Tsai 652	c	Unknown	USA	B. Guggenheim (Zürich, Switzerland)
Aggregatibacter actinomycetemcomitans	no	no	no	998	313	OMZ 300, originated from ATCC 29523, CDC A1916	nt	Blood	USA	B. Guggenheim (Zürich, Switzerland)
Aggregatibacter actinomycetemcomitans	no	no	no	1000	314	EF 11145	b	Unknown	Sweden	E. Falsen /Göteborg, Sweden)
Aggregatibacter actinomycetemcomitans	no	no	no	1001	315	EF 11210	b	Unknown	Sweden	E. Falsen /Göteborg, Sweden)
Aggregatibacter actinomycetemcomitans	no	no	no	1002	316	EF 12604, CCUG 12604, HG3909, B 8092	d	Abscess	Sweden	E. Falsen /Göteborg, Sweden)
Aggregatibacter actinomycetemcomitans	no	no	no	1003	317	EF 13631	b	Unknown	Sweden	E. Falsen /Göteborg, Sweden)
Aggregatibacter actinomycetemcomitans	no	no	yes	1004	318	287-2	a	Oral cavity	Sweden	J. Carlsson (Umea, Sweden)
Aggregatibacter actinomycetemcomitans	no	no	no	1008	321	294-5	a	Oral cavity	Sweden	J. Carlsson (Umea, Sweden)
Haemophilus influenzae	no	no	no	1010	323	15292		Spinal fluid	Denmark	H. Schønheyder (Aalborg, Denmark)
Haemophilus influenzae	no	no	no	1011	324	15639		Cervix	Denmark	H. Schønheyder (Aalborg, Denmark)
Aggregatibacter actinomycetemcomitans	no	no	no	442	326	NCTC 9709, 120/48-49 (3)	c	Abscess	Denmark	NCTC
Aggregatibacter actinomycetemcomitans	no	no	yes	443	327	Type strain, ATCC 33384, CCUG 13227, DSM 8324, NCTC 9710, HG683, 277/49-50(8), 277/49-50, 2nd batch	c	Abscess	Denmark	NCTC
Aggregatibacter actinomycetemcomitans	no	no	no	666	328	147/80	b	Endocarditis	Sweden	I. Kallings (Stockholm, Sweden)
Aggregatibacter actinomycetemcomitans	no	no	yes	667	329	156/80	b	Blood	Sweden	I. Kallings (Stockholm, Sweden)
Arcanobacterium haemolyticum	no	no	no	1100	331	13702/92		Unknown	Denmark	Statens Serum Institut (Copenhagen, Denmark)
Haemophilus parainfluenzae	no	no	no	1101	332	14618/93		Unknown	Denmark	Statens Serum Institut (Copenhagen, Denmark)
Aggregatibacter actinomycetemcomitans	no	no	yes	1103	334	14409/93	a	Unknown	Denmark	Statens Serum Institut (Copenhagen, Denmark)
Haemophilus parainfluenzae	no	no	no	1104	335	13438/92		Unknown	Denmark	Statens Serum Institut (Copenhagen, Denmark)
Haemophilus parahaemolyticus	no	no	no	1114	342	HIM 712-6		Unknown	Germany	W. Mannheim (Marburg, Germany)
Aggregatibacter segnis	no	no	no	1116	344	Tillandeon		Unknown		
Haemophilus parainfluenzae	no	no	no	1121	348	HIM 955-7		Unknown	Germany	W. Mannheim (Marburg, Germany)

TABLE S4

Species	Cluster 10	Cluster 13	Cluster 25	HK No.	SPS No.	Other names	Serotype	Isolated from	Country	Source
Haemophilus parainfluenzae	no	no	no	1123	350	HIM 917-4		Unknown	Germany	W. Mannheim (Marburg, Germany)
Haemophilus parainfluenzae	no	no	no	1124	351	HIM 904-7		Unknown	Germany	W. Mannheim (Marburg, Germany)
Haemophilus parainfluenzae	no	no	no	1126	353	HIM 683-7		Unknown	Germany	W. Mannheim (Marburg, Germany)
Aggregatibacter actinomycetemcomitans	no	no	yes	1129	356	HIM 290-3	a	Unknown	Germany	W. Mannheim (Marburg, Germany)
Aggregatibacter actinomycetemcomitans	no	no	no	1132	359	HIM 294-3a	a	Unknown	Germany	W. Mannheim (Marburg, Germany)
Aggregatibacter aphrophilus	no	no	no	309	360			Dental plaque	Denmark	Own isolate (Aarhus, Denmark)
Aggregatibacter aphrophilus	no	no	no	310	361			Dental plaque	Denmark	Own isolate (Aarhus, Denmark)
Aggregatibacter aphrophilus	no	no	no	315	362			Dental plaque	Denmark	Own isolate (Aarhus, Denmark)
Aggregatibacter segnis	no	no	no	316	363	Type strain		Dental plaque	Denmark	Own isolate (Aarhus, Denmark)
Aggregatibacter aphrophilus	no	no	no	324	365	P527		Abscess	Denmark	W.W. Frederiksen, P. Holm (Copenhagen, Denmark)
Aggregatibacter aphrophilus	no	no	no	325	366	P536		Joint infection	Denmark	W.W. Frederiksen, P. Holm (Copenhagen, Denmark)
Aggregatibacter aphrophilus	no	no	no	326	367	P537		Sinusitis pus	Denmark	W.W. Frederiksen, P. Holm (Copenhagen, Denmark)
Aggregatibacter aphrophilus	no	no	no	329	368			Endocarditis	Denmark	Own isolate (Aarhus, Denmark)
Aggregatibacter aphrophilus	no	no	no	372	369	Type strain, ATCC 33389, CCUG 3715, NCTC 5906, 320		Endocarditis	UK	NCTC
Aggregatibacter aphrophilus	no	no	no	415	370	ATCC 29241, CCUG 41355, NCTC 10557, Funagli		Paronychia, human	UK	NCTC
Aggregatibacter segnis	no	no	no	499	371	Sims 47		Oral cavity	UK	W. Sims (London, UK)
Aggregatibacter segnis	no	no	no	500	372	Sims 22		Oral cavity	UK	W. Sims (London, UK)
Aggregatibacter segnis	no	no	no	574	373	Ampicillin res.		Oral cavity	Denmark	Own isolate (Aarhus, Denmark)
Haemophilus influenzae	no	no	no	705	374	MHAEM1052/SLCH628B	b	Blood	USA	J.M. Musser (Rochester, USA), D.M. Granoff (St. Louis, USA)
Haemophilus influenzae	no	no	no	706	375	MHAEM1053/SLCH808A	b	Blood	USA	J.M. Musser (Rochester, USA), D.M. Granoff (St. Louis, USA)
Haemophilus influenzae	no	no	no	708	377	HMAEM1055/SLCH1059A	b	Blood	USA	J.M. Musser (Rochester, USA), D.M. Granoff (St. Louis, USA)
Haemophilus parainfluenzae	no	no	no	758	379	269		Oro-pharynx	Brazil	I.M. Landgraf (Sao Paulo, Brasil)
Haemophilus parainfluenzae	no	no	no	759	380	400		Oro-pharynx	Brazil	I.M. Landgraf (Sao Paulo, Brasil)
Haemophilus parahaemolyticus	no	no	no	785	382	19314		Unknown	Denmark	Clinical Microbiology (Aarhus, Denmark)
Haemophilus pittmaniae	no	no	no	847	385	78/91		Blood	Brazil	M.C.C. Brandileone (Sao Paulo, Brasil)
Aggregatibacter actinomycetemcomitans	no	no	no	1095	388	543	b	Adult periodontitis	USA	S. Assikainen (Helsinki, Finland)
Aggregatibacter actinomycetemcomitans	no	no	no	1097	390	1155	b	Localized juvenile periodontitis	USA	S. Assikainen (Helsinki, Finland)
Aggregatibacter actinomycetemcomitans	no	no	yes	1099	392	2753	a	Adult periodontitis	Finland	S. Assikainen (Helsinki, Finland)

Table S4. Characteristics of studied strains.

Table consists of strains listed in rows and their characteristics in columns. The following information was provided for strains: species name; presence of prophages from clusters 10, 13, and 25; collection id numbers: HK, SPS, other; serotype; clinical origin; geographical origin; source of the strain.

TABLE S5

Name	F/R	Sequence* (5'–3')	Purpose	Reference
P1	R	TCTCCACCATTTTTGAGTGG	Serotyping, b, c, f	Kaplan et al., 2001
P2	R	GAAACCACTTCTATTTCTCC	Serotyping, b, c, f	Kaplan et al., 2001
P3	R	CCTTTATCAATCCAGACAGC	Serotyping, b, c, f	Kaplan et al., 2001
P4	F	ARAAYTTYTCWTCGGGAATG	Serotyping, b, c, f	Kaplan et al., 2001
P5	F	TGGGTCATGGGAGGTACTCC	Serotyping, a	Kaplan et al., 2001
P6	R	GCTAGGAACAAAGCAGCATC	Serotyping, a	Kaplan et al., 2001
P7	F	TGGAACGGGTATGGGAACGG	Serotyping, d	Kaplan et al., 2001
P8	R	GGATGCTCATCTAGCCATGC	Serotyping, d	Kaplan et al., 2001
P9	F	ATTCCAGCCTTTTGGTTCTC	Serotyping, e	Kaplan et al., 2001
P10	R	TGGTCTGCGTTGTAGGTTGG	Serotyping, e	Kaplan et al., 2001
P11	F	CTCAGAGATGGGTTTGTGCC	Serotyping, e'	Wahlfors et al., 1995
P12	R	AGATTCACTCCCCATCGCTG	Serotyping, e'	Wahlfors et al., 1995
P13	F	AGRGTTYGATYMTGGCTCAG	16S rRNA gene sequencing	Paliy et al., 2009
P14	R	RGYTACCTTGTTACGACTT	16S rRNA gene sequencing	Paliy et al., 2009
P15	F	TCTTGGTCGTGACGCTGTTA	*Aggregatibacter* Mu-like phage detection	this study
P16	R	GTGGCGATAATGCTCCAGTT	*Aggregatibacter* Mu-like phage detection	this study
P17	F	CTGGGCGAAAGCACACTTAG	*Aggregatibacter* Mu-like phage detection	this study
P18	R	CCGGCAAGATAACTGTCGAT	*Aggregatibacter* Mu-like phage detection	this study
P19	F	GCTTTGGTCGTGTTGTGACTA	*Aggregatibacter* Mu-like phage detection	this study
P20	R	GCACCAGGGCTTTTTCATAA	*Aggregatibacter* Mu-like phage detection	this study
P21	F	TGATGTGGTAAATTATGAACATTCG	*Aggregatibacter* B3-like phage detection	this study
P22	R	TTTCAAATAGGCTTCCGAGTTT	*Aggregatibacter* B3-like phage detection	this study
P23	F	CAAACTCCAAACCACCCATT	*Aggregatibacter* B3-like phage detection	this study
P24	R	TTCCACTGCGATTCTTGATG	*Aggregatibacter* B3-like phage detection	this study
P25	F	CGAGTTGAGCGTCGAAAACT	*Aggregatibacter* B3-like phage detection	this study
P26	R	AAGCTCTTTATCGCCGACCT	*Aggregatibacter* B3-like phage detection	this study
P27	F	GCAGGGTTTTGGTACTGGTC	*Aggregatibacter* AaΦ23-like phage detection	this study
P28	R	TTAACGGTTTCCGCATTTTC	*Aggregatibacter* AaΦ23-like phage detection	this study
P29	F	GTCTCTAACCGGTCAATTGGAA	*Aggregatibacter* AaΦ23-like phage detection	this study
P30	R	GTACAAGTGGATGCCGGTCT	*Aggregatibacter* AaΦ23-like phage detection	this study
P31	F	CAGATCAGCAAATCCAGCAA	*Aggregatibacter* AaΦ23-like phage detection	this study
P32	R	AACCAACGGAGGAATGTCAG	*Aggregatibacter* AaΦ23-like phage detection	this study

*R = A or G; Y = C or T; W = A or T.

Table S5. Primers used in the study.

References: (29-31)

File S1. Nucleotide sequences of prophage regions

See separate file.

File S2. Protein sequences of phage phylogenetic markers

See separate file.

References

1. Chen T, Yu WH, Izard J, Baranova OV, Lakshmanan A, Dewhirst FE. The Human Oral Microbiome Database: a web accessible resource for investigating oral microbe taxonomic and genomic information. Database : the journal of biological databases and curation. 2010;2010:baq013. Epub 2010/07/14.
2. Norskov-Lauritsen N, Kilian M. Reclassification of *Actinobacillus actinomycetemcomitans*, *Haemophilus aphrophilus*, *Haemophilus paraphrophilus* and *Haemophilus segnis* as *Aggregatibacter actinomycetemcomitans* gen. nov., comb. nov., *Aggregatibacter aphrophilus* comb. nov. and *Aggregatibacter segnis* comb. nov., and emended description of *Aggregatibacter aphrophilus* to include V factor-dependent and V factor-independent isolates. International journal of systematic and evolutionary microbiology. 2006;56(Pt 9):2135-46. Epub 2006/09/08.
3. Norskov-Lauritsen N. Classification, identification, and clinical significance of *Haemophilus* and *Aggregatibacter* species with host specificity for humans. Clinical microbiology reviews. 2014;27(2):214-40. Epub 2014/04/04.
4. Marks M, Fookes M, Wagner J, Ghinai R, Sokana O, Sarkodie YA, et al. Direct Whole-Genome Sequencing of Cutaneous Strains of *Haemophilus ducreyi*. Emerging infectious diseases. 2018;24(4):786-9. Epub 2018/03/20.
5. Pettigrew MM, Ahearn CP, Gent JF, Kong Y, Gallo MC, Munro JB, et al. *Haemophilus influenzae* genome evolution during persistence in the human airways in chronic obstructive pulmonary disease. Proceedings of the National Academy of Sciences of the United States of America. 2018;115(14):E3256-E65. Epub 2018/03/21.
6. Kittichotirat W, Bumgarner RE, Chen C. Evolutionary Divergence of *Aggregatibacter actinomycetemcomitans*. Journal of dental research. 2016;95(1):94-101. Epub 2015/10/01.
7. Bradley DE. Ultrastructure of bacteriophage and bacteriocins. Bacteriological reviews. 1967;31(4):230-314. Epub 1967/12/01.
8. Arndt D, Grant JR, Marcu A, Sajed T, Pon A, Liang Y, et al. PHASTER: a better, faster version of the PHAST phage search tool. Nucleic acids research. 2016;44(W1):W16-21. Epub 2016/05/05.
9. Paez-Espino D, Chen IA, Palaniappan K, Ratner A, Chu K, Szeto E, et al. IMG/VR: a database of cultured and uncultured DNA Viruses and retroviruses. Nucleic acids research. 2017;45(D1):D457-D65. Epub 2016/11/02.
10. Paez-Espino D, Eloe-Fadrosh EA, Pavlopoulos GA, Thomas AD, Huntemann M, Mikhailova N, et al. Uncovering Earth's virome. Nature. 2016;536(7617):425-30. Epub 2016/08/18.
11. Paez-Espino D, Pavlopoulos GA, Ivanova NN, Kyrpides NC. Nontargeted virus sequence discovery pipeline and virus clustering for metagenomic data. Nature protocols. 2017;12(8):1673-82. Epub 2017/07/28.
12. Paez-Espino D, Roux S, Chen IA, Palaniappan K, Ratner A, Chu K, et al. IMG/VR v.2.0: an integrated data management and analysis system for cultivated and environmental viral genomes. Nucleic acids research. 2019;47(D1):D678-D86. Epub 2018/11/09.
13. Shirley M, Ma Z, Pedersen B, Wheelan S. Efficient "pythonic" access to FASTA files using pyfaidx. PeerJ PrePrints 2015;3:e970v1
14. Brister JR, Ako-Adjei D, Bao Y, Blinkova O. NCBI viral genomes resource. Nucleic acids research. 2015;43(Database issue):D571-7. Epub 2014/11/28.

15. Anderson MJ, Gorley RN, Clarke KR. PERMANOVA+ for PRIMER: Guide to Software and Statistical Methods. PRIMER-E: Plymouth, UK.2008.

16. Gangaiah D, Webb KM, Humphreys TL, Fortney KR, Toh E, Tai A, et al. *Haemophilus ducreyi* Cutaneous Ulcer Strains Are Nearly Identical to Class I Genital Ulcer Strains. PLoS neglected tropical diseases. 2015;9(7):e0003918. Epub 2015/07/07.

17. Pillay A, Katz SS, Abrams AJ, Ballard RC, Simpson SV, Taleo F, et al. Complete Genome Sequences of 11 *Haemophilus ducreyi* Isolates from Children with Cutaneous Lesions in Vanuatu and Ghana. Genome announcements. 2016;4(4). Epub 2016/07/09.

18. Turnbaugh PJ, Ley RE, Hamady M, Fraser-Liggett CM, Knight R, Gordon JI. The human microbiome project. Nature. 2007;449(7164):804-10. Epub 2007/10/19.

19. Baltimore D. Expression of animal virus genomes. Bacteriological reviews. 1971;35(3):235-41. Epub 1971/09/01.

20. Federhen S. The NCBI Taxonomy database. Nucleic acids research. 2012;40(Database issue):D136-43. Epub 2011/12/06.

21. Lavigne R, Darius P, Summer EJ, Seto D, Mahadevan P, Nilsson AS, et al. Classification of Myoviridae bacteriophages using protein sequence similarity. BMC microbiology. 2009;9:224. Epub 2009/10/28.

22. Lefkowitz EJ, Dempsey DM, Hendrickson RC, Orton RJ, Siddell SG, Smith DB. Virus taxonomy: the database of the International Committee on Taxonomy of Viruses (ICTV). Nucleic acids research. 2018;46(D1):D708-D17. Epub 2017/10/19.

23. Roux S, Adriaenssens EM, Dutilh BE, Koonin EV, Kropinski AM, Krupovic M, et al. Minimum Information about an Uncultivated Virus Genome (MIUViG). Nature biotechnology. 2018. Epub 2018/12/18.

24. Roux S, Hallam SJ, Woyke T, Sullivan MB. Viral dark matter and virus-host interactions resolved from publicly available microbial genomes. eLife. 2015;4. Epub 2015/07/23.

25. Howard-Varona C, Hargreaves KR, Abedon ST, Sullivan MB. Lysogeny in nature: mechanisms, impact and ecology of temperate phages. The ISME journal. 2017;11(7):1511-20. Epub 2017/03/16.

26. Willi K, Sandmeier H, Meyer J. Temperate bacteriophages of *Actinobacillus actinomycetemcomitans* associated with periodontal disease are genetically related. Med Microbiol Lett. 1993;2:419-26.

27. Resch G, Kulik EM, Dietrich FS, Meyer J. Complete genomic nucleotide sequence of the temperate bacteriophage Aa Phi 23 of *Actinobacillus actinomycetemcomitans*. Journal of bacteriology. 2004;186(16):5523-8. Epub 2004/08/05.

28. Szafranski SP, Winkel A, Stiesch M. The use of bacteriophages to biocontrol oral biofilms. Journal of biotechnology. 2017;250:29-44. Epub 2017/01/22.

29. Kaplan JB, Perry MB, MacLean LL, Furgang D, Wilson ME, Fine DH. Structural and genetic analyses of O polysaccharide from *Actinobacillus actinomycetemcomitans* serotype f. Infection and immunity. 2001;69(9):5375-84. Epub 2001/08/14.

30. Wahlfors J, Meurman JH, Vaisanen P, Alakuijala P, Korhonen A, Torkko H, et al. Simultaneous detection of *Actinobacillus actinomycetemcomitans* and *Porphyromonas gingivalis* by a rapid PCR method. Journal of dental research. 1995;74(11):1796-801. Epub 1995/11/01.

31. Paliy O, Kenche H, Abernathy F, Michail S. High-throughput quantitative analysis of the human intestinal microbiota with a phylogenetic microarray. Applied and environmental microbiology. 2009;75(11):3572-9. Epub 2009/04/14.
